# Differences in nanoscale organization of regulatory active and inactive human chromatin

**DOI:** 10.1016/j.bpj.2022.02.009

**Published:** 2022-02-10

**Authors:** Katharina Brandstetter, Tilo Zülske, Tobias Ragoczy, David Hörl, Miguel Guirao-Ortiz, Clemens Steinek, Toby Barnes, Gabriela Stumberger, Jonathan Schwach, Eric Haugen, Eric Rynes, Philipp Korber, John A. Stamatoyannopoulos, Heinrich Leonhardt, Gero Wedemann, Hartmann Harz

**Affiliations:** 1 Human Biology & BioImaging, Faculty of Biology, Ludwig-Maximilians-Universität München, Munich, Germany; 2 Competence Center Bioinformatics, Institute for Applied Computer Science, Hochschule Stralsund, Stralsund, Germany; 3 Altius Institute for Biomedical Sciences, Seattle, Washington; 4 Biomedical Center (BMC), Molecular Biology, Faculty of Medicine, Ludwig-Maximilians-Universität München, Martinsried, Germany; 5 Department of Genome Sciences, University of Washington, Seattle, Washington; 6 Department of Medicine, Division of Oncology, University of Washington, Seattle, Washington

## Abstract

Methodological advances in conformation capture techniques have fundamentally changed our understanding of chromatin architecture. However, the nanoscale organization of chromatin and its cell-to-cell variance are less studied. Analyzing genome-wide data from 733 human cell and tissue samples, we identified 2 prototypical regions that exhibit high or absent hypersensitivity to deoxyribonuclease I, respectively. These regulatory active or inactive regions were examined in the lymphoblast cell line K562 by using high-throughput super-resolution microscopy. In both regions, we systematically measured the physical distance of 2 fluorescence in situ hybridization spots spaced by only 5 kb of DNA. Unexpectedly, the resulting distance distributions range from very compact to almost elongated configurations of more than 200-nm length for both the active and inactive regions. Monte Carlo simulations of a coarse-grained model of these chromatin regions based on published data of nucleosome occupancy in K562 cells were performed to understand the underlying mechanisms. There was no parameter set for the simulation model that can explain the microscopically measured distance distributions. Obviously, the chromatin state given by the strength of internucleosomal interaction, nucleosome occupancy, or amount of histone H1 differs from cell to cell, which results in the observed broad distance distributions. This large variability was not expected, especially in inactive regions. The results for the mechanisms for different distance distributions on this scale are important for understanding the contacts that mediate gene regulation. Microscopic measurements show that the inactive region investigated here is expected to be embedded in a more compact chromatin environment. The simulation results of this region require an increase in the strength of internucleosomal interactions. It may be speculated that the higher density of chromatin is caused by the increased internucleosomal interaction strength.

## Significance

Conformation capture techniques are limited to measuring contact probability. Here, we focused on a complementary aspect by measuring physical distances of loci, with a genomic distance of ∼5 kb in single cells. This range of distances of approximately 100 nm is crucial for mediating the physical contact of transcription factors and other regulatory elements. Microscopy data delivered the complete distance distribution of two prototypic regions with regulatory active and inactive chromatin, respectively. Unexpectedly, we found very broad distributions of distances in both regions. Computer simulations of a coarse-grained model of these regions showed that the variance of the single-cell measurements can be explained only by the combinations of different influencing factors. This emphasizes the large cell-to-cell variance in the processes regulating chromatin compaction even in inactive regions.

## Introduction

For almost 100 years, it has been known that interphase chromatin can be distinguished by means of light microscopy into less dense euchromatin and denser packed heterochromatin (1,2). Later, it became clear that nucleosomes are the basic building blocks organizing DNA packaging and are therefore central to the organization of chromatin (3). Groundbreaking electron microscopic studies showed the tight interaction between histones and DNA, forming an 11-nm-thick fiber (4,5). Methodological advances have led to the view that chromatin has a rather irregular, heterogeneous organization (6, 7, 8). This view is supported by electron microscopic studies and super-resolution fluorescence microscopy that show interphase chromatin to be organized in a flexible and disordered structure in which regions with higher nucleosome density are interspersed with nucleosome-depleted regions (9, 10, 11, 12, 13, 14).

The landscape of chromatin states is much more diverse than the originally described euchromatin and heterochromatin suggest. By analyzing genome-wide distribution patterns of chromatin-associated proteins, posttranslational histone modifications and DNase I hypersensitivity with algorithms such as ChromHMM and Segway, investigators have proposed up to 51 chromatin classes (15, 16, 17, 18, 19, 20, 21). DNase I hypersensitivity is a criterion that can also be used alone to subdivide chromatin in regulatory or active DNA with a high number of DNase I hypersensitive sites (DHS) as opposed to inactive regions with a low density of DHS (22,23).

Posttranslational histone modifications of the active chromatin classes, such as acetylation, may reduce nucleosome interaction strength and thus participate, among other mechanisms such as through ATP-dependent remodelers, in producing an open, less densely packed chromatin (24, 25, 26, 27, 28). Inactive classes are often characterized by methylation marks on histone 3 (e.g., H3K9me2/3), which can be bound by the heterochromatic protein 1, thereby compacting chromatin (29). However, large parts of inactive and more densely packed chromatin do not carry significant amounts of posttranslational histone modifications (15). Other mechanisms, such as the amount of linker histone H1, must therefore be responsible for the compaction (14).

A remarkable feature of chromatin is its dynamic nature, which has been observed in several fluorescence imaging studies (30, 31, 32, 33, 34, 35, 36, 37, 38, 39, 40) and is the reason for the large cell-to-cell variability in the structure of chromatin domains (41). Changes in nucleosome occupancy are actively regulated and can drastically affect the 3-dimensional (3D) genome architecture as it has been shown, for example, by the effects of tumor necrosis factor alpha on human endothelial cells (42). Even at the level of single nucleosomes, a significant and dynamic cell-to-cell variability can be found (43). The recently developed Fiber-seq method reveals that regulatory elements are actuated in an all-or-none fashion, thereby replacing a canonical nucleosome (44). Some ATP-dependent chromatin remodelers, and probably also some pioneer transcription factors, are known to exhibit nucleosome eviction activity (45, 46, 47). Together, these examples show that, depending on the regulatory context, the number and exact position of nucleosomes in active chromatin of eukaryotes can dynamically change.

Computational studies show a close link between nucleosome positions and the spatial organization of chromatin (48), which was explored by applying computer simulations of a coarse-grained model by many groups (e.g., (49, 50, 51)). These studies demonstrate, for example, that different nucleosome repeat lengths are responsible for more open or closed chromatin configurations (14,52). Moving even a single nucleosome can strongly influence the spatial organization (53). Thus, including the real length of the different linker DNA into coarse-grained models is required to obtain realistic results (14,53).

In our research, we investigated structural differences between active and inactive 5-kb chromatin segments of prototypical chromatin regions, selected on the basis of the presence or absence of DNase I hypersensitivity, using oligonucleotide-based fluorescence in situ hybridization (oligoFISH). By measuring the distance between labeled endpoints with systematic 3D stimulated emission depletion (STED) microscopy and comparing these data with Monte Carlo simulations of a coarse-grained model (53,54), we aimed to find underlying organizational principles. In active chromatin, simulated data match the microscopic data well, assuming cell-to-cell variability in nucleosomal occupancy. For inactive chromatin, variability of the maximal strength of the internucleosomal interaction and the binding of the linker histone H1 must be assumed to match the width of the distribution. Regardless of whether chromatin is active or inactive, our results reveal two striking features for 5-kb segments: (1) all distance distributions are right-tailed, and simulations indicate an underlying cell-to-cell variance in chromatin organization, and (2) distributions cover a wide range of distances from less than 50 nm to more than 200 nm.

## Materials and methods

For a more detailed description of the methods and procedures described in this section, please refer to the supporting materials and methods.

### Cell culture

Human erythroleukemia K562 cells (ATCC: CCL243) received from the Stamatoyannopoulos lab were grown in RPMI 1640 medium (Sigma, USA) supplemented with 10% fetal bovine serum (Sigma, USA) and 1% v/v penicillin/streptomycin (Sigma, USA) in cell culture flasks. Cells were cultured at 37°C in a humidified atmosphere containing 5% CO_2_ and regularly tested for mycoplasma contamination.

### Selection criteria for genomic regions

Universally active and inactive genomic regions were assessed by using the index of consensus DHSs of Meuleman et al. (22) derived from DNase I hypersensitive regions in 733 human biosamples encompassing 438 human cell and tissue types and states. We identified genomic regions with statistically significant enrichments of cleavage activity in DNase-seq experiments by using the program hotspot2 (55). The selected active region (chr11: 119,075,000–119,125,000) is spanned by a diverse set of genes, whereas the inactive region (chr11: 55,810,260–55,840,940) has a minimal number of elements overlapped by RepeatMasker.

### Sample preparation and microscopy

Oligonucleotide probes for STED microscopy: We tiled 30 non-overlapping oligonucleotides (40-mers) across each target region (1.5–2 kb), selected for uniqueness and a higher density than afforded by other published design tools optimized for whole-genome coverage or chromosome walking (56, 57, 58). Oligonucleotides were labeled with ATTO 594 or ATTO 647N (LGC Biosearch Technologies, USA). A list of all of the oligonucleotides used is provided in Table S1 in the supporting materials and methods. FISH of formaldehyde-fixed K562 cells was carried out as previously published (41) with adaptations.

STED microscopy was carried out on a 3D STED microscope (Abberior Instruments, Germany) equipped with 2 pulsed excitation lasers (594 nm, 0.3 mW and 640 nm, 1.2 mW), 1 pulsed depletion laser (775 nm, 1.2 W), and Avalanche photodiodes for detection. A 100× UPlanSApo 1.4 NA oil immersion objective (Olympus, Japan) was used for all of the acquisitions. Pairs of FISH spots labeled with different dyes were detected in confocal scans, and high-resolution STED detail stacks were acquired only around these points of interest.

### Image data analysis

Supervised machine learning was used as a quality control step to automatically classify STED stacks into “good” or “bad.” Detailed spot analysis was performed on the analyzable good data to determine the coordinates of both FISH spots in their respective STED channels. The algorithm searched for the spot pair with the brightest signal by using a Laplacian-of-Gaussian blob detector and saved their subpixel coordinates derived from fitting a multidimensional Gaussian using the Levenberg-Marquardt algorithm for further statistical analysis. For measurements with 2D depletion, 3D coordinates were transformed into projected 2D coordinates by omitting the z coordinate.

### Coarse-grained modeling

Simulation software: The software was developed in the Wedemann group in the last few decades and used in many studies. It was written in C++ and was adapted for the use of shared-memory parallel architectures according to the OpenMP standard. The replica exchange algorithm was implemented for distributed memory architectures using Message Passing Interface. The software cannot be made public at the moment, since it contains code under copyright by other parties.

Simulation protocol: A Monte Carlo (MC) algorithm was used to create a statistically relevant set of configurations satisfying the Boltzmann distribution (59). To overcome local energy minima (54), we applied a replica exchange procedure introduced by Swendsen and Wang (60). Here, *M* replicas of the system were simulated with Metropolis MC simultaneously, each at a different temperature, *T*_*i*_. After a fixed number of MC simulation step replicas with adjacent temperatures (*T*_*i*_*, T*_*i+1*_), the temperature is swapped with the probability (Eq. 1):

(1) $$\min\left[1,\exp\left(-\left(\beta_{i}-\beta_{i+1}\right)\left(E_{i+1}-E_{i}\right)\right)\right],$$

with $\beta_{i}=1/\left(k_{B}T_{i}\right)$, $k_{B}$ being the Boltzmann constant and $E_{i}$ the energy (e.g., elastic energies), of the system *i*. Before the simulations, the set of temperatures was determined using a feedback-optimized approach (61). This algorithm optimizes the distribution of temperatures iteratively, such that the diffusion of replicas from the highest to the lowest temperature and vice versa is improved in each iteration. The procedure is more efficient when starting with a system that is pre-relaxed using a simulated annealing approach (54). Simulation parameters and constants are given in Table S2.

We chose 16–60 replicas, depending on the system. For systems with 4 kT as a maximum value of internucleosomal interaction energy, we computed at least 10 × 10^6^ MC steps and 90 × 10^6^ steps for 6 kT per replica after simulated annealing. For checking the convergence, we analyzed the end-to-end distance and the energy as parameters. To determine the point when equilibrium was reached, we analyzed visually the plots with the number of steps on a logarithmic scale. Only configurations after that point were used in the analysis. From analysis of the autocorrelation of energy and end-to-end distance, we estimated that configurations are uncorrelated after 10 × 10^3^ steps for systems with 4-kT maximum interaction strength and 20 × 10^3^ steps for 6 kT (see supporting materials and methods). This leads to 1000–2000 uncorrelated configurations. See Table S3 for all of these values of every simulation in the supporting material.

Modeling of 3D configurations: Since atomistic modeling of chains with many nucleosomes is not possible, coarse-grained models are widely used. We applied the simulation procedure as described in Muller et al. and followed the description given there (53). Chromatin is modeled as a chain of segments, in which spherocylindrical units describing the nucleosomes are connected by cylindrical segments describing the linker DNA. Each segment *i* possesses a position and a local coordinate system consisting of three perpendicular unit vectors ($\hat{u_{i}},\hat{v_{i}},\;\hat{f_{i}}$) that describe its torsional orientation (Fig. S1). Vector $\hat{u_{i}}$ is parallel to the direction of the segment (i.e., the vector $\vec{s_{i}}$ from its position to the position of the next segment). The position of the center of the nucleosome and its orientation is computed from the center of the nucleosome segment by the length d and six angles describing the relative orientation (Fig. S2). Systems without linker histone and with linker histone differ by the set of angles (62). The length of each individual linker DNA was computed from the positions of the nucleosomes in the studied region. The number of base pairs of a linker length is converted to nanometers by a factor of 0.34 nm/bp. Each linker DNA is modeled by at least 2 segments. If the linker length is larger than 20 nm, then the number of segments is calculated by rounding up (linker length/10 nm).

### Statistical analysis

A mixture histogram (Fig. 4 *H*) was calculated by minimizing the squared differences between the bins of a histogram of the microscopically measured FISH spot distances (Fig. 4 *A*) and a linear combination of the histograms of simulation results with varying nucleosome occupancies (Fig. 4 *B–G*). Quadratic programming (via the quadprog package in R) was used to find a solution in which the contributions of the individual simulations are non-negative and equal 1.

2D and 3D distance data were cut off at the maximum length of a theoretical beads-on-a-string fiber, since it is very unlikely that genomic regions more elongated than a fully stretched beads-on-a-string fiber are present in the nucleus. To calculate the length of a beads-on-a-string fiber, the following formula was used: genomic length [bp] ^∗^ 0.34 nm (size of 1 base)/7 (63). For 5-kb genomic distances, the cutoff for measured distances was at 250 nm.

### Data availability

All of the data are available through the public Open Science Framework repository: https://doi.org/10.17605/OSF.IO/ZJWXM. Simulation trajectories are in an easily readable XML format (64). Analysis and visualization scripts are available as a directly runnable code ocean capsule: https://codeocean.com/capsule/8421512/tree/v2.

## Results

Chromatin organization of active and inactive chromatin was analyzed in K562 cells using systematic super-resolution microscopy of DNA sequences labeled with oligoFISH probes and comparison with simulated 3D chromatin configurations generated by a coarse-grained model. The K562 cell line is well suited to computer simulations as a wealth of information such as genome-wide chromatin immunoprecipitation sequencing data, comprehensive maps of posttranslational nucleosome modifications, and nucleosome positioning generated by the ENCODE project are available (21,65).

### STED microscopy as a tool to study prototypic chromatin regions on the kilobase scale

**Figure 1:**
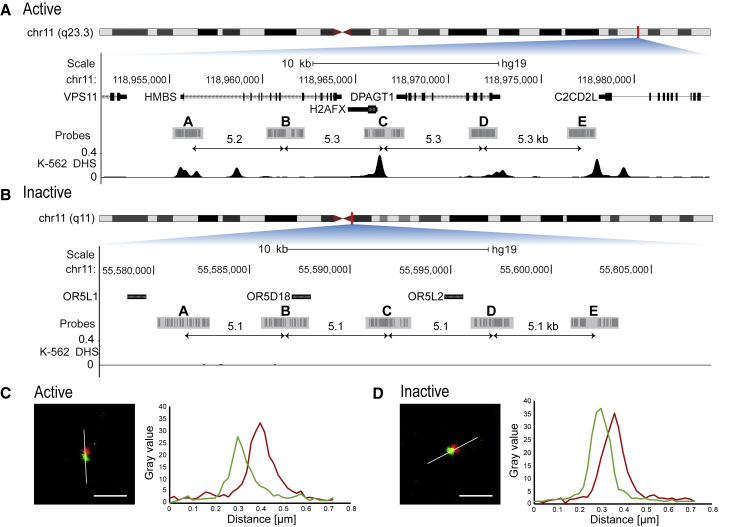
FISH probe design for active and inactive region. (*A*) The active region contains the genes *HMBS*, *H2AFX,* and *DPAGT1*. The probe sets are almost equally spaced (5.2, 5.3, 5.3, and 5.3 kb midpoint to midpoint) and mostly cover DHS sites. (*B*) The inactive region contains genes for olfactory receptors. The region shows no DNase I hypersensitivity, and the probe sets are equally spaced (5.1 kb midpoint to midpoint). Modified University of California, Santa Cruz genome browser plot (68); the data for the DNase I track are from GEO: GSM816655. (*C* and *D*) Representative STED detail images of FISH spots in 2 colors for active (*C*) and inactive (*D*) (target 1 in green, target 2 in red). Line plots depict intensity values for both colors along lines of interest (*white lines*). Scale bar, 500 nm. To see this figure in color, go online.

By using data from Meuleman et al. (22), we selected a 20-kb region on chromosome 11 (hg19, chr11: 118955404–118977871), which exhibits very high density of DHSs, not only in K562 (Fig. 1 *A*) but also in more than 730 samples from human cells and tissues. Moreover, this region is flanked upstream and downstream by highly active chromatin. For inactive chromatin, the selection criteria were a minimal number of repetitive elements and missing DHS over 30 kb in more than 730 human samples. In K562 cells, the region without DHSs spans over 2 Mb. The selected 20-kb inactive region is also located on chromosome 11 (hg19, chr11: 55580425–55603312) (Fig. 1 *B*). For each of these 20-kb regions, 5 oligoFISH probe sets (A, B, C, D, E; Fig. 1, *A* and *B*) were designed, dividing the 20 kb into 4 approximately 5-kb-long segments from midpoint to midpoint of the respective probe set (probe set combinations: AB, BC, CD, DE). Each oligoFISH probe set consisted of 30 oligonucleotides (directly fluorescently labeled 40mers) covering a region of approximately 1.5–2 kb (Fig. 1, *A* and *B*). These small genomic distances are expected to result in spatial distances falling below the resolution limit of light microscopy (66), which is more than 200 nm in the x- and y-dimensions and >500 nm in the z dimension (67). Using STED microscopy, we achieved a root mean square precision for the distance measurements between two spots with a different spectral behavior of approximately 7.5 nm in 3D (supporting materials and methods) (Fig. 1, *C* and *D*). By using reconstituted chromatin, we showed that STED microscopy can resolve distances between the ends of chromatin consisting of ∼5 kb DNA and up to 25 nucleosomes (Fig. S3). The distances measured microscopically were in the range of a simulated distance distribution of the same system.

### Inactive regions are more compact than active regions

**Figure 2:**
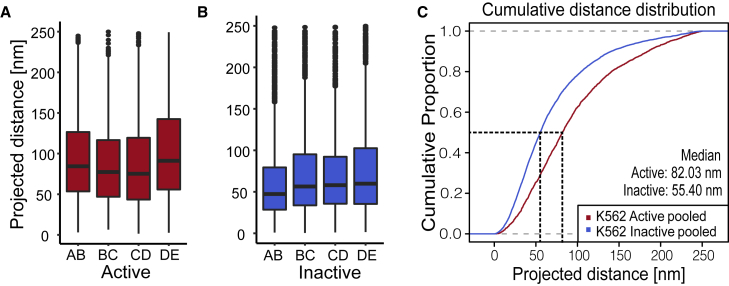
2D STED distance measurements showed increased compaction in the inactive region versus the active region. (*A*) Boxplot for the active region for all 4 measured intervals (AB: n = 672, BC: n = 540, CD: n = 484, DE: n = 566; n = number of single-cell measurements pooled from 3 independent replicates). (*B*) Boxplot for the inactive region for all 4 measured intervals (AB: n = 1585, BC: n = 1621, CD: n = 1200, DE: n = 1395; n = single-cell measurements from 3 independent replicates). (*C*) All of the data from active (*A*) and inactive (*B*) regions were pooled to generate a cumulative distribution. The cumulative distribution of measured distances showed differences in distributions between active (*red*) and inactive (*blue*). The median is the value at the 50% proportion (*black dashed line*). For the active region, the median is 82 nm and for the inactive region, it is 55 nm. To see this figure in color, go online.

Recent studies reveal a high cell-to-cell variance of the spatial genome organization (69, 70, 71). To study the chosen regions, we applied high-throughput 2D STED microscopy to generate data with high statistical power characterizing the nanoscale organization of 5-kb segments of active and inactive chromatin. For each of the 8 investigated 5-kb segments, between 484 and 1621 single-cell measurements were analyzed. The four measured intervals in the active chromatin region differ from one another. We found some significant deviations, with the maximum difference in the median projected distance of 16 nm (p = 0.00053, BC versus DE and CD versus DE, Wilcoxon rank-sum test) (Fig. 2 *A*; Table S4). In active chromatin, variability of the nanoscale organization is expected since each 5-kb segment is composed of different proportions of exons, introns, enhancers, and other regulatory sequences. Surprisingly, we also found highly significant differences between the investigated intervals in inactive chromatin. We expected much less difference in compaction because inactive chromatin is expected to be more uniform as it does not harbor active regulatory elements and nucleosome occupancy is not modified by transcriptional activity (Figs. 2 *B* and S4). The maximum difference in the median projected distance was 12 nm within the inactive chromatin group (p < 0.0001, AB versus DE, Wilcoxon rank-sum test; Table S4).

However, since the differences within the active and inactive regions are small, they were pooled to show the overall length distribution of each chromatin class. The median projected distance between 2 FISH spots flanking a typical 5-kb interval of active chromatin is 82 nm and 55 nm in inactive chromatin (Fig. 2 *C*). Shorter double spot distances indicate a higher degree of chromatin compaction, whereas larger distances suggest less compaction. Thus, data from our measurements are in line with published data showing active chromatin to be less compacted compared to inactive chromatin (11). As expected, the distributions of the FISH spot distances of active and inactive chromatin differ significantly as shown in a cumulative distribution plot (Fig. 2 *C*, p < 2 × 10^−16^, Wilcoxon rank-sum test; Table S4).

For a more in-depth analysis, we selected a 5-kb segment for both the active and inactive regions, which are representative of the respective group in 2D STED measurements. We chose interval AB for the active region and CD for the inactive region (Fig. 2, *A* and *B*). Both regions do not show CCCTC-binding factor (CTCF) binding sites and are therefore not anchors for chromatin loops.

### Assigning the input parameters for coarse-grained modeling

**Figure 3:**
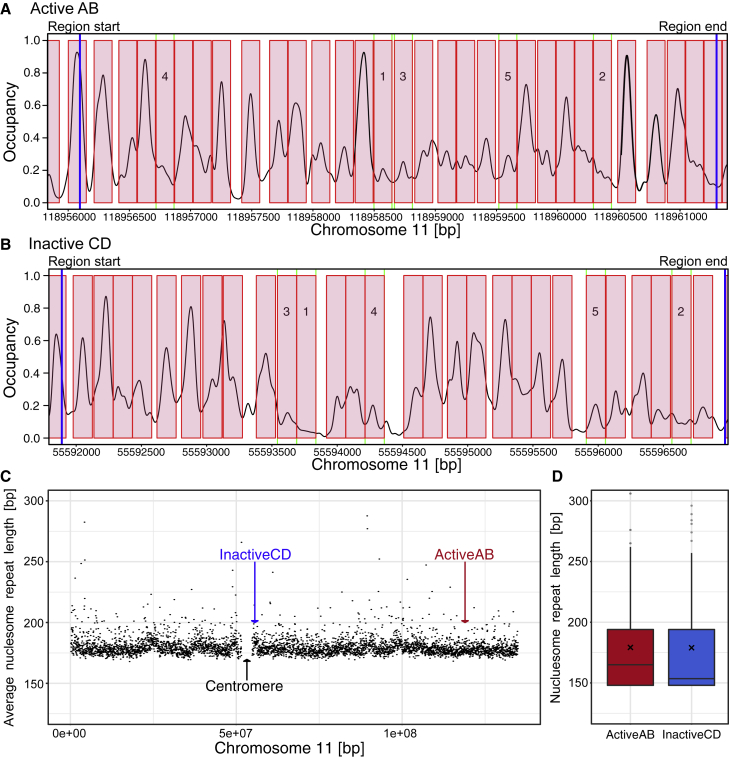
Nucleosome positions and nucleosome repeat lengths were calculated using the NucPosSimulator. Nucleosome positions (*red boxes*) for active AB (*A*) and inactive CD (*B*) were based on MNase-seq occupancy tracks (*black line*). The blue lines indicate the start and end of the investigated region. Numbers in boxes indicate the ranking of the 5 nucleosomes with the lowest nucleosome occupancy signal. (*C*) The mean values of the NRL of a sliding window of 30,000 bp. Values larger than 300 and windows with fewer than 3 nucleosomes were omitted. The mean NRL for chromosome 11 was 183.4 ± 66.3 bp. (*D*) Investigated active and inactive regions as marked in the plot (*arrows in C*) have a mean of 179.6 and 179.1 bp, respectively (black x). To see this figure in color, go online.

The exact position of nucleosomes is an important input parameter for coarse-grained models and strongly affects simulated configurations (14,53). Nucleosomal positioning can be determined by micrococcal nuclease digestion followed by deep sequencing (MNase-seq) (72). Here, we used ENCODE MNase-seq tracks of K562 cells, which are derived from cell populations and therefore often show a seemingly overlapping nucleosome pattern (University of California, Santa Cruz accession: wgEncodeEH000921, GEO accession: GSM920557). These data are unsuitable for our coarse-grained model, as it requires non-overlapping unique nucleosome positions as input. Therefore, we computed the most probable non-overlapping nucleosome populations by applying the NucPosSimulator (73). Experimentally derived nucleosome occupancy and the computed most probable nucleosome positions of active region AB and inactive region CD are shown in Fig. 3, *A* and *B*. Nucleosome positions of the respective flanking regions can be found in Fig. S4. We identified 28 nucleosomes in the active region and 29 nucleosomes in the inactive region. The flanking regions contain approximately 110 nucleosomes on each side. A list of the lengths of all linker DNA can be found in the Open Science Framework repository. For the nucleosomal repeat length (NRL) of chromosome 11, we calculated a mean value of 183.4 ± 66.3 bp applying NucPosSimulator (Fig. 3 *C*) (for calculation details, see the Materials and methods section). The mean NRL of the active (AB) and inactive (CD) region studied in detail is 179.6 and 179.1 bp, respectively (Fig. 3 *D*). Both values are in the range of the NRL of chromosome 11. To cross-check the effects of possible inaccuracies in the positions of the nucleosomes, additionally we determined the nucleosome positions for both regions and flanking regions from lymphoblastoid cell lines (74) and used them for control simulations.

The strength of the internucleosomal energy is another important parameter in all coarse-grained models and depends on the solvent (75) and histone modification (71). Literature values for this energy typically range from 3 to 10 kT (71,76,77). Nucleosomes containing unmodified histones have a higher interaction energy, whereas modifications such as acetylation weaken internucleosomal interactions (71). Since the inactive chromatin examined here does not exhibit significant histone modifications (Fig. S4 *B*), we have used a value from the upper range of the literature values (6 kT) to simulate this chromatin type. Conversely, the active region features many posttranslational histone modifications (Fig. S4 *A*), and we thus used a lower value (4 kT) to compute the respective configurations.

### The nucleosome occupancy varies from cell to cell in active chromatin

The microscopic data shown so far are 2D data, which underestimate the real 3D distances between the FISH spots since the cells are expected to be rotated randomly relative to the optical axis of the microscope. Only 3D single-cell microscopy allows the study of real distances between 2 spots on a single-cell level and to compare data between microscopy and simulation. Therefore, we performed 3D STED measurements, which require careful correction for refractive index mismatch between the immersion fluid of the objective lens and the embedding medium (see supporting materials and methods).

**Figure 4:**
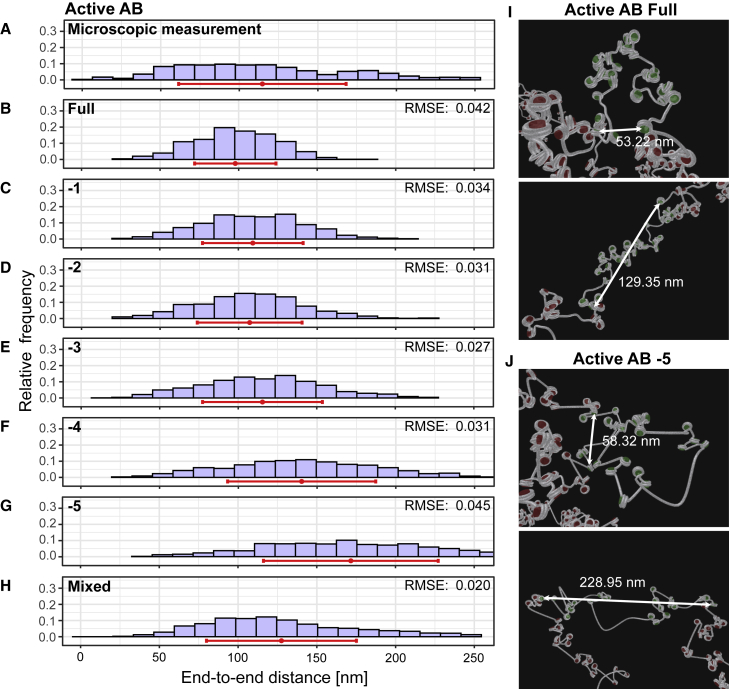
Distance distributions from microscopic experiments and from computer simulations of the active region. (*A*) 3D STED measurements of active AB result in a distance distribution ranging from <50 to 250 nm, with a mean of 115 ± 53 nm (n = 762 single-cell measurements from 3 independent replicates). (*B*–*H*) For computer simulations, results are shown for the region active AB with all nucleosomes (*full*) (*B*), with 1–5 nucleosomes replaced by naked DNA (*C–G*) and a combined plot (*H*). The mean value (*red dot*) and standard deviation (*red line*) are shown for each distribution. In the combined plot (*H*), the distributions have a weight of 0.45, 0.00, 0.00, 0.00, 0.31, and 0.24 (from all nucleosomes to −5 nucleosomes). (*I* and *J*) Example images of simulated chromatin fibers for active region AB (*green nucleosomes*), with all nucleosomes (*I*) and with −5 nucleosomes (*J*) and the adjacent sequences (*red nucleosomes*). The upper image in (*I*) and (*J*) shows a configuration resulting in a short end-to-end distance indicated by a white arrow; the lower image depicts a large end-to-end distance. RMSE, root mean square error of simulated histogram bins in comparison to the measured data. To see this figure in color, go online.

The 3D STED measurements for the 5-kb AB interval in the active chromatin region revealed distances ranging from <50 to 250 nm, with a mean distance of 115 nm (n = 762; Fig. 4 *A*; data of all other segments Fig. S5; statistical data in Table S5). Remarkably, in active chromatin, elongated configurations can be found, which results in a right-tailed distribution of the microscopic distance measurements. To understand this phenomenon better, we performed coarse-grained computer modeling of the nucleosome chain with the most probable nucleosome positions. We sampled a statistically relevant ensemble of independent 3D configurations in the active region by applying our coarse-grained model, which included elastic and electrostatic properties as well as excluded volume effects. To compare the simulated data with the microscopic data, the distances between the simulated sequence segments that correspond to those of the microscopic measurements were determined. In this way, a distance histogram was generated from the simulated data, which can be directly compared to the microscopic data (Fig. 4, *B–G*). The computed distribution was narrower, and the mean distance was approximately a standard deviation shorter than the microscopically measured distribution (Fig. 4 *B*).

We hypothesized that in the cell population used for the microscopy experiment, the number of bound nucleosomes varies from cell to cell. This hypothesis was tested by computer simulations, in which the least probable nucleosomes were removed. To find the nucleosomes with the lowest occupancy signal, we analyzed the mean value from the occupancy data calculated by NucPosSimulator (nucleosomes with lowest occupancy signal are indicated in Figs. 3 *A* and S4). Next, we computed statistically relevant ensembles of 3D configurations by replacing the nucleosome with the lowest occupancy signal by naked DNA (−1, Fig. 4 *C*). The same was done by replacing two (Fig. 4 *D*), three (Fig. 4 *E*), four (Fig. 4 *F*), and five (Fig. 4 *G*) nucleosomes according to the rank order of the nucleosome occupancy signal. In fact, a reduction of the total nucleosome number resulted in increasingly larger mean distances, but none of the individual distributions were comparable with the microscopically measured distribution. By applying a least squares fit, the different distance distributions were combined and resulted in a mixed distance histogram that mimics the microscopic data better than each of the underlying histograms, as indicated by a reduction in the root mean square error (Fig. 4 *H*) (see Materials and methods). Visualizations of simulated chromatin configurations show that both fibers with all nucleosomes and with a reduced nucleosome number (−5) can have short and long end-to-end distances (Fig. 4, *I* and *J*). These configurations show local accumulations of a few nucleosomes connected by stretches with low nucleosome occupancy. These structures are remarkably similar to recently published light and electron microscopic data of interphase chromatin (12,14,78). These results from the models are robust against possible inaccuracies in the nucleosome positions since computer simulations with nucleosome positions derived from lymphoblastoid cell lines deliver nearly identical results (Fig. S6, *K* and *L*). Linker histone H1 does not change the distance distributions in this case either (Fig. S6, *I* and *J*).

A process that is obviously accompanied by major changes in chromatin structure and in which nucleosomes are also temporarily removed from the chromatin structure is DNA replication in the S phase of the cell cycle. Fluorescence-activated cell sorting of cells with fluorescently labeled DNA was used to generate G1, S, and G2 phase fractions for further analysis (Fig. S7). Microscopically measured distance distributions of G1 and S phase cells resemble the data of the unsorted population.

### Inactive region is compacted by various mechanisms

**Figure 5:**
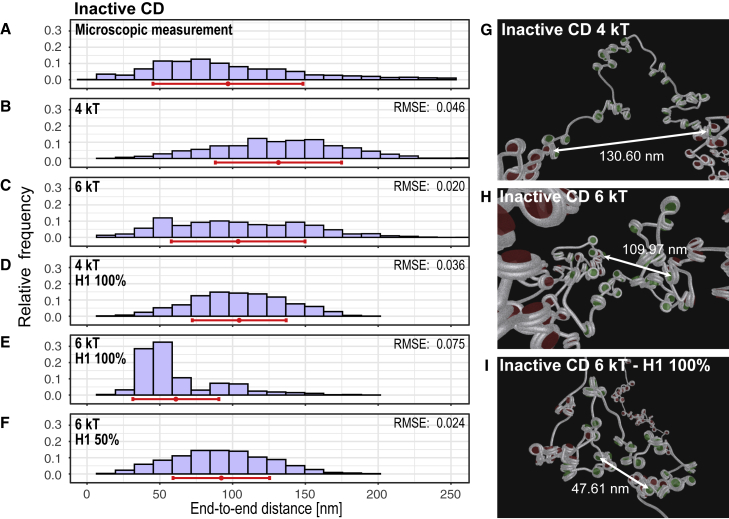
Distance distributions from microscopic experiments and from computer simulations of the inactive region. (*A*) 3D STED measurement of inactive CD results in a right-tailed distance distribution, with the mass of the distribution toward shorter distances and a mean of 97 ± 52 nm (n = 1320 single-cell measurements from 3 independent replicates). (*B*–*F*) Computed distance distributions with different maximal internucleosomal interactions (4 kT [*B*, *D*] and 6 kT [*C, E, F*]), without (*B*, *C*) linker histone H1 or with (*D*, *E*) H1 (100% of nucleosomes occupied), and a random distribution of binding of 50% H1 (*F*). The mean value (*red dot*) and standard deviation (*red line*) are shown for each distribution. (*G*–*I*) Visualizations of simulated configurations. RMSE, root-mean-square error of simulated histogram bins in comparison to the measured data. To see this figure in color, go online.

Microscopic data of the inactive region CD show the expected shift of the histogram to shorter distances, indicating more condensed chromatin (Fig. 5 *A*). Similar to the active chromatin, the histogram of the inactive region also contains large distances that cannot be explained by replicating DNA (Fig. S7). 3D STED distance histograms of the inactive region CD were compared with simulated data by the same strategy as above. The comparison showed that the computed mean distance was ∼40 nm larger than the microscopically measured distance when a maximal attractive internucleosomal energy of 4 kT was used for the simulation (Fig. 5, *A* and *B*). As argued earlier, an increase in the interaction energy to 6 kT seems to be more realistic for simulating inactive chromatin. This approach delivered configurations with the mean value of the simulated distance distribution in the correct range but symmetrical and not skewed to smaller values (Fig. 5 *C*). Obviously, additional mechanisms compact the inactive chromatin of the investigated region.

Genome-wide data on the level of H3K9me3 (GEO: GSM733776) and H3K27me3 (GEO: GSM733658) histone modifications show in the inactive region CD only background levels, which can be found throughout the genome. Also, repetitive DNA sequences (RepeatMasker) are not enriched. The levels of these markers are significantly lower than in regions known to be compacted by heterochromatinization or by binding the Polycomb group proteins. Therefore, other mechanisms must be considered, such as the binding of linker histone 1 (H1), which has long been known to have a chromatin-compacting effect (14,79). H1 is included in the computer model by different angles of the attached linker DNA at the nucleosomes (52). These angles were derived by a systematic analysis of data from reconstituted fibers (62). It can be expected that details of the angles vary since the chicken linker histone H5, for example, causes different angles than human H1 (62). However, all of the variants of H1 lead to higher chromatin compaction.

In fact, simulations with a stochiometric H1 to nucleosome ratio of 1:1 led to more compact configurations, with a mean value of 27 nm less for 4 kT (Fig. 5 *D*) and 43 nm less for 6 kT (Fig. 5 *E*). To explore the effects of different stoichiometry of H1, we performed computer simulations of a random 50% nucleosome binding (1:2). Here, the compaction is less pronounced, and the mean is approximately 12 nm smaller than without H1 (Fig. 5 *F*). Visualizations of exemplary simulated configurations are shown in Fig. 5, *G–I*.

In summary, similar to the active region the experimental data of the inactive region can only be explained by a cell-to-cell variability but including a stronger internucleosomal interaction and binding of H1.

## Discussion

By using high-throughput super-resolution microscopy, we studied the nanoscale organization of 5-kb chromatin segments that are located in regulatory active and inactive chromatin. The data shown here contain information that differs for fundamental reasons from that of published conformation capture data sets such as Hi-C. While microscopy measures the physical distances between genomic loci, conformation capture methods assess how often direct contacts between genomic elements occur (80). Most conformation capture data sets represent a population average, whereas we provide here statistically robust data on the chromatin configuration in single cells that can be directly compared with data from simulations. The selected areas are prototypic for active and inactive chromatin because patterns of prominent or absent DHSs spread over hundreds of kilobases around the selected region and can be found in more than 730 different human cell and tissue samples. Both regions have an NRL close to the average of the entire chromosome, which is another indication that representative regions were selected. For these reasons, we assume that the structural principles described apply to other parts of the genome.

In both active and inactive chromatin, 3D spatial distances between the endpoints of the 5-kb segments differ from cell to cell, resulting in a broad distance distribution, with the mass of the distribution shifted more to shorter values in inactive chromatin. In contrast, simulations with different nucleosome occupancies, changed strength of the internucleosomal energies, or deviations from stoichiometric H1 binding led to far narrower distance distributions. Therefore, the large width of the distance distribution seems to be a feature that is caused by the summation of cell-to-cell differences in the resulting histogram.

Unexpectedly, we found very elongated chromatin configurations with 5-kb exhibiting lengths of over 200 nm in both active and inactive chromatin. For comparison, a stretched beads-on-a-string chromatin fiber of 5 kb has a length of 243 nm (63). Replication cannot account for the majority of these elongated configurations as we have shown by measurements on cells in G1 phase. Replication is a fast process, occurring at ∼16 bp/s (81), so the probability of fixing a cell at the moment a 5-kb segment is currently replicated is low. The same is true for other active mechanisms such as loop extrusion by cohesion (82). In simulations with our coarse-grained model, elongated chromatin configurations are more probable if a number of nucleosomes are replaced by naked DNA. Therefore, it is important to investigate which nucleosomes have the weakest occupancy in our model. In fact, 8 of the 10 nucleosomes with the lowest occupancy in the active region are localized within DHSs (Fig. S4 *A*), a result that is consistent with genome-wide measurements (44).

The perspective of cell-to-cell differences in nucleosome occupancy in active DNA is supported by different lines of evidence: (1) While at certain positions nucleosomes are positioned with high precision (83), nucleosome positions can vary substantially from cell to cell (43,73), (2) pioneer transcription factors and chromatin remodeling complexes can change nucleosome occupancy (84,85), (3) the upregulation of genes is known to reduce the number of bound nucleosomes (42) and increases H2B mobility (14), (4) transcription factors compete cooperatively with nucleosomes for access to DNA (86,87), and (5) regulatory elements are actuated in an all-or-none fashion by the cooperative binding of transcriptional factors in place of a canonical nucleosome (44,88).

For most of the 1600 known transcription factors (89), there are no models to estimate the DNA structure after binding. We simulated regions without nucleosomes as linker DNA with the corresponding elastic and electrostatic properties, since 92% of the transcription factors studied have a DNA footprint between 7 and 30 nt (90), while nucleosomes have a footprint of 146–147 nt and the DNA is 1.65× wrapped around them (5). Therefore, we can neglect bound transcription factors in the model without limiting the conclusions.

As described earlier, the microscopic measurements of inactive chromatin revealed a compaction that can be explained by an increase in the strength of internucleosomal interactions and by the additional introduction of the linker histone H1. Microscopic measurements showed that the inactive region investigated here is expected to be embedded in a more compact chromatin environment (Fig. S8). It can be speculated that this higher density is caused by the increased internucleosomal interaction strength as found in the model. This result is in line with the current discussion of phase separation in the nucleus (91).

Similar to the active regions, microscopic data of the inactive region also show elongated chromatin configurations (>200 nm) in individual cells. In analogy to active chromatin, this could indicate variable nucleosome occupancy in inactive chromatin as well. In fact, the data shown in Fig. 3 *B* also show weakly bound nucleosomes in this chromatin class, but this does not exclude further mechanisms causing elongated chromatin configurations. Regardless, the large variation in physical distances between spots with a genomic distance of 5 kb from cell to cell suggests that inactive chromatin is also subject to continuous reorganization.

In each computer simulation, a system has a certain number of nucleosomes, amount of bound H1, and internucleosomal interaction strength. It can be expected that *in vivo* more variety exists (e.g., in the active region, one or two nucleosome are missing and a varying stoichiometry of linker histone is present). The properties of these systems are expected to be in the range of the already-broad range of different simulated systems presented here.

An extensive body of literature (for a review, see Schoenfelder and Fraser (92)) on chromatin architecture focuses on the formation of chromatin loops, bringing regulatory elements into close contact and thus regulating gene expression. Distances below which an enhancer is thought to activate a promotor range from less than 150 nm (66) to 300 nm (93). Here, we show by high-throughput microscopy of human chromatin that in active regions more than 45% of the 5-kb endpoints approach to less than 100 nm, whereas in inactive chromatin, this is the case in more than 60% of the cells (value derived from data of Figs. 4 *A* and 5 *A*). Apparently, thermodynamically driven spontaneous movements can bring regulatory elements into close contact with their promoters that are only a few kilobases distant from one another. Considering that in the human genome, 142,000 enhancer-like elements fall within 2 kb from the nearest transcription start site (21), such spontaneous movements of chromatin could significantly influence gene regulation.

## Author contributions

This study was conceived and supervised by H.H., G.W., J.A.S., and H.L. K.B. performed all of the microscopic experiments, including sample preparation and STED imaging shown in Figs. 1, 2, 4 *A*, 5 *A*, S5, and S8. Work on reconstituted chromatin (Fig. S3) was completed by C.S., who was responsible for the molecular biology; T.B. prepared the salt gradient dialysis chromatin under the supervision of P.K.; D.H., M.G.-O. and C.S. performed the imaging. Cell-cycle measurements (Fig. S7) were performed by M.G.-O. and G.S. (imaging) and J.S. (fluorescence-activated cell sorting). E.R. selected the investigated genomic regions, and E.H. provided the computational tools for the oligo probe design. T.R. designed the oligo probes and supervised the FISH experiments. K.B. analyzed and interpreted the published ENCODE genome browser data, with the assistance of T.R. (Fig. S4). D.H. provided the computational tools for microscope automation and image analysis. T.Z. performed the computational modeling, with input from G.W. T.Z. analyzed the nucleosome position from MNase data, prepared and executed all of the simulations, and analyzed the simulation data as 3D visualizations and histograms (Figs. 3; 4, *B*–*J*; 5, *B*–*I*; S1; S2; S3, *D*; and S6, *B*–*L*). The manuscript was written by H.H. and G.W., with support from all of the authors.

## Supporting citations

References (94, 95, 96, 97, 98, 99, 100, 101, 102, 103, 104, 105, 106, 107, 108, 109, 110) appear in the supporting material.

## Supporting material

Document S1. Tables S2–S5 and Figures S1–S8

Table S1. Genomic coordinates and sequences of the used oligonucleotide probes

Document S2. Article plus supporting material
