# Supplementary material for: Differences in nanoscale organization of regulatory active and inactive human chromatin
Source: Biophys J. 2022 Feb 10;121(6):977–90. doi: 10.1016/j.bpj.2022.02.009 (PMC8943813; doi:10.1016/j.bpj.2022.02.009)
Supplement: Document S1. Tables S2–S5 and Figures S1–S8 [file mmc1.pdf]

Biophysical Journal, Volume *121*

Supplemental information

Differences in nanoscale organization of regulatory active and inactive human chromatin

Katharina Brandstetter, Tilo Zülske, Tobias Ragoczy, David Hörl, Miguel Guirao-Ortiz, Clemens Steinek, Toby Barnes, Gabriela Stumberger, Jonathan Schwach, Eric Haugen, Eric Rynes, Philipp Korber, John A. Stamatoyannopoulos, Heinrich Leonhardt, Gero Wedemann, and Hartmann Harz

SUPPORTING MATERIALS AND METHODS

Reagents

Dulbecco's Phosphate buffered saline (DPBS, 1x, D8537), Poly-L-lysine (P1399), Dextran Sulfate (D6001) and DAPI (D9542) were all purchased by Sigma, Germany. 10x DPBS (Gibco, 14200075) and 20x SSC (Invitrogen, AM9763) were from Thermo Fisher Scientific, USA. Furthermore 16% methanol-free and ultra-pure Formaldehyde (18814-20, Polysciences, USA), high-precision coverslips of 1.5 thickness and 18x18 mm size (LH22.1, Carl Rot, Germany), Formamide (0606, Amresco, USA), Fixogum rubber cement (Marabu, Germany) and SiR-DNA dye (SC007, Spirochrome, Switzerland) were used. MOWIOL was prepared according to (1).

Selection criteria for genomic regions used in this study

From the UCSC Genome browser (http://hgdownload.soe.ucsc.edu/goldenPath/hg38/database/), we downloaded the hg38 coordinates of centromeres, segmental duplications ("super dups"), and short tandem repeats ("RepeatMasker"). From the Gencode Genes project website, we downloaded version 37 of "basic" gene annotations for the 24 chromosomes in hg38 coordinates (ftp://ftp.ebi.ac.uk/pub/databases/gencode/Gencode_human/release_37/gencode.v37.basic.annotation.gtf.gz). From the Gencode file, we extracted the coordinates of gene bodies for genes annotated at level 1 or 2 for which at least one transcript is annotated at level 1 or 2 with a transcript support level of 1 or 2; this curated list of gene bodies comprises the "genic regions" in what follows. To assess chromatin accessibility across diverse cell and tissue types, we used the "Index" of (2) derived from DNase I hypersensitive regions called at FDR 0.1% in 733 diverse biosamples, and to assess accessibility in K-562 cells, we used regions called via the program hotspot2 (3) at FDR 0.1% from the alignment file downloadable from https://www.encodeproject.org/files/ENCFF591TEM/.

To identify inactive regions, we took all genomic regions between successive elements in the Index, and the regions between the first and last Index elements on each chromosome and their respective ends of the chromosome. From these, we subtracted centromeric and genic regions, and retained all resulting regions with widths of at least 25 kb across which over 80% of the sites are uniquely mappable by 36mers and over 80% lie outside regions of segmental duplications. From these remaining regions, we chose the one that was overlapped the least by RepeatMasker elements. This region, chr11:55,810,260-55,840,940, stood out because less than 26.9% of it is overlapped by RepeatMasker elements; over 62% of each of the remaining candidate inactive regions are overlapped by RepeatMasker.

To identify active regions, we started by partitioning each chromosome into 50-kb segments, starting at the "left" end of each chromosome, and later repeating this 50-kb partitioning with an offset of 25kb into each chromosome. We restricted the 50-kb segments to those that are at least 50% overlapped by Index elements and overlapped <35% by RepeatMasker elements and not overlapped by any segmental duplications. We further restricted these to 50-kb regions fully containing an Index element present in 732 or 733 diverse biosamples and a strong (maximally-scoring) DNase I hypersensitive region in the K-562 biosample. We ranked the remaining 50-kb segments in descending order by the percentages by which they are overlapped by Index elements and considered their genic content and the degree to

which they are overlapped by RepeatMasker elements. The region chr11:119,075,000-119,125,000 stood out for being spanned by a diverse set of genes.

In addition, the probe sets were spaced approximately 5 kb (midpoint to midpoint) from each other and, for the active region, were mostly placed on DHS peaks. The probe sets were designed to span 1.5 – 2 kb.

The probe sets were also transferred to the hg19 genome assembly by using the UCSC genome browser in order to be able to use the publicly available MNase-seq data set ENCSR000CXQ from the ENCODE project (see also Preparation and Simulation).

Sample preparation and microscopy

Oligonucleotide probes for STED microscopy. Dye conjugation was carried out post-synthesis in a pool via an NHS-ester modification reaction. Working stocks of pools of 30 oligonucleotides covering the target regions had a total concentration of 10 µM and were diluted further for experiments. For a list of all oligo probes used in this study see Table S1.

Sample preparation and fluorescence in situ hybridization (FISH). Hybridization was carried out as previously published with small adaptations (4). PBS washed K-562 cells were resuspended in a small volume of PBS at a density of 1 million cells per ml and cell suspension was applied to poly-L-lysine coated glass coverslips. Cells were fixed using an osmotically balanced and methanol-free 4% formaldehyde solution which has previously been shown to not cause detectable nuclear shrinkage (5). The following washing and permeabilization steps were carried out according to Bintu et al (4). Coverslips were then inverted onto 8 µl of hybridization solution and sealed with rubber cement. Slides were placed on a heat block set to 81°C for 3 minutes. The samples were incubated at 37°C overnight (16 – 20 h). This protocol uses low hybridization temperatures and short hybridization times which has previously been shown to only minimally disrupt chromatin structure on the nanoscale by using electron and super-resolution microscopy (5-8). Due to the directly labeled primary probes the protocol contains only washing steps on the second day. The samples were washed twice with 2x SSC for 15 minutes. Two 7-minute washes in 0.2x SSC/ 0.2% Tween-20 were carried out on a heat block at 56°C followed by one wash in 4x SSC/ 0.2% Tween-20 at RT. DNA was counterstained with DAPI (100 µg/ml in 2x SSC), followed by two more washes in 2x SSC. Coverslips were mounted on microscopic slides with MOWIOL (2.5% DABCO, pH 7.0), dried for 30 minutes and sealed with nail polish to preserve cell morphology and prevent shrinkage of cells.

Sample preparation for FISH and SiR-DNA staining. Samples were prepared the same way as for two color FISH. In this case only one probe pool (B for active and inactive region) with an ATTO 594 dye label was used for hybridization. Instead of DAPI counterstaining, the samples were stained in 2.5 µM SiR-DNA in 2x SSC for 1 h in a humid chamber. Subsequently, slides were washed two times with 2x SSC for 5 minutes. Coverslips were mounted on microscopic slides with MOWIOL (2.5% DABCO, pH 7.0), dried for 30 min and sealed with nail polish.

Fluorescence activated cell sorting (FACS) The effect of the cell cycle stage on the chromatin configuration was investigated by sorting K562 cells based on their DNA content into the G1, S and G2 phase. Cells were incubated for 30 minutes in RPMI 1640 medium (10 % FCS, 1 % strep/pen) containing 5 µg/µl Hoechst33342 (Thermo Scientific, Germany). After centrifugation cell pellets were resuspended in 0.5 % BSA/PBS and sieved (mesh size 40 µm) to remove cell clumps. Cell sorting was performed using the BD FACSAria Fusion (BD Biosciences, New Jersey) and FACS Diva 9.0.1 software. The gating of the sorter was adjusted to sort single cells based on light scattering. Fluorescent gates were set to sort G1, S and G2 phase cells based on Hoechst33342 fluorescence using a 405nm laser excitation and a 425-475nm bandpass emission filter. Subsequently, the three cell fractions were used in FISH experiments as described above.

STED microscopy for FISH two color imaging. The STED hardware was controlled with Python scripts by using the specpy interface to the microscope control software Imspector (versions 0.13 and 14.0, Abberior Instruments). To find oligoFISH spot pairs confocal dual color 50 µm x 50 µm x 5 µm (for 2D and 3D acquisitions) volumes were acquired using 100 µm pinhole, 150 nm pixel size, 250 nm z-steps, 10 µs pixel dwell time, no line accumulation and excitation laser powers of 18.8% for 594 nm and 19.3% for 640 nm. Confocal scans were investigated, points were detected with a Laplacian-of-Gaussian blob detector in both channels and nuclear regions exhibiting signals in both color channels no more than 5 pixels apart from one another were determined. At these points of interest, STED detail stacks (3 µm x 3µm x 1.4µm) were acquired. For 2D STED acquisitions, the spatial light modulator (SLM) was used to generate a 2D STED depletion pattern and stacks were acquired with 200 nm z steps, 7 planes, 20 nm pixel size, 10 µs pixel dwell time, 5x line accumulation, 100 µm pinhole, excitation laser power 53.5% for 594 nm, 53.5% for 640 nm and 29.6% for 775 nm depletion laser power. For 3D STED acquisitions, careful correction for refractive index mismatch between immersion fluid of the microscope objective and the cell is crucial. Therefore, immersion oil with a refractive index of 1.522 was used for 3D acquisitions. The SLM modulator was set to generate a 3D STED depletion pattern and stacks (3 µm x 3 µm x 1.5 µm) were imaged with 60 nm z steps, 25 planes, 45 nm pixel size, 10 µs pixel dwell time, 5x line accumulation, 100 µm pinhole, excitation laser power 53.5% for 594 nm, 53.5% for 640 nm and 29.6% for 775 nm depletion laser power. The process was repeated for the next overview scan. The focus position was updated to the plane of maximum intensity in the previous overview image to allow for overnight imaging without focus loss. By moving the stage in x and y in a spiral pattern, overview scans followed by STED detail scans were acquired until a pre-set amount of time had passed.

STED microscopy for FISH and SiR-DNA co-imaging. Image acquisitions were carried out on a 3D STED microscope system from Abberior Instruments described above using a 100x UPlanSApo 1.4 NA oil immersion objective (Olympus, Japan). The STED hardware was controlled with Python scripts as described above. To find oligoFISH spots in 594 nm confocal dual color 50 µm x 50 µm x 7 µm volumes were acquired using 100 µm pinhole, 150 nm pixel size, 10 µs pixel dwell time, no line accumulation and excitation laser powers of 18.8% for 594 nm and 19.3% for 640 nm. Confocal scans were investigated, points were detected with a Laplacian-of-Gaussian blob detector in the 594 nm channel. At these points of interest, STED detail stacks (3 µm x 3 µm x 1.4 µm, 200 nm plane spacing) were acquired using the 594 nm laser for excitation. To get the surrounding SiR-DNA signal a 15 x 15 µm (30

nm pixel size, 1 plane) field of view was acquired around the same points of interest using the 640 nm laser. By moving the stage in x and y in a spiral pattern, overview scans followed by STED detail scans were acquired until a pre-set amount of time had passed.

Measurement of the microscopic Resolution

Precision of distance measurement

As STED microscopy is not prone to chromatic aberrations (9) we implement the two-color approach to increase optical resolution (10) without chromatic correction. In order to estimate the localization precision under our imaging conditions we proceeded as follows:

1. As measuring the localization precision is challenging in cells because FISH spots bleach too quickly, we used a test sample with bleach-stable fluorescent beads (Tetra Spec, dark red, 100nm, Thermo Fischer, USA).
2. The intensity of the excitation and depletion laser was adjusted so that the images of the beads are comparable to the FISH spots in the publication. By acquiring *T*=10 consecutive 3D stacks of the same beads, image series were obtained in which the beads were localized by a Laplacian of Gaussian blob detection followed by a least squares fit of a Gaussian function.
3. We determined the relative position $x_{i,t}^{r}$ of each bead *i* relative to the center of mass of all beads in one frame *t* of the timeseries to mitigate effects of drift (which would not be present in the single-frame FISH measurements).
4. We then calculated the mean relative position of individual beads over time μx_i^r and calculated the root-mean-square deviation (*RMSD*) for each bead:
$$RMSD_i = \sqrt{\frac{1}{T}\sum_{t=1}^{T}(x_{i,t}^{r} - \mu x_i^r)^2}$$
In 3D depletion mode, we measure a mean *RMSD* of 5.3nm (xyz).
5. By assuming that the errors are uncorrelated the localization imprecision for the 3D distance of a pair of spots results in $\sqrt{2}\,5.3\ \approx 7.5$ nm

Measurement of microscope resolution with reconstituted chromatin

The plasmid pFMP233 (11) contains 25 repeats (197-bp) of the Widom 601 sequence (25 x 601). This insert was excised by EcoRI and Xba1 digestion and purified by agarose gel electrophoresis. The ends of the 25-mer were labeled by ligation with 58 bp long oligonucleotides marked by 5-Propargylamino-dCTP-ATTO-647N (Jena Bioscience) or 5-Propargylamino-dCTP-ATTO-594 (Jena Bioscience) respectively. In the next step, the labeled 25 x 601 fragments were either used for microcopy or assembled into chromatin by salt gradient dialysis as described (12). This was done at medium assembly degree (histone: DNA mass ratio ~ 0.5) by using a mixture (~ 1:1 ratio) of labeled and unlabeled DNA. Reconstituted chromatin or labelled 25 x 601 fragments were mounted for STED microscopy on poly-D-lysin coated coverslips. After washing and fixing in 4% formaldehyde, samples were mounted for STED microscopy as described above (Fig. S3).

Modeling of reconstituted chromatin

For the 25x601 systems used for controls base pair 88 to 234 was the position of the first nucleosome. The nuclear repeat length for the fragment was 197 bp. Therefore, the nucleosomes start points were calculated as 88 + x * 197 bp and the end points as 234 + x * 197 bp with x as an integer between 0 and 24. We performed simulations with a maximal internucleosomal interaction of 4 kT and 6 kT. We estimated that configurations are uncorrelated after $2.5x10^3$ steps for the 25x601 system used for controls (s. section "Analysis of correlation of simulated configurations"). We sampled 2000 independent configurations. The histograms of the end-to-end distances seem quite symmetric (Fig. S3). Distances are in the range of 0 to 260 nm with a peak at about 140 nm. For increasing the internucleosomal interaction strength the compaction rises only by a negligible distance for this system.

Image data analysis

STED microscopy image analysis for FISH spot distances. Though the automated data acquisition process produced large numbers of images, some of these were of insufficient quality for further analysis due to poor signal to noise ratio or spot detection only in one channel caused by premature bleaching or sample drift. Therefore, supervised machine learning was used as a quality control step to automatically classify STED stacks into "good" or "bad". An experienced scientist classified about more than two thousand sum projections of oligoFISH STED stacks as "analyzable data" or "not analyzable data". Features extracted from the sum projections of his ground truth dataset were used to train a Random Forest classifier that could be used to automatically classify further acquisitions. All machine learning was done in Python 3 (13) using scikit-learn (ver. 0.19.1 or earlier). All acquired raw data including "good" and "bad" images can be found via DOI 10.17605/OSF.IO/ZJWXM

Detailed spot analysis was performed on the analyzable data to determine the coordinates of both FISH spots in their respective STED channels. The algorithm searched for the spot pair with the brightest signal and saved their subpixel coordinates for further statistical analysis. After a rough spot detection with a Laplacian-of-Gaussian blob detector, subpixel localization was performed by fitting a multidimensional Gaussian using the Levenberg-Marquardt algorithm. The code for handling the microscopy data and analysis is available at: https://bitbucket.org/davidhoerl/sted-oligofish-analysis.

Chromatin environment of single FISH spots. To determine the relative chromatin compaction at the FISH spot, a maximum z-projection of the FISH stack was overlaid onto the single SiR-DNA plane (scaled with bilinear interpolation to match pixel sizes). In the resulting images, the spot position and nuclear outlines were annotated by hand. To reduce out-of-focus signal, a rolling-ball (radius=50px) background subtraction was performed on the SiR channel. For each image, the quantile of the SiR intensity at the FISH spot location with respect to all pixels in the nuclear annotation (smoothed with a Gaussian blur with sigma=1px) was determined. The results were visualized as boxplots and statistical significance of differences between inactive and active loci was assessed via a two-sided Wilcoxon rank sum test.

Distance distributions of reconstituted chromatin. To measure the end-to-end distances of ATTO594 and ATTO647N end-labelled reconstituted chromatin (25x601 system), we acquired two-channel STED images of large FOVs (2D depletion pattern, 20nm pixel size). Probe endpoints in both color channels were localized by performing Laplacian-of-Gaussian blob detection followed by subpixel refinement by fitting a Gaussian function to the detected blobs. Localized spots with a peak brightness above a manually determined threshold were discarded to ignore aggregates. Furthermore, spots having a nearest neighbor closer than 250nm in the same channel were discarded for the same reason. A matching of point pairs from both channels with minimal overall distance was determined by solving a linear assignment problem. Distances above the maximum plausible length of 250nm (corresponding to stretched beads-on-a-string) were set to a large constant in the distance matrix to exclude them from further analysis. All matches with plausible lengths from the assignment result were used to compile the final distance measurements. The result is shown in Fig. S3 *C.*

Coarse-grained modeling

Elastic energies. Elastic interactions are modelled by harmonic potentials. The strength constants of the interactions are named $a_{(Y)}^{(X)}$ where *X* denotes the type of interaction (*s*=stretching, *b*=bending, *t*=torsion) and *Y* the interaction partners (DNA or nucleosome). The energy for stretching (Eq. 2) is calculated by:

$$E_{stretch} = \frac{a_Y^{(s)}}{b_i^0} (b_i - b_i^0)^2, \qquad (2)$$

where b_i is the current length and b_i^0 is the equilibrium length of the segment. The bending energy is given by (Eq. 3):

$$E_{bending} = \frac{a_Y^{(b)}}{b_i^0} \theta_i^{\,2}, \qquad (3)$$

Where θ_i is calculated from $cos(\theta_i) = \hat{B}_i \cdot \hat{u}_{i+1}$ with $\hat{B}_i$ being the equilibrium direction of the next segment and $\hat{u}_{i+1}$ its actual direction. The torsional energy (Eq. 4) is computed as:

$$E_{torsion} = \frac{a_Y^{(t)}}{b_i^0} (\alpha_i + \gamma_i - \tau_i)^2, \qquad (4)$$

Where the angles α_i ,and γ_i are from the Euler-transformation $(\alpha_i, \beta_i, \gamma_i)$ from the local coordinate system from segment *i* to segment *i+1*. The angle τ_i is the intrinsic twist (14).

Internucleosomal interaction. The internucleosomal interaction is described by a shifted 12-6 Lennard-Jones (Eq. 5) potential

$$E_{internuc} = 4\varepsilon(\hat{o}_1, \hat{o}_2, \hat{r}) \left[\left(\frac{\sigma_0}{|\vec{r}| - \sigma(\hat{o}_1, \hat{o}_2, \hat{r}) + \sigma_0} \right)^{12} - \left(\frac{\sigma_0}{|\vec{r}| - \sigma(\hat{o}_1, \hat{o}_2, \hat{r}) + \sigma_0} \right)^{6} \right], (5)$$

where $\hat{o}_1$ and $\hat{o}_2$ denote the orientation of the nucleosome and $\vec{r}$ the distance between the centers of the nucleosomes. The shape of the nucleosome and the spatial dependency of the internucleosomal interaction strength is modelled by ε (Eq. 6) and σ (Eq. 7) depending of $\hat{o}_1, \hat{o}_2$ and $\hat{r}$. This is implemented by a series expansion in S-functions (15):

$\sigma(\hat{o}_1, \hat{o}_2, \hat{r}) = \sigma_0[\sigma_{000}S_{000} + \sigma_{cc2}(S_{202} + S_{022}) + \sigma_{220}S_{220} + \sigma_{222}S_{222} + \sigma_{224}S_{224}]$, (6)

and

$\varepsilon(\hat{o}_1, \hat{o}_2, \hat{r}) = \varepsilon[\varepsilon_{000}S_{000} + \varepsilon_{cc2}(S_{202} + S_{022}) + \varepsilon_{220}S_{220} + \varepsilon_{222}S_{222} + \varepsilon_{224}S_{224}]$, (7)

The expansion coefficients were chosen to match the spatial dimensions of the nucleosome and data from force spectroscopy experiments (16-18).

DNA-Nucleosome excluded volume. The volume of DNA segments is approximated by spheres. The minimal distance d between the center of DNA sphere and a spherocylinder describing the nucleosomes is computed. The excluded volume energies $E_{DNA-Nuc}$ is described as the sum of the individual excluded volume energies $E'_{DNA-Nuc}$ (Eq. 8) computed for DNA sphere and the volume of the nucleosome:

$$E'_{DNA-Nuc} = \begin{cases} 0 & if\ d \geq r_n + r_d \\ k(d - r_n - r_d)^{12} & else \end{cases}, \qquad (8)$$

with $r_n = (5.5/2)$ nm and $r_d = 1.2\ nm$.

Electrostatic energy of linker DNA. A DNA segment is modelled by a chain c of charged spheres The GROMACS unit system was used which is based on nm, ps, K, electron charge (e) and atomic mass unit (u) (19).

The electrostatic energy of two spheres with charge q_1 and q_2 and radius a separated by a center-to-center distance r can be approximated by the electrostatic part of the Derjaguin-Landau-Verwey-Overbeek theory (20,21) as (Eq. 9)

$$E_{el}(r) = \frac{1}{4\pi\varepsilon\varepsilon_0}\, q_1 q_2 \left(\frac{e^{\kappa\alpha}}{1+\kappa\alpha}\right)^2 \frac{e^{-\kappa r}}{r}, \qquad (9)$$

With κ being the inverse Debye length (Eq. 10) calculated by:

$$\kappa^2 = \frac{2e_c^2 \rho N_A}{\varepsilon\varepsilon_0 k_B T}, \qquad (10)$$

For the values listed in Table S2 κ yields $\kappa = 1.0387\ nm^{-1}$ which corresponds to a Debye length of $\lambda_D = \kappa^{-1} = 0.96\ nm$.

The charge of a DNA segment is given by $q = \nu d$, with ν being the nominal line charge density ($-2/0.34\ e_c\ nm^{-1}$) and d the length of the DNA represented by the sphere. The line charge density ν of the DNA must be adapted to the effective charge density (Eq. 11) $\nu *$

$$\nu *= \nu \chi_{CR} \chi_{PBS}, \qquad (11)$$

Where χ_{CR} is the charge adaptation factor and χ_{PBS} accounts for the geometry of subsequent overlapping beads and for deviations due to using an approximation instead of the exact Poisson-Boltzmann (PB) equation (22). Here, we use for χ_{CR} a value of 0.42 as derived in (22). The adaptation factor χ_{PBS} was determined by relating this potential to previous description as cylindrical segments (22).

Preparation and simulation. For the preparation of the simulation data we first selected an appropriate human genome dataset (MNAse-seq of K-562 cells from the ENCODE project ENCSR000CXQ (23,24)) in BigWig format (ENCFF000VNN). Next, we converted this file into the WIG-Format applying the BigWig2Wig-tool and finally in a BED format by a simple awk-script. Reads from chromosome 11 were extracted applying another simple UNIX-awk-script. In order to avoid false positive nucleosome positions blacklisted regions were filtered out (https://www.encodeproject.org/files/ENCFF001TDO/). Best nucleosome positions were determined with NucPosSimulator (25) generating a BED file containing the nucleosome positions and the occupancy, i.e. the number of read centers counted per base pair, smoothed with a Gaussian kernel and normalized. For identifying the least probable nucleosome the mean occupancy values of the 147 bp regions classified as nucleosomes by NucPosSimulator were determined and sorted. After removing the chosen number of nucleosomes with the smallest values, we generated a nucleosome chain with liker lengths as in the region and performed computer simulations (26). In order to incorporate effects of surrounding chromatin nucleosomes 20 kb were included at both sites of both investigated regions. The simulations were carried out on the linux cluster in Stralsund and the North German Supercomputing Alliance (HLRN) in Berlin.

Calculation of nucleosome repeat length. The nucleosome repeat length (NRL) of whole chromosome 11 was determined analyzing the chr11 BED-file as described in the previous section. In a preparatory step nucleosome positions for the whole chromosome 11 were determined applying NucPosSimulator. From resulting sorted paired end nucleosome reads the repeat length between adjacent nucleosomes was calculated by subtracting the last base pair to the first base pair of the following nucleosome read. The average NRL a sliding window was calculated for a window size of 30000 bp. From this dataset windows with less than 3 nucleosomes e.g. in the centromere were removed applying filter-function from R package "dplyr" (filter(dataset(`#Nucs`!=3))). The developed script (plotNRL.R) is published in a codeocean.com capsule (https://codeocean.com/capsule/8421512/tree/v2).

Analysis of correlation of simulated configurations

Maximum value of internucleosomal interaction energy 4 kT: We performed 10 x 10^6 Monte Carlo steps per replica after simulated annealing utilizing about 10^3 core hours for each system on the HLRN supercomputer. For the analysis of autocorrelation, we performed a test simulation where we saved every 25 Monte Carlo steps. We computed the autocorrelation of the end-to-end distances and the energies and concluded that the correlation length between configurations is about 800 steps. To be on the safe side we considered configurations uncorrelated after 10x10^3 steps. For equilibration we excluded the first about 10^5 steps after simulated annealing from the analysis. The exact values are given in table S3 in supplemental material. Therefore, we generated nearly 1000 uncorrelated configurations in the lowest temperature we used to calculate the distance distribution plots.

Maximum value of internucleosomal interaction energy 6 kT: We performed 90 x 10^6 steps per replica after simulated annealing utilizing about 30x10^3 core hours for each system on the HLRN supercomputer. For the analysis of autocorrelation, we performed a test simulation where we saved every 75 Monte Carlo steps. We computed autocorrelation the end-to-end distances and the energies, and we concluded that the correlation length between configurations is about 2.5 x10^3 steps. To be on

the safe side we considered configurations uncorrelated after $20x10^3$ steps. We excluded the first half, $60x10^6$ steps, from the analysis for equilibration. Therefore, we generated 2000 uncorrelated configurations in the lowest temperature we used to calculate the distance distribution plots.

The 25x601 system contains only 25 nucleosomes which is only 10 % of the 251 nucleosomes of the other simulations. We estimated, that $2.5x10^3$ steps, a quarter of the steps used for the active region, are more than enough for considering two configurations as uncorrelated.

Statistical analysis

Statistics and reproducibility. No statistical method was used to predetermine sample size. Investigators were not blinded during the experiments and when assessing the outcome. For each experiment, data were collected from at least three independent biological replicates.

Plots in Fig. 2-5 and Fig. S4 and S5 were generated using ggplot2 in R Studio (ver. 1.3.1056) (27). Significance levels were always tested by a non-parametric two-sided Wilcoxon rank sum test and a Bonferroni-Holm correction was used to avoid errors through multiple testing when applicable. Data in Fig. 2-3 and Fig. S5 are represented as boxplots where the middle line indicates the median, the lower and upper hinges correspond to the 25% and 75% quartiles, the upper whisker extends to the largest value no further than 1.5 x IQR (inter-quartile range) from the hinge and the lower whisker extends to the smallest value from the hinge at most 1.5 x IQR. The data acquisition, image processing and analysis was done in an unbiased way by automation.

SUPPORTING TABLES

TABLE S1 The genomic coordinates and sequences of the used oligonucleotide probes can be found in the separate file named *Oligonucleotide_probes.xlsx*

TABLE S2 Simulation parameters and constants

e_c	1.602 ´ 10^{-19} C	Electric charge unit
v	–2/0.34 e_c nm^{-1}	Line charge density of DNA
ρ	0.1 ´ 10^{24} mol nm^{-3}	Molarity of the monovalent solution
N_A	6.022 ´ 10^{23} mol^{-1}	Avogadro constant
ε	80	Value for the dielectric value in the solution
ε_0	$(4\pi f)^{-1}$	Dielectric constant
f	138.935 kJ nm mol^{-1} e_c^{-2}	Electric conversion factor
k_B	8.314513 ´ 10^{-3} kJ mol^{-1} K^{-1}	Boltzmann constant
a	1.2 nm	Radius of the DNA model sphere
T	295 K	Temperature of the solution
	10 nm	maximum DNA segment length
	5.5 nm	nucleosome height
	11 nm	nucleosome diameter
	4 k_BT and 6 k_BT (inactive)	ε for $E_{internuc}$
	5.5 nm	σ for $E_{internuc}$
	665	$a_{DNA}^{(s)}$
	665	$a_{NUC}^{(s)}$
	120.44	$a_{DNA}^{(b)}$
	120.44	$a_{NUC}^{(b)}$
	219.25	$a_{DNA}^{(t)}$
	782.85	$a_{NUC}^{(t)}$
	1.2 kJ mol^{-1}	Lennard jones ε for DNA
	2.0 kJ mol^{-1}	Lennard jones σ for DNA
	$S000$ = 1.6957	interaction potential nucleosome s-functions
	$Scc2$ = –0.7641	
	$S220$ = –0.1480	
	$S222$ = –0.2582	
	$S224$ = 0.5112	
	$E000$ = 2.7206	
	$Ecc2$ = 6.0995	
	$E220$ = 3.3826	
	$E222$ = 7.1036	
	$E224$ = 3.2870	

TABLE S3 Overview over simulation steps in Monte Carlo simulations: All simulations steps (MCS = Monte Carlo steps), steps in simulated annealing (SA), steps in replica exchange (RE), The number of steps for equilibration and where the analysis starts, the estimated number of uncorrelated configurations used in the analysis, the maximal temperature in RE and the number of replicas.

	Total MCS [10^6]	MCS SA [10^6]	MCS RE [10^6]	Analysis start in MCS	Estimated #uncorrelated configurations	Maximal temperature [K]	#Replicas
Active Full	20	10	10	10120000	992	370	16
Active -1	20	10	10	10120000	992	370	16
Active -2	20	10	10	10120000	992	370	16
Active -3	20	10	10	10120000	992	370	16
Active -4	20	10	10	10120000	992	370	16
Active -5	20	10	10	10120000	992	370	16
Active Gafney	30	20	10	20040000	999	370	16
Active -5 Gafney	30	20	10	20040000	999	370	16
Active 45% H1	50	10	40	40020000	2000	450	32
Active H1	71	20	51	31040000	2000	450	32
Active -2 H1	59	20	39	39940000	2000	450	32
Inactive	20	10	10	10200000	982	370	16
Inactive H1	20	10	10	10200000	982	370	16
Inactive 6 KbT	100	10	90	72040000	2000	590	60
Inactive 6 kbT H1 70 %	100	10	90	60060000	2000	590	60
Inactive 6 KbT H1	100	10	90	60060000	2000	590	60
25x601 4KbT	15	5	10	10002500	2001	445	16
25x601 6KbT	15	5	10	10002500	2001	445	16

TABLE S4 Statistical data for 2D STED datasets

p-values for Active AB – BC – CD – DE

	**Active AB**	**Active BC**	**Active CD**
**Active BC**	0.03220	-	-
**Active CD**	0.02706	0.80123	-
**Active DE**	0.18970	0.00053	0.00053

p-values for Inactive AB – BC – CD – DE

	**Inactive AB**	**Inactive BC**	**Inactive CD**
**Inactive BC**	$3.3 * 10^{-7}$	-	-
**Inactive CD**	$5.8 * 10^{-9}$	0.451	-
**Inactive DE**	$1.2 * 10^{-12}$	0.071	0.451

p-value for all active vs. all inactive

	**Active**
**Inactive**	$<2 * 10^{-16}$

Test: Wilcoxon rank sum test with continuity correction. Correction method: Bonferroni holm correction for multiple testing. Software: R studio, Significant: $p < 0.05$.

TABLE S5 Statistical data for 3D STED datasets

p-values for Active AB – BC – CD – DE

	**Active AB**	**Active BC**	**Active CD**
**Active BC**	0.895	-	-
**Active CD**	0.138	0.076	-
**Active DE**	1.000	1.000	0.525

p-values for Inactive AB – BC – CD – DE

	**Inactive AB**	**Inactive BC**	**Inactive CD**
**Inactive BC**	$8 * 10^{-4}$	-	-
**Inactive CD**	$8.3 * 10^{-5}$	1.000	-
**Inactive DE**	$9.4 * 10^{-5}$	1.000	1.000

p-value for all active vs. all inactive

	**Active**
**Inactive**	$<2 * 10^{-16}$

Test: Wilcoxon rank sum test with continuity correction. Correction method: Bonferroni holm correction for multiple testing. Software: R studio. Significant: $p < 0.05$.

SUPPORTING FIGURES

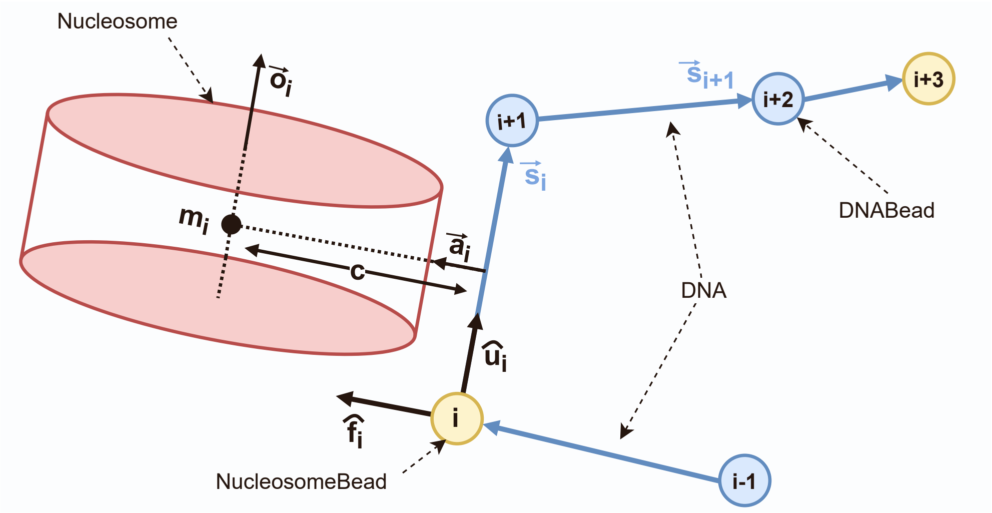

FIGURE S1 Model of a nucleosome chain. *i* represents the position of the bead in the chain, yellow circles indicate nucleosome bead positions, and blue circles indicate DNA bead positions. The nucleosome is represented by a red cylinder. The segment vector $\vec{s_i}$ points from one bead to the next bead. A local coordination system $(\hat{u}_i, \hat{v}_i, \hat{f}_i)$ (not shown) describes the orientation of a bead. Vector $\vec{a_i}$ describes the direction from the center of the segment to the nucleosome center m_i, c is its length, and vector $\vec{o_i}$ describes the orientation of the nucleosome. Vector $\vec{a_i}$ is defined by two rotations of vector $\hat{v}_i$ (i) around $\vec{u_i}$ by the angle ε (not shown), (ii) around vector $\hat{f}_i$ by the angle ϕ (not shown).

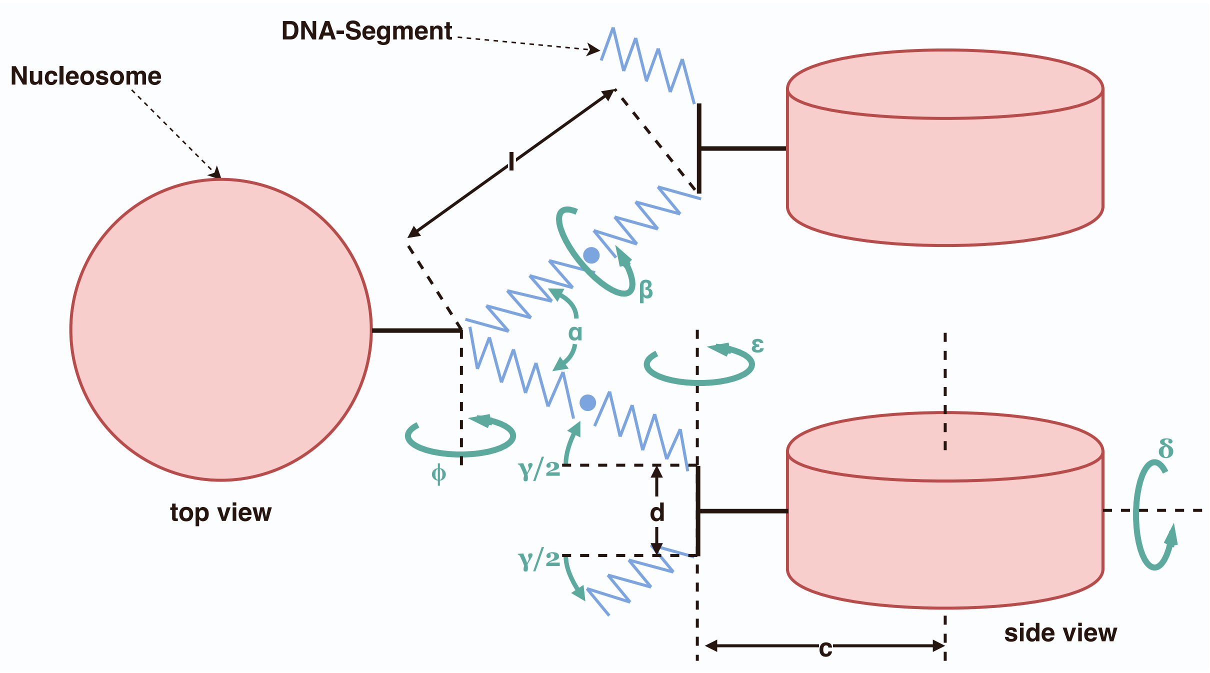

FIGURE S2 The relative orientation of the nucleosome is described by the angles $\alpha, \delta, \varepsilon, \gamma, \phi$ (modified from (28)). β is the torsional orientation of subsequent nucleosomes. l is the length of the DNA modeling the linker DNA, d the distance between the entry and the exit point of the linker DNA at the nucleosome, c the distance between the center of the nucleosome segment and the center of the oblate sphero-cylinder modeling of the nucleosome.

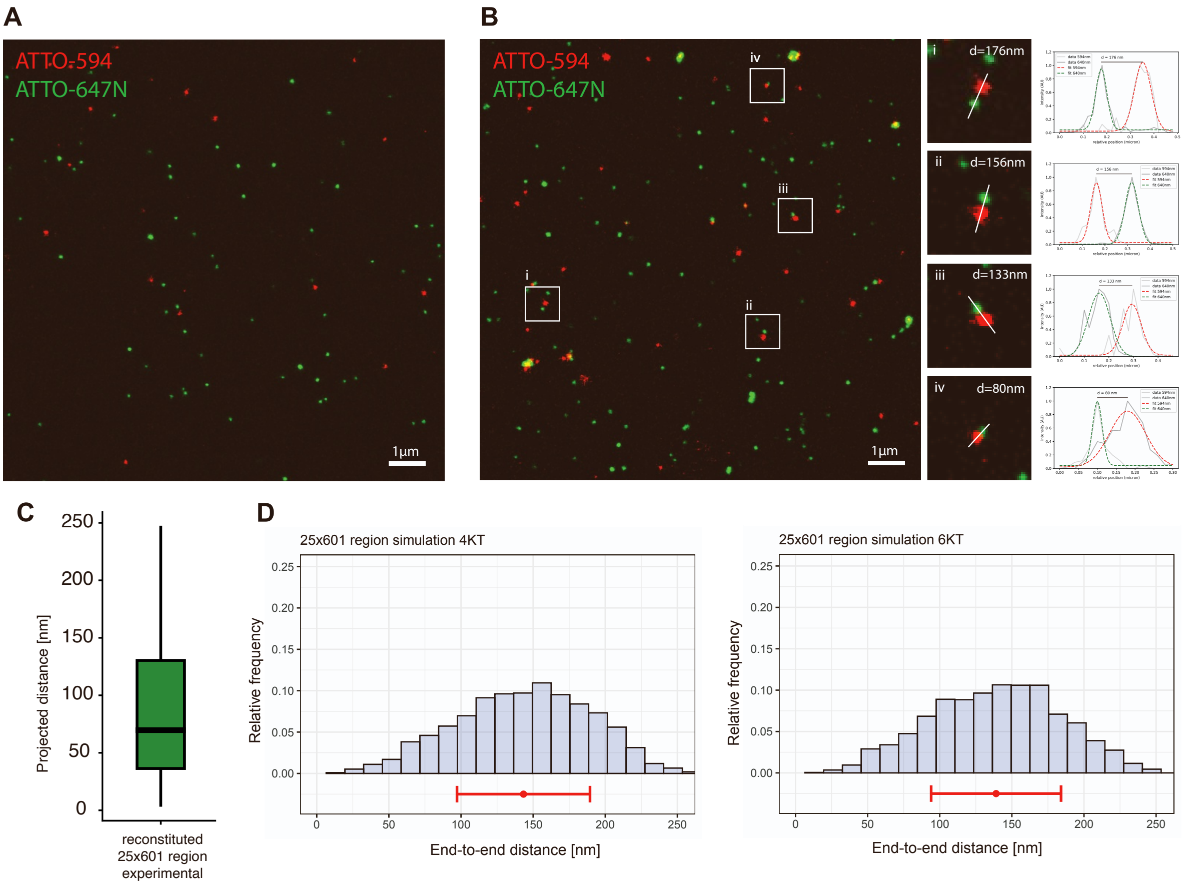

FIGURE S3 Measurement of microscope resolution with reconstituted chromatin. (*A*) Representative dual-channel STED image of ATTO-594 and ATTO-647N end-labelled DNA fragments (25x601) as described in Supporting Materials and Methods (*B*) Representative STED image of chromatin reconstituted from end-labelled 25x601 fragments. Right: magnified view of 1x1 micron around regions i-iv and line profiles. Distances were determined by fitting Gaussian functions to the line profiles in both channels and measuring the difference of the peak locations. (*C*) Box plot of the microscopically measured distances of n=230 reconstituted 25x601 fragments, median=70 nm (*D*) Distance distributions from simulations of the reconstituted 25x601 region as described in the Supporting Materials and Methods and the main text, 4KT (left) and 6KT (right). Mean value and standard deviation are shown below in red.

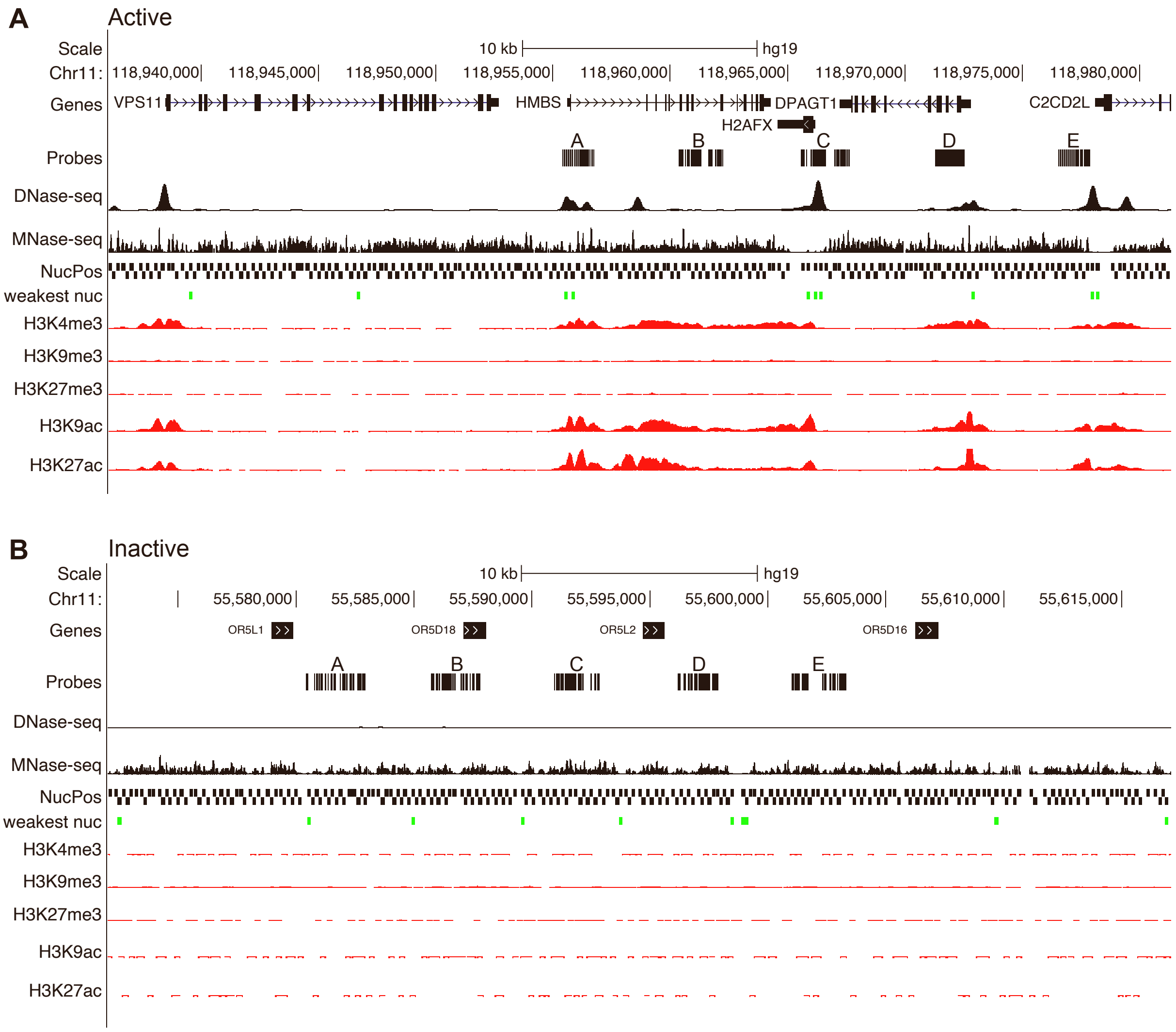

FIGURE S4 Active (*A*) and inactive (*B*) genomic regions with genes, probe sets (A-E), DNase-seq (GSM816655), MNase-seq (GSM920557), nucleosome positions from NucPosSimulator, the weakest nucleosomes calculated by NucPosSimulator, H3K4me3 (GSM733680), H3K9me3 (GSM733776), H3K27me3 (GSM733658), H3K9ac (GSM733778), H3K27ac (GSM733656). Tracks show that inactive region has almost no histone modifications while the active region contains active marks like H3K4me3, H3K9ac and H3K27ac. Notably, most of the weakest nucleosomes for the active region are located at DNase I hypersensitive sites. Plot was generated with the UCSC genome browser (29) (http://genome.ucsc.edu).

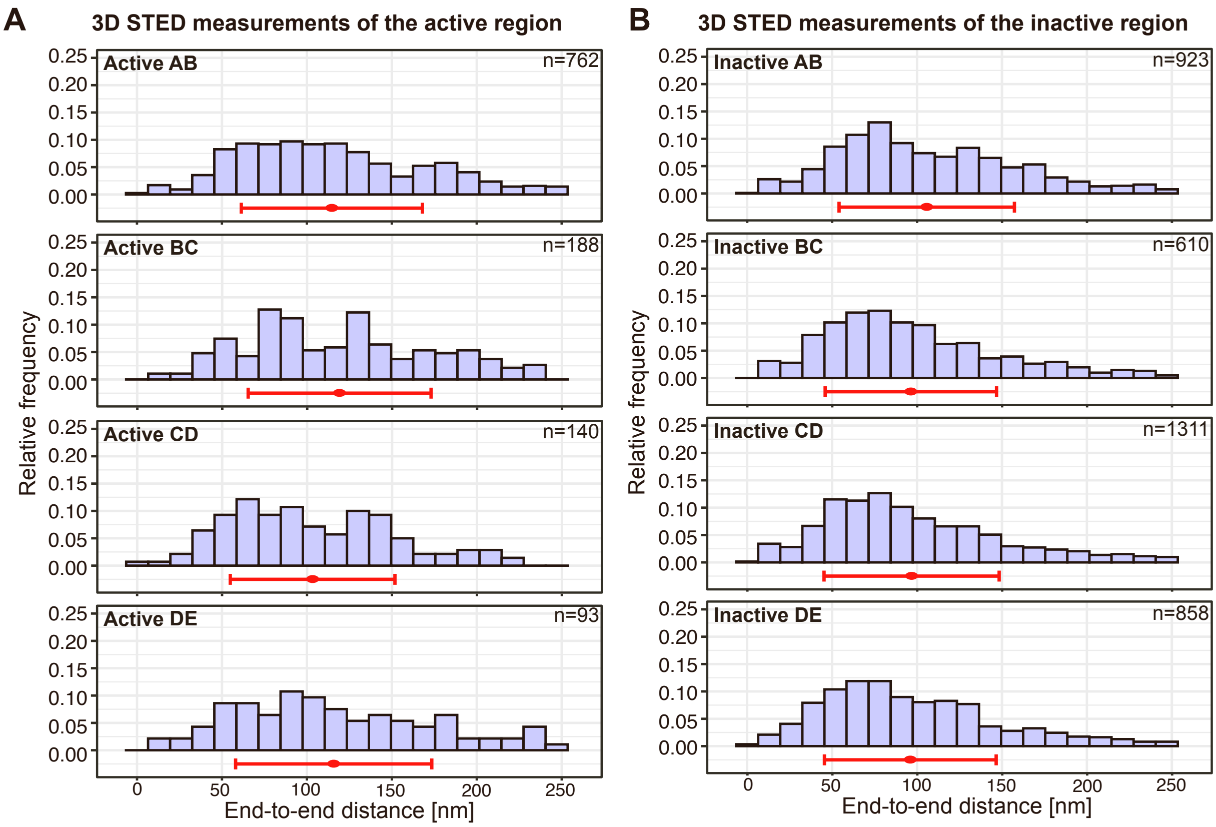

FIGURE S5 Distance histograms from 3D STED measurements for all four intervals (AB, BC, CD, DE) in active (*A*) and inactive (*B*). The mean for each histogram is indicated by the red dot and the standard deviation by the red line. N-numbers can be found next to the respective histogram (for statistical data, see Table S4).

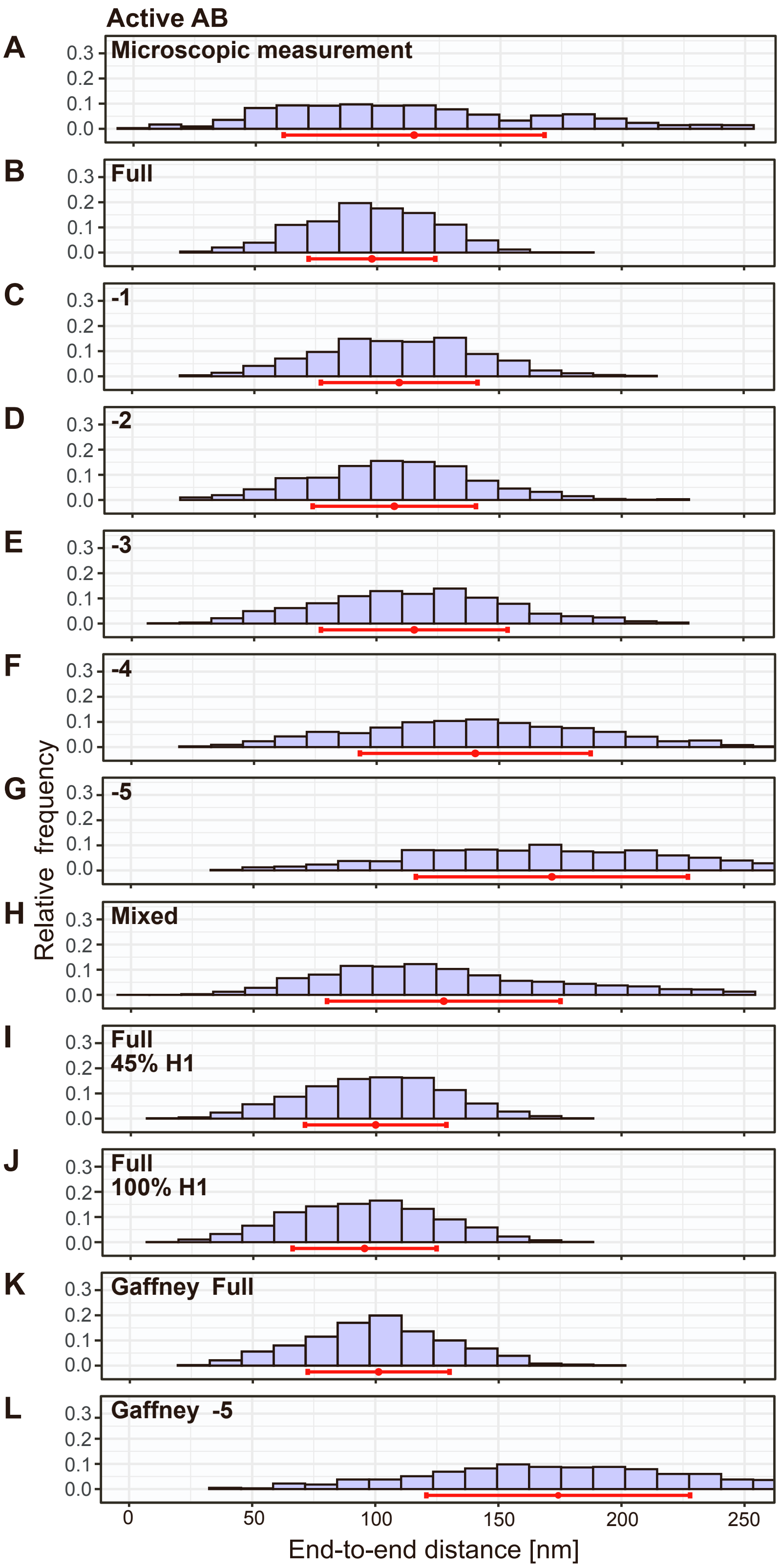

FIGURE S6 Distance distributions from simulations of the of the active locus. (*A-H*) same data as in Fig. 4 of the manuscript. As control the full locus was simulated in the presence of (*I*) 45% and (*J*) 100% H1 binding. Here, no effect was observed. (*K-L*) As second control simulations were performed with nucleosome positions derived from a lymphoblastoid cell lines (30). (*K*) all nucleosomes, (*L*) 5 nucleosomes with lowest occupancy replaced by naked DNA. Again, no effect was observed.

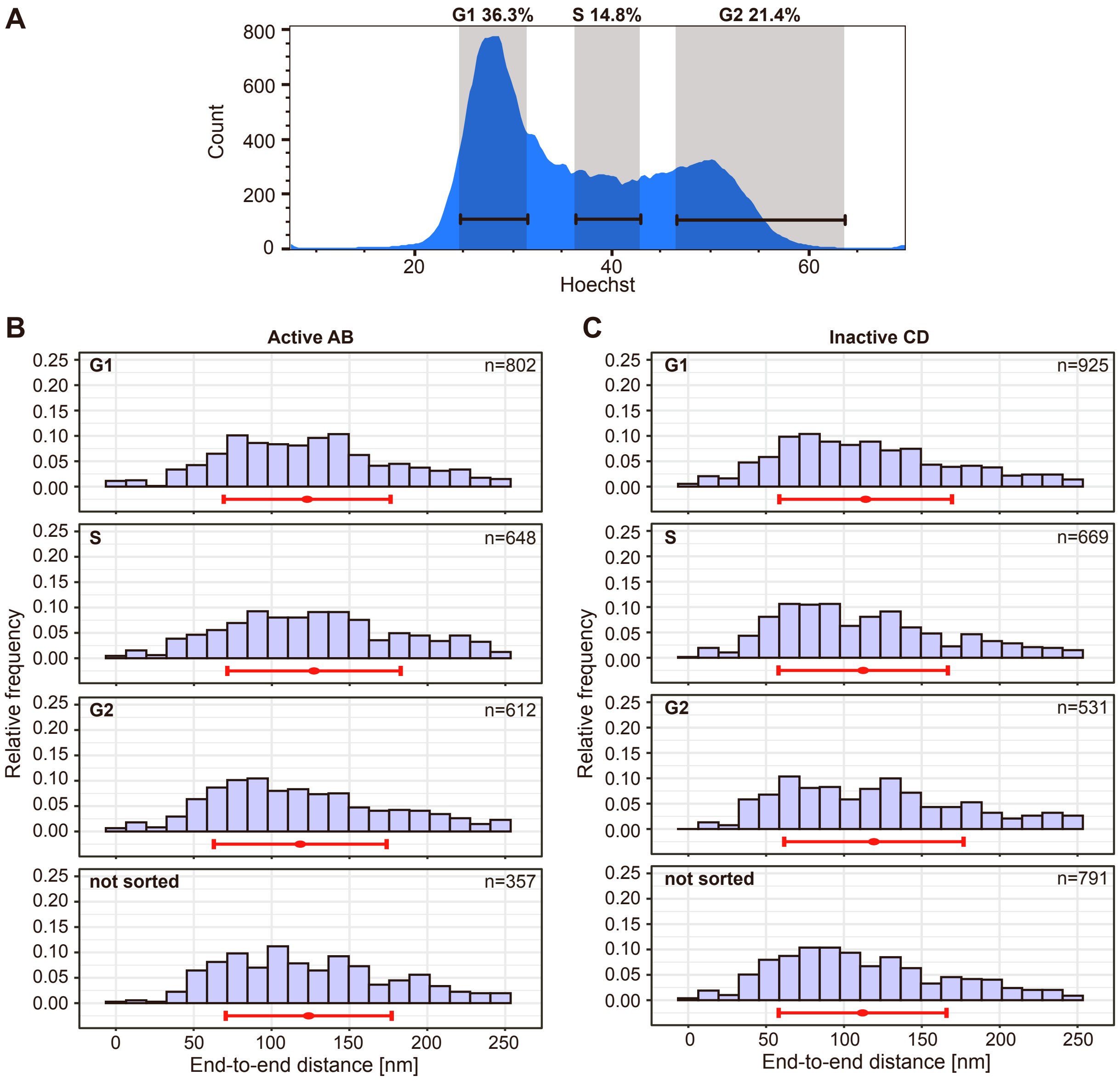

FIGURE S7 Chromatin compaction in cells of different cell cycle stages. Cells were flow-sorted according to their DNA content to investigate a possible influence of the cell cycle on the microscopically measured distance distribution of FISH spots 5 kb apart (*A*) Representative sorting profile of Hoechst-stained K562 cells, gate settings and percentage of cells in each cell cycle phase. (*B*) Distance distribution in active region AB for G1, S, G2 phase and unsorted control cells (6 replicates). (*C*) Histograms for the inactive region CD according to (*B*) using data from 4 replicates. Mean and standard deviation are shown in red under each histogram, n number of cells analyzed. Extended DNA configurations are also found in G1 phase, arguing against an effect of replication on the broadening of the histogram.

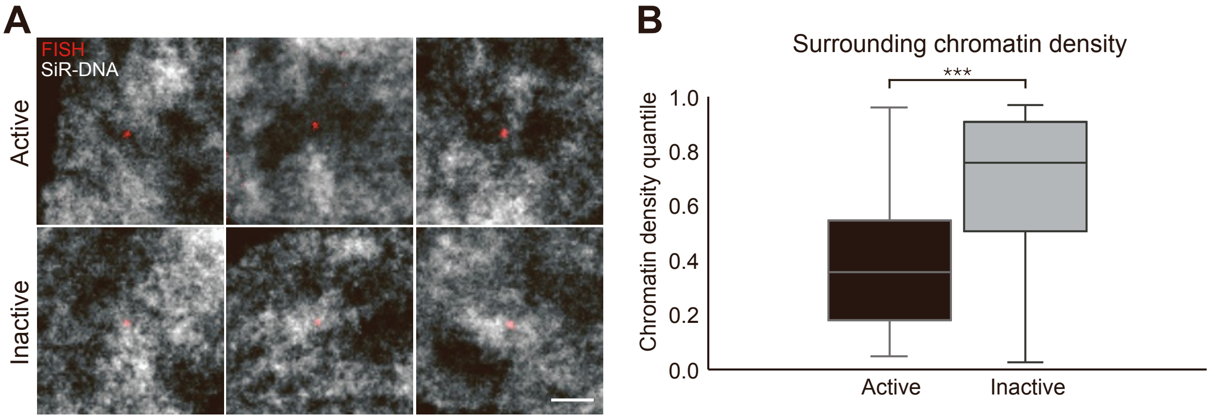

FIGURE S8 Chromatin environment of the active and inactive region. (*A*) Representative images for the active (upper row) and inactive (lower row) region labeled with one FISH probe set (red) and chromatin labeled with SiR-DNA (gray). Scale bar = 1 µm. (*B*) Chromatin density quantile for active (black, n=43) and inactive (gray, n=38) differ significantly (two-sided Wilcoxon rank sum test, $p = 9.890 * 10^{-6}$). Inactive region is embedded in higher density chromatin, while active chromatin is surrounded by lower density chromatin.

SUPPORTING REFERENCES

1. Wurm, C. A., D. Neumann, R. Schmidt, A. Egner, and S. Jakobs. 2010. Sample preparation for STED microscopy. In Live cell imaging. Springer, pp. 185-199.
2. Meuleman, W., A. Muratov, E. Rynes, J. Halow, K. Lee, D. Bates, M. Diegel, D. Dunn, F. Neri, A. Teodosiadis, A. Reynolds, E. Haugen, J. Nelson, A. Johnson, M. Frerker, M. Buckley, R. Sandstrom, J. Vierstra, R. Kaul, and J. Stamatoyannopoulos. 2020. Index and biological spectrum of human DNase I hypersensitive sites. *Nature*. 584(7820):244-251, doi: 10.1038/s41586-020-2559-3.
3. John, S., P. J. Sabo, R. E. Thurman, M. H. Sung, S. C. Biddie, T. A. Johnson, G. L. Hager, and J. A. Stamatoyannopoulos. 2011. Chromatin accessibility pre-determines glucocorticoid receptor binding patterns. *Nat Genet*. 43(3):264-268, doi: 10.1038/ng.759.
4. Bintu, B., L. J. Mateo, J. H. Su, N. A. Sinnott-Armstrong, M. Parker, S. Kinrot, K. Yamaya, A. N. Boettiger, and X. Zhuang. 2018. Super-resolution chromatin tracing reveals domains and cooperative interactions in single cells. *Science*. 362(6413):eaau1783, doi: 10.1126/science.aau1783.
5. Boettiger, A. N., B. Bintu, J. R. Moffitt, S. Wang, B. J. Beliveau, G. Fudenberg, M. Imakaev, L. A. Mirny, C. T. Wu, and X. Zhuang. 2016. Super-resolution imaging reveals distinct chromatin folding for different epigenetic states. *Nature*. 529(7586):418-422, doi: 10.1038/nature16496.
6. Markaki, Y., D. Smeets, S. Fiedler, V. J. Schmid, L. Schermelleh, T. Cremer, and M. Cremer. 2012. The potential of 3D-FISH and super-resolution structured illumination microscopy for studies of 3D nuclear architecture: 3D structured illumination microscopy of defined chromosomal structures visualized by 3D (immuno)-FISH opens new perspectives for studies of nuclear architecture. *Bioessays*. 34(5):412-426, doi: 10.1002/bies.201100176.
7. Solovei, I., A. Cavallo, L. Schermelleh, F. Jaunin, C. Scasselati, D. Cmarko, C. Cremer, S. Fakan, and T. Cremer. 2002. Spatial preservation of nuclear chromatin architecture during three-dimensional fluorescence in situ hybridization (3D-FISH). *Exp Cell Res*. 276(1):10-23, doi: 10.1006/excr.2002.5513.
8. Branco, M. R., and A. Pombo. 2006. Intermingling of chromosome territories in interphase suggests role in translocations and transcription-dependent associations. *PLoS Biol*. 4(5):e138, doi: 10.1371/journal.pbio.0040138.
9. Göttfert, F., C. A. Wurm, V. Mueller, S. Berning, V. C. Cordes, A. Honigmann, and S. W. Hell. 2013. Coaligned dual-channel STED nanoscopy and molecular diffusion analysis at 20 nm resolution. *Biophysical journal*. 105(1):L01-L03.
10. Esa, A., P. Edelmann, G. Kreth, L. Trakhtenbrot, N. Amariglio, G. Rechavi, M. Hausmann, and C. Cremer. 2000. Three-dimensional spectral precision distance microscopy of chromatin nanostructures after triple-colour DNA labelling: a study of the BCR region on chromosome 22 and the Philadelphia chromosome. *J Microsc*. 199(Pt 2):96-105, doi: 10.1046/j.1365-2818.2000.00707.x.
11. Lieleg, C., P. Ketterer, J. Nuebler, J. Ludwigsen, U. Gerland, H. Dietz, F. Mueller-Planitz, and P. Korber. 2015. Nucleosome spacing generated by ISWI and CHD1 remodelers is constant regardless of nucleosome density. *Mol Cell Biol*. 35(9):1588-1605, doi: 10.1128/MCB.01070-14.
12. Oberbeckmann, E., N. Krietenstein, V. Niebauer, Y. Wang, K. Schall, M. Moldt, T. Straub, R. Rohs, K. P. Hopfner, P. Korber, and S. Eustermann. 2021. Genome

information processing by the INO80 chromatin remodeler positions nucleosomes. *Nat Commun*. 12(1):3231, doi: 10.1038/s41467-021-23016-z.
13. Van Rossum, G., Drake, F. L. 2009. Python 3 Reference Manual. *Scotts Valley, CA: Create Space*.
14. Klenin, K., H. Merlitz, and J. Langowski. 1998. A Brownian dynamics program for the simulation of linear and circular DNA and other wormlike chain polyelectrolytes. *Biophys J*. 74(2 Pt 1):780-788, doi: 10.1016/S0006-3495(98)74003-2.
15. Zewdie, H. 1998. Computer simulation studies of liquid crystals: A new Corner potential for cylindrically symmetric particles. *The Journal of chemical physics*. 108(5):2117-2133.
16. Stehr, R., N. Kepper, K. Rippe, and G. Wedemann. 2008. The effect of internucleosomal interaction on folding of the chromatin fiber. *Biophys J*. 95(8):3677-3691, doi: 10.1529/biophysj.107.120543.
17. Stehr, R., R. Schopflin, R. Ettig, N. Kepper, K. Rippe, and G. Wedemann. 2010. Exploring the conformational space of chromatin fibers and their stability by numerical dynamic phase diagrams. *Biophys J*. 98(6):1028-1037, doi: 10.1016/j.bpj.2009.11.040.
18. Kepper, N., R. Ettig, R. Stehr, S. Marnach, G. Wedemann, and K. Rippe. 2011. Force spectroscopy of chromatin fibers: extracting energetics and structural information from Monte Carlo simulations. *Biopolymers*. 95(7):435-447, doi: 10.1002/bip.21598.
19. Hess, B., C. Kutzner, D. van der Spoel, and E. Lindahl. 2008. GROMACS 4: Algorithms for Highly Efficient, Load-Balanced, and Scalable Molecular Simulation. *J Chem Theory Comput*. 4(3):435-447, doi: 10.1021/ct700301q.
20. Levin, Y. 2002. Electrostatic correlations: from plasma to biology. *Reports on progress in physics*. 65(11):1577.
21. Walker, D. A., B. Kowalczyk, M. O. de la Cruz, and B. A. Grzybowski. 2011. Electrostatics at the nanoscale. *Nanoscale*. 3(4):1316-1344, doi: 10.1039/c0nr00698j.
22. Maffeo, C., R. Schopflin, H. Brutzer, R. Stehr, A. Aksimentiev, G. Wedemann, and R. Seidel. 2010. DNA-DNA interactions in tight supercoils are described by a small effective charge density. *Phys Rev Lett*. 105(15):158101, doi: 10.1103/PhysRevLett.105.158101.
23. Davis, C. A., B. C. Hitz, C. A. Sloan, E. T. Chan, J. M. Davidson, I. Gabdank, J. A. Hilton, K. Jain, U. K. Baymuradov, A. K. Narayanan, K. C. Onate, K. Graham, S. R. Miyasato, T. R. Dreszer, J. S. Strattan, O. Jolanki, F. Y. Tanaka, and J. M. Cherry. 2018. The Encyclopedia of DNA elements (ENCODE): data portal update. *Nucleic Acids Res*. 46(D1):D794-D801, doi: 10.1093/nar/gkx1081.
24. Consortium, E. P., J. E. Moore, M. J. Purcaro, H. E. Pratt, C. B. Epstein, N. Shoresh, J. Adrian, T. Kawli, C. A. Davis, A. Dobin, R. Kaul, J. Halow, E. L. Van Nostrand, P. Freese, D. U. Gorkin, Y. Shen, Y. He, M. Mackiewicz, F. Pauli-Behn, B. A. Williams, A. Mortazavi, C. A. Keller, X. O. Zhang, S. I. Elhajjajy, J. Huey, D. E. Dickel, V. Snetkova, X. Wei, X. Wang, J. C. Rivera-Mulia, J. Rozowsky, J. Zhang, S. B. Chhetri, J. Zhang, A. Victorsen, K. P. White, A. Visel, G. W. Yeo, C. B. Burge, E. Lecuyer, D. M. Gilbert, J. Dekker, J. Rinn, E. M. Mendenhall, J. R. Ecker, M. Kellis, R. J. Klein, W. S. Noble, A. Kundaje, R. Guigo, P. J. Farnham, J. M. Cherry, R. M. Myers, B. Ren, B. R. Graveley, M. B. Gerstein, L. A. Pennacchio, M. P. Snyder, B. E. Bernstein, B. Wold, R. C. Hardison, T. R. Gingeras, J. A. Stamatoyannopoulos, and Z. Weng. 2020. Expanded encyclopaedias of DNA elements in the human and mouse genomes. *Nature*. 583(7818):699-710, doi: 10.1038/s41586-020-2493-4.

25. Schopflin, R., V. B. Teif, O. Muller, C. Weinberg, K. Rippe, and G. Wedemann. 2013. Modeling nucleosome position distributions from experimental nucleosome positioning maps. *Bioinformatics*. 29(19):2380-2386, doi: 10.1093/bioinformatics/btt404.
26. Mörl, M.-C., T. Zülske, R. Schöpflin, and G. Wedemann. 2019. Data formats for modelling the spatial structure of chromatin based on experimental positions of nucleosomes. *AIMS Biophysics*. 6(3):83.
27. RStudioTeam. 2020. RStudio: Integrated Development for R. . *RStudio, PBC, Boston, MA*.
28. Rippe, K., R. Stehr, and G. Wedemann. 2012. Monte Carlo Simulations of Nucleosome Chains to Identify Factors that Control DNA Compaction and Access. *Rsc Biomol Sci*.(24):198-235, doi: 10.1039/9781849735056-00198.
29. Kent, W. J., C. W. Sugnet, T. S. Furey, K. M. Roskin, T. H. Pringle, A. M. Zahler, and D. Haussler. 2002. The human genome browser at UCSC. *Genome Research*. 12(6):996-1006, doi: 10.1101/gr.229102.
30. Gaffney, D. J., G. McVicker, A. A. Pai, Y. N. Fondufe-Mittendorf, N. Lewellen, K. Michelini, J. Widom, Y. Gilad, and J. K. Pritchard. 2012. Controls of nucleosome positioning in the human genome. *PLoS Genet*. 8(11):e1003036, doi: 10.1371/journal.pgen.1003036.
