# Supplementary material for: Differences in nanoscale organization of regulatory active and inactive human chromatin
Source: Biophys J. 2022 Feb 10;121(6):977–90. doi: 10.1016/j.bpj.2022.02.009 (PMC8943813; doi:10.1016/j.bpj.2022.02.009)
Supplement: Document S2. Article plus supporting material [file mmc3.pdf]

Biophysical Journal
Article

Biophysical Society

Differences in nanoscale organization of regulatory active and inactive human chromatin

Katharina Brandstetter,[1,7] Tilo Zülske,[2,7] Tobias Ragoczy,[3] David Hörl,[1] Miguel Guirao-Ortiz,[1] Clemens Steinek,[1] Toby Barnes,[4] Gabriela Stumberger,[1] Jonathan Schwach,[1] Eric Haugen,[3] Eric Rynes,[3] Philipp Korber,[4] John A. Stamatoyannopoulos,[3,5,6] Heinrich Leonhardt,[1] Gero Wedemann,[2,*] and Hartmann Harz[1,*]

[1]Human Biology & BioImaging, Faculty of Biology, Ludwig-Maximilians-Universität München, Munich, Germany; [2]Competence Center Bioinformatics, Institute for Applied Computer Science, Hochschule Stralsund, Stralsund, Germany; [3]Altius Institute for Biomedical Sciences, Seattle, Washington; [4]Biomedical Center (BMC), Molecular Biology, Faculty of Medicine, Ludwig-Maximilians-Universität München, Martinsried, Germany; [5]Department of Genome Sciences, University of Washington, Seattle, Washington; and [6]Department of Medicine, Division of Oncology, University of Washington, Seattle, Washington

ABSTRACT Methodological advances in conformation capture techniques have fundamentally changed our understanding of chromatin architecture. However, the nanoscale organization of chromatin and its cell-to-cell variance are less studied. Analyzing genome-wide data from 733 human cell and tissue samples, we identified 2 prototypical regions that exhibit high or absent hypersensitivity to deoxyribonuclease I, respectively. These regulatory active or inactive regions were examined in the lymphoblast cell line K562 by using high-throughput super-resolution microscopy. In both regions, we systematically measured the physical distance of 2 fluorescence in situ hybridization spots spaced by only 5 kb of DNA. Unexpectedly, the resulting distance distributions range from very compact to almost elongated configurations of more than 200-nm length for both the active and inactive regions. Monte Carlo simulations of a coarse-grained model of these chromatin regions based on published data of nucleosome occupancy in K562 cells were performed to understand the underlying mechanisms. There was no parameter set for the simulation model that can explain the microscopically measured distance distributions. Obviously, the chromatin state given by the strength of internucleosomal interaction, nucleosome occupancy, or amount of histone H1 differs from cell to cell, which results in the observed broad distance distributions. This large variability was not expected, especially in inactive regions. The results for the mechanisms for different distance distributions on this scale are important for understanding the contacts that mediate gene regulation. Microscopic measurements show that the inactive region investigated here is expected to be embedded in a more compact chromatin environment. The simulation results of this region require an increase in the strength of internucleosomal interactions. It may be speculated that the higher density of chromatin is caused by the increased internucleosomal interaction strength.

SIGNIFICANCE Conformation capture techniques are limited to measuring contact probability. Here, we focused on a complementary aspect by measuring physical distances of loci, with a genomic distance of ~5 kb in single cells. This range of distances of approximately 100 nm is crucial for mediating the physical contact of transcription factors and other regulatory elements. Microscopy data delivered the complete distance distribution of two prototypic regions with regulatory active and inactive chromatin, respectively. Unexpectedly, we found very broad distributions of distances in both regions. Computer simulations of a coarse-grained model of these regions showed that the variance of the single-cell measurements can be explained only by the combinations of different influencing factors. This emphasizes the large cell-to-cell variance in the processes regulating chromatin compaction even in inactive regions.

INTRODUCTION

For almost 100 years, it has been known that interphase chromatin can be distinguished by means of light microscopy into less dense euchromatin and denser packed heterochromatin (1,2). Later, it became clear that nucleosomes are the basic building blocks organizing DNA packaging and are therefore central to the organization

Submitted August 19, 2021, and accepted for publication February 7, 2022.

[7]These authors contributed equally

*Correspondence: gero.wedemann@hochschule-stralsund.de or harz@biologie.uni-muenchen.de

Editor: Lars Nordenskiöld.

https://doi.org/10.1016/j.bpj.2022.02.009

© 2022 Biophysical Society.

This is an open access article under the CC BY license (http://creativecommons.org/licenses/by/4.0/).

of chromatin (3). Groundbreaking electron microscopic studies showed the tight interaction between histones and DNA, forming an 11-nm-thick fiber (4,5). Methodological advances have led to the view that chromatin has a rather irregular, heterogeneous organization (6–8). This view is supported by electron microscopic studies and super-resolution fluorescence microscopy that show interphase chromatin to be organized in a flexible and disordered structure in which regions with higher nucleosome density are interspersed with nucleosome-depleted regions (9–14).

The landscape of chromatin states is much more diverse than the originally described euchromatin and heterochromatin suggest. By analyzing genome-wide distribution patterns of chromatin-associated proteins, posttranslational histone modifications and DNase I hypersensitivity with algorithms such as ChromHMM and Segway, investigators have proposed up to 51 chromatin classes (15–21). DNase I hypersensitivity is a criterion that can also be used alone to subdivide chromatin in regulatory or active DNA with a high number of DNase I hypersensitive sites (DHS) as opposed to inactive regions with a low density of DHS (22,23).

Posttranslational histone modifications of the active chromatin classes, such as acetylation, may reduce nucleosome interaction strength and thus participate, among other mechanisms such as through ATP-dependent remodelers, in producing an open, less densely packed chromatin (24–28). Inactive classes are often characterized by methylation marks on histone 3 (e.g., H3K9me2/3), which can be bound by the heterochromatic protein 1, thereby compacting chromatin (29). However, large parts of inactive and more densely packed chromatin do not carry significant amounts of posttranslational histone modifications (15). Other mechanisms, such as the amount of linker histone H1, must therefore be responsible for the compaction (14).

A remarkable feature of chromatin is its dynamic nature, which has been observed in several fluorescence imaging studies (30–40) and is the reason for the large cell-to-cell variability in the structure of chromatin domains (41). Changes in nucleosome occupancy are actively regulated and can drastically affect the 3-dimensional (3D) genome architecture as it has been shown, for example, by the effects of tumor necrosis factor alpha on human endothelial cells (42). Even at the level of single nucleosomes, a significant and dynamic cell-to-cell variability can be found (43). The recently developed Fiber-seq method reveals that regulatory elements are actuated in an all-or-none fashion, thereby replacing a canonical nucleosome (44). Some ATP-dependent chromatin remodelers, and probably also some pioneer transcription factors, are known to exhibit nucleosome eviction activity (45–47). Together, these examples show that, depending on the regulatory context, the number and exact position of nucleosomes in active chromatin of eukaryotes can dynamically change.

Computational studies show a close link between nucleosome positions and the spatial organization of chromatin (48), which was explored by applying computer simulations of a coarse-grained model by many groups (e.g., (49–51)). These studies demonstrate, for example, that different nucleosome repeat lengths are responsible for more open or closed chromatin configurations (14,52). Moving even a single nucleosome can strongly influence the spatial organization (53). Thus, including the real length of the different linker DNA into coarse-grained models is required to obtain realistic results (14,53).

In our research, we investigated structural differences between active and inactive 5-kb chromatin segments of prototypical chromatin regions, selected on the basis of the presence or absence of DNase I hypersensitivity, using oligonucleotide-based fluorescence in situ hybridization (oligoFISH). By measuring the distance between labeled endpoints with systematic 3D stimulated emission depletion (STED) microscopy and comparing these data with Monte Carlo simulations of a coarse-grained model (53,54), we aimed to find underlying organizational principles. In active chromatin, simulated data match the microscopic data well, assuming cell-to-cell variability in nucleosomal occupancy. For inactive chromatin, variability of the maximal strength of the internucleosomal interaction and the binding of the linker histone H1 must be assumed to match the width of the distribution. Regardless of whether chromatin is active or inactive, our results reveal two striking features for 5-kb segments: (1) all distance distributions are right-tailed, and simulations indicate an underlying cell-to-cell variance in chromatin organization, and (2) distributions cover a wide range of distances from less than 50 nm to more than 200 nm.

MATERIALS AND METHODS

For a more detailed description of the methods and procedures described in this section, please refer to the supporting material's and methods.

Cell culture

Human erythroleukemia K562 cells (ATCC: CCL243) received from the Stamatoyannopoulos lab were grown in RPMI 1640 medium (Sigma, USA) supplemented with 10% fetal bovine serum (Sigma, USA) and 1% v/v penicillin/streptomycin (Sigma, USA) in cell culture flasks. Cells were cultured at 37°C in a humidified atmosphere containing 5% CO_2 and regularly tested for mycoplasma contamination.

Selection criteria for genomic regions

Universally active and inactive genomic regions were assessed by using the index of consensus DHSs of Meuleman et al. (22) derived from DNase I hypersensitive regions in 733 human biosamples encompassing 438 human cell and tissue types and states. We identified genomic regions with statistically significant enrichments of cleavage activity in DNase-seq experiments by using the program hotspot2 (55). The selected active region (chr11: 119,075,000–119,125,000) is spanned by a diverse set of genes, whereas the inactive region (chr11: 55,810,260–55,840,940) has a minimal number of elements overlapped by RepeatMasker.

Sample preparation and microscopy

Oligonucleotide probes for STED microscopy: We tiled 30 non-overlapping oligonucleotides (40-mers) across each target region (1.5–2 kb), selected for uniqueness and a higher density than afforded by other published design tools optimized for whole-genome coverage or chromosome walking (56–58). Oligonucleotides were labeled with ATTO 594 or ATTO 647N (LGC Biosearch Technologies, USA). A list of all of the oligonucleotides used is provided in Table S1 in the supporting material's and methods. FISH of formaldehyde-fixed K562 cells was carried out as previously published (41) with adaptations.

STED microscopy was carried out on a 3D STED microscope (Abberior Instruments, Germany) equipped with 2 pulsed excitation lasers (594 nm, 0.3 mW and 640 nm, 1.2 mW), 1 pulsed depletion laser (775 nm, 1.2 W), and Avalanche photodiodes for detection. A 100× UPlanSApo 1.4 NA oil immersion objective (Olympus, Japan) was used for all of the acquisitions. Pairs of FISH spots labeled with different dyes were detected in confocal scans, and high-resolution STED detail stacks were acquired only around these points of interest.

Image data analysis

Supervised machine learning was used as a quality control step to automatically classify STED stacks into "good" or "bad." Detailed spot analysis was performed on the analyzable good data to determine the coordinates of both FISH spots in their respective STED channels. The algorithm searched for the spot pair with the brightest signal by using a Laplacian-of-Gaussian blob detector and saved their subpixel coordinates derived from fitting a multidimensional Gaussian using the Levenberg-Marquardt algorithm for further statistical analysis. For measurements with 2D depletion, 3D coordinates were transformed into projected 2D coordinates by omitting the z coordinate.

Coarse-grained modeling

Simulation software: The software was developed in the Wedemann group in the last few decades and used in many studies. It was written in C++ and was adapted for the use of shared-memory parallel architectures according to the OpenMP standard. The replica exchange algorithm was implemented for distributed memory architectures using Message Passing Interface. The software cannot be made public at the moment, since it contains code under copyright by other parties.

Simulation protocol: A Monte Carlo (MC) algorithm was used to create a statistically relevant set of configurations satisfying the Boltzmann distribution (59). To overcome local energy minima (54), we applied a replica exchange procedure introduced by Swendsen and Wang (60). Here, M replicas of the system were simulated with Metropolis MC simultaneously, each at a different temperature, T_i. After a fixed number of MC simulation step replicas with adjacent temperatures (T_i, T_{i+1}), the temperature is swapped with the probability (Eq. 1):

$$\min[1, \exp(-(\beta_i - \beta_{i+1})(E_{i+1} - E_i))], \tag{1}$$

with $\beta_i = 1/(k_B T_i)$, k_B being the Boltzmann constant and E_i the energy (e.g., elastic energies), of the system i. Before the simulations, the set of temperatures was determined using a feedback-optimized approach (61). This algorithm optimizes the distribution of temperatures iteratively, such that the diffusion of replicas from the highest to the lowest temperature and vice versa is improved in each iteration. The procedure is more efficient when starting with a system that is pre-relaxed using a simulated annealing approach (54). Simulation parameters and constants are given in Table S2.

We chose 16–60 replicas, depending on the system. For systems with 4 kT as a maximum value of internucleosomal interaction energy, we computed at least 10×10^6 MC steps and 90×10^6 steps for 6 kT per replica after simulated annealing. For checking the convergence, we analyzed the end-to-end distance and the energy as parameters. To determine the point when equilibrium was reached, we analyzed visually the plots with the number of steps on a logarithmic scale. Only configurations after that point were used in the analysis. From analysis of the autocorrelation of energy and end-to-end distance, we estimated that configurations are uncorrelated after 10×10^3 steps for systems with 4-kT maximum interaction strength and 20×10^3 steps for 6 kT (see supporting material's and methods). This leads to 1000–2000 uncorrelated configurations. See Table S3 for all of these values of every simulation in the supporting material.

Modeling of 3D configurations: Since atomistic modeling of chains with many nucleosomes is not possible, coarse-grained models are widely used. We applied the simulation procedure as described in Muller et al. and followed the description given there (53). Chromatin is modeled as a chain of segments, in which spherocylindrical units describing the nucleosomes are connected by cylindrical segments describing the linker DNA. Each segment i possesses a position and a local coordinate system consisting of three perpendicular unit vectors ($\widehat{u_i}$, $\widehat{v_i}$, $\widehat{f_i}$) that describe its torsional orientation (Fig. S1). Vector $\widehat{u_i}$ is parallel to the direction of the segment (i.e., the vector $\vec{s_i}$ from its position to the position of the next segment). The position of the center of the nucleosome and its orientation is computed from the center of the nucleosome segment by the length d and six angles describing the relative orientation (Fig. S2). Systems without linker histone and with linker histone differ by the set of angles (62). The length of each individual linker DNA was computed from the positions of the nucleosomes in the studied region. The number of base pairs of a linker length is converted to nanometers by a factor of 0.34 nm/bp. Each linker DNA is modeled by at least 2 segments. If the linker length is larger than 20 nm, then the number of segments is calculated by rounding up (linker length/10 nm).

Statistical analysis

A mixture histogram (Fig. 4 *H*) was calculated by minimizing the squared differences between the bins of a histogram of the microscopically measured FISH spot distances (Fig. 4 *A*) and a linear combination of the histograms of simulation results with varying nucleosome occupancies (Fig. 4 *B*–*G*). Quadratic programming (via the quadprog package in R) was used to find a solution in which the contributions of the individual simulations are non-negative and equal 1.

2D and 3D distance data were cut off at the maximum length of a theoretical beads-on-a-string fiber, since it is very unlikely that genomic regions more elongated than a fully stretched beads-on-a-string fiber are present in the nucleus. To calculate the length of a beads-on-a-string fiber, the following formula was used: genomic length [bp] * 0.34 nm (size of 1 base)/7 (63). For 5-kb genomic distances, the cutoff for measured distances was at 250 nm.

Data availability

All of the data are available through the public Open Science Framework repository: https://doi.org/10.17605/OSF.IO/ZJWXM. Simulation trajectories are in an easily readable XML format (64). Analysis and visualization scripts are available as a directly runnable code ocean capsule: https://codeocean.com/capsule/8421512/tree/v2.

RESULTS

Chromatin organization of active and inactive chromatin was analyzed in K562 cells using systematic super-resolution microscopy of DNA sequences labeled with oligoFISH probes and comparison with simulated 3D chromatin configurations generated by a coarse-grained model. The

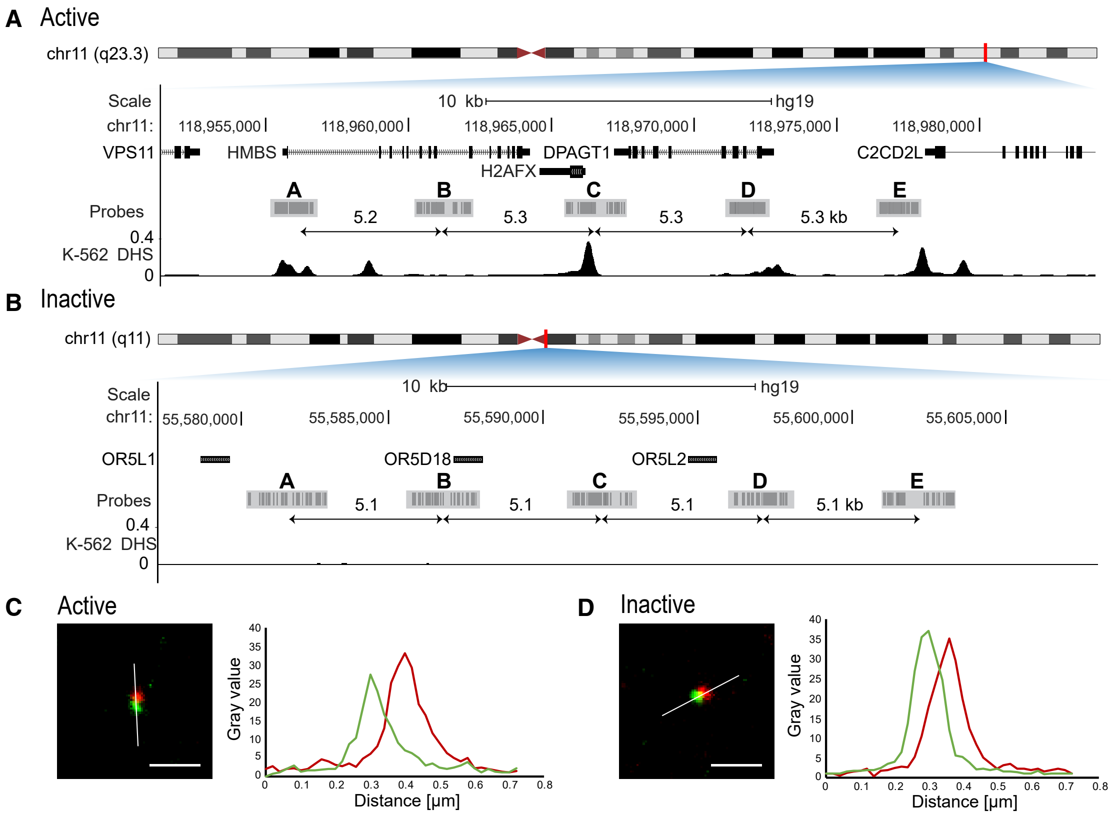

FIGURE 1 FISH probe design for active and inactive region. (*A*) The active region contains the genes *HMBS*, *H2AFX,* and *DPAGT1*. The probe sets are almost equally spaced (5.2, 5.3, 5.3, and 5.3 kb midpoint to midpoint) and mostly cover DHS sites. (*B*) The inactive region contains genes for olfactory receptors. The region shows no DNase I hypersensitivity, and the probe sets are equally spaced (5.1 kb midpoint to midpoint). Modified University of California, Santa Cruz genome browser plot (68); the data for the DNase I track are from GEO: GSM816655. (*C* and *D*) Representative STED detail images of FISH spots in 2 colors for active (*C*) and inactive (*D*) (target 1 in green, target 2 in red). Line plots depict intensity values for both colors along lines of interest (*white lines*). Scale bar, 500 nm. To see this figure in color, go online.

K562 cell line is well suited to computer simulations as a wealth of information such as genome-wide chromatin immunoprecipitation sequencing data, comprehensive maps of posttranslational nucleosome modifications, and nucleosome positioning generated by the ENCODE project are available (21,65).

STED microscopy as a tool to study prototypic chromatin regions on the kilobase scale

By using data from Meuleman et al. (22), we selected a 20-kb region on chromosome 11 (hg19, chr11: 118955404–118977871), which exhibits very high density of DHSs, not only in K562 (Fig. 1 *A*) but also in more than 730 samples from human cells and tissues. Moreover, this region is flanked upstream and downstream by highly active chromatin. For inactive chromatin, the selection criteria were a minimal number of repetitive elements and missing DHS over 30 kb in more than 730 human samples. In K562 cells, the region without DHSs spans over 2 Mb. The selected 20-kb inactive region is also located on chromosome 11 (hg19, chr11: 55580425–55603312) (Fig. 1 *B*). For each of these 20-kb regions, 5 oligoFISH probe sets (A, B, C, D, E; Fig. 1, *A* and *B*) were designed, dividing the 20 kb into 4 approximately 5-kb-long segments from midpoint to midpoint of the respective probe set (probe set combinations: AB, BC, CD, DE). Each oligoFISH probe set consisted of 30 oligonucleotides (directly fluorescently labeled 40mers) covering a region of approximately 1.5–2 kb (Fig. 1, *A* and *B*). These small genomic distances are expected to result in spatial distances falling below the resolution limit of light microscopy (66), which is more than 200 nm in the x- and y-dimensions and >500 nm in the z dimension (67). Using STED microscopy, we achieved a root mean square precision for the distance measurements between two spots with a different spectral behavior of approximately 7.5 nm in 3D (supporting material's and methods) (Fig. 1, *C* and *D*). By using reconstituted chromatin, we showed that STED microscopy can resolve distances between the ends of chromatin consisting of ∼5 kb DNA and up to 25 nucleosomes (Fig. S3). The distances measured microscopically were in the range of a simulated distance distribution of the same system.

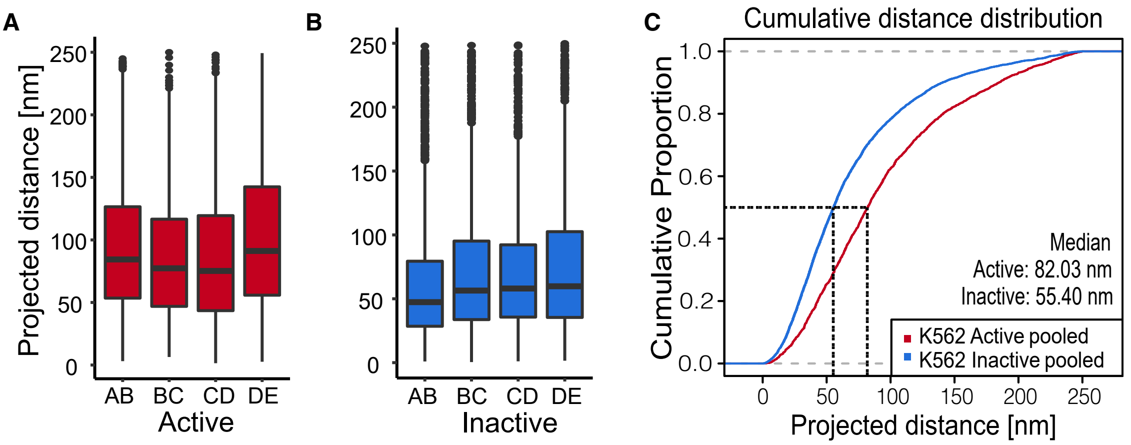

FIGURE 2 2D STED distance measurements showed increased compaction in the inactive region versus the active region. (*A*) Boxplot for the active region for all 4 measured intervals (AB: n = 672, BC: n = 540, CD: n = 484, DE: n = 566; n = number of single-cell measurements pooled from 3 independent replicates). (*B*) Boxplot for the inactive region for all 4 measured intervals (AB: n = 1585, BC: n = 1621, CD: n = 1200, DE: n = 1395; n = single-cell measurements from 3 independent replicates). (*C*) All of the data from active (*A*) and inactive (*B*) regions were pooled to generate a cumulative distribution. The cumulative distribution of measured distances showed differences in distributions between active (*red*) and inactive (*blue*). The median is the value at the 50% proportion (*black dashed line*). For the active region, the median is 82 nm and for the inactive region, it is 55 nm. To see this figure in color, go online.

Inactive regions are more compact than active regions

Recent studies reveal a high cell-to-cell variance of the spatial genome organization (69–71). To study the chosen regions, we applied high-throughput 2D STED microscopy to generate data with high statistical power characterizing the nanoscale organization of 5-kb segments of active and inactive chromatin. For each of the 8 investigated 5-kb segments, between 484 and 1621 single-cell measurements were analyzed. The four measured intervals in the active chromatin region differ from one another. We found some significant deviations, with the maximum difference in the median projected distance of 16 nm ($p = 0.00053$, BC versus DE and CD versus DE, Wilcoxon rank-sum test) (Fig. 2 *A*; Table S4). In active chromatin, variability of the nanoscale organization is expected since each 5-kb segment is composed of different proportions of exons, introns, enhancers, and other regulatory sequences. Surprisingly, we also found highly significant differences between the investigated intervals in inactive chromatin. We expected much less difference in compaction because inactive chromatin is expected to be more uniform as it does not harbor active regulatory elements and nucleosome occupancy is not modified by transcriptional activity (Figs. 2 *B* and S4). The maximum difference in the median projected distance was 12 nm within the inactive chromatin group ($p < 0.0001$, AB versus DE, Wilcoxon rank-sum test; Table S4).

However, since the differences within the active and inactive regions are small, they were pooled to show the overall length distribution of each chromatin class. The median projected distance between 2 FISH spots flanking a typical 5-kb interval of active chromatin is 82 nm and 55 nm in inactive chromatin (Fig. 2 *C*). Shorter double spot distances indicate a higher degree of chromatin compaction, whereas larger distances suggest less compaction. Thus, data from our measurements are in line with published data showing active chromatin to be less compacted compared to inactive chromatin (11). As expected, the distributions of the FISH spot distances of active and inactive chromatin differ significantly as shown in a cumulative distribution plot (Fig. 2 *C*, $p < 2 \times 10^{-16}$, Wilcoxon rank-sum test; Table S4).

For a more in-depth analysis, we selected a 5-kb segment for both the active and inactive regions, which are representative of the respective group in 2D STED measurements. We chose interval AB for the active region and CD for the inactive region (Fig. 2, *A* and *B*). Both regions do not show CCCTC-binding factor (CTCF) binding sites and are therefore not anchors for chromatin loops.

Assigning the input parameters for coarse-grained modeling

The exact position of nucleosomes is an important input parameter for coarse-grained models and strongly affects simulated configurations (14,53). Nucleosomal positioning can be determined by micrococcal nuclease digestion followed by deep sequencing (MNase-seq) (72). Here, we used ENCODE MNase-seq tracks of K562 cells, which are derived from cell populations and therefore often show a seemingly overlapping nucleosome pattern (University of California, Santa Cruz accession: wgEncodeEH000921, GEO accession: GSM920557). These data are unsuitable for our coarse-grained model, as it requires non-overlapping unique nucleosome positions as input. Therefore, we computed the most probable non-overlapping nucleosome populations by applying the NucPosSimulator (73).

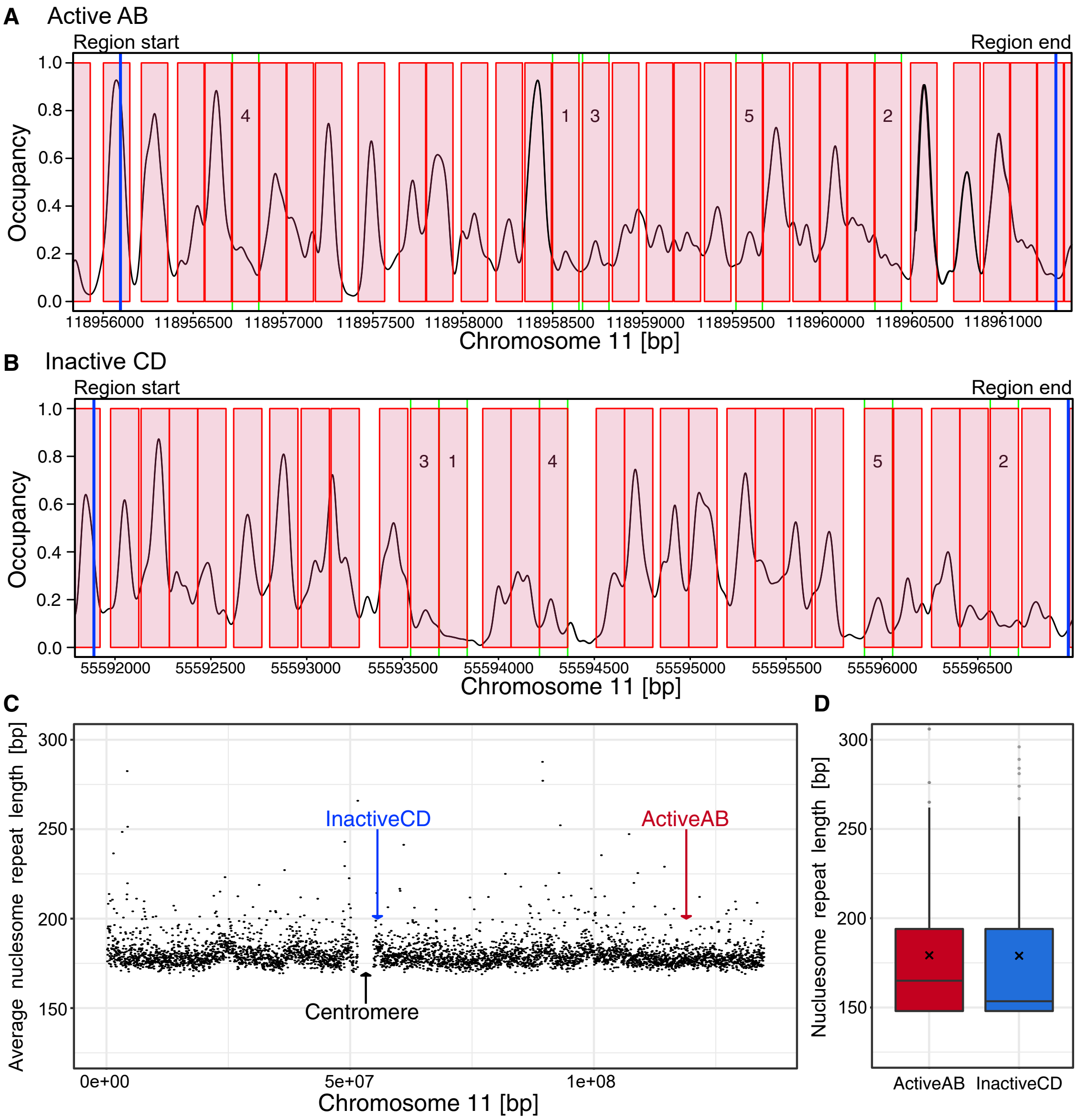

FIGURE 3 Nucleosome positions and nucleosome repeat lengths were calculated using the NucPosSimulator. Nucleosome positions (*red boxes*) for active AB (*A*) and inactive CD (*B*) were based on MNase-seq occupancy tracks (*black line*). The blue lines indicate the start and end of the investigated region. Numbers in boxes indicate the ranking of the 5 nucleosomes with the lowest nucleosome occupancy signal. (*C*) The mean values of the NRL of a sliding window of 30,000 bp. Values larger than 300 and windows with fewer than 3 nucleosomes were omitted. The mean NRL for chromosome 11 was 183.4 ± 66.3 bp. (*D*) Investigated active and inactive regions as marked in the plot (*arrows in C*) have a mean of 179.6 and 179.1 bp, respectively (black x). To see this figure in color, go online.

Experimentally derived nucleosome occupancy and the computed most probable nucleosome positions of active region AB and inactive region CD are shown in Fig. 3, *A* and *B*. Nucleosome positions of the respective flanking regions can be found in Fig. S4. We identified 28 nucleosomes in the active region and 29 nucleosomes in the inactive region. The flanking regions contain approximately 110 nucleosomes on each side. A list of the lengths of all linker DNA can be found in the Open Science Framework repository. For the nucleosomal repeat length (NRL) of chromosome 11, we calculated a mean value of 183.4 ± 66.3 bp applying NucPosSimulator (Fig. 3 *C*) (for calculation details, see the Materials and methods section). The mean NRL of the active (AB) and inactive (CD) region studied in detail is 179.6 and 179.1 bp, respectively (Fig. 3 *D*). Both values are in the range of the NRL of chromosome 11. To cross-check the effects of possible inaccuracies in the positions of the nucleosomes, additionally we determined the nucleosome positions for both regions and flanking regions from lymphoblastoid cell lines (74) and used them for control simulations.

The strength of the internucleosomal energy is another important parameter in all coarse-grained models and depends on the solvent (75) and histone modification (71). Literature values for this energy typically range from 3 to 10 kT (71,76,77). Nucleosomes containing unmodified histones have a higher interaction energy, whereas modifications such as acetylation weaken internucleosomal interactions (71). Since the inactive chromatin examined here does not exhibit significant histone modifications (Fig. S4 *B*), we have used a value from the upper range of the literature values (6 kT) to simulate this chromatin type. Conversely, the active region features many posttranslational histone modifications (Fig. S4 *A*), and we thus used a lower value (4 kT) to compute the respective configurations.

The nucleosome occupancy varies from cell to cell in active chromatin

The microscopic data shown so far are 2D data, which underestimate the real 3D distances between the FISH spots since the cells are expected to be rotated randomly relative to the optical axis of the microscope. Only 3D single-cell microscopy allows the study of real distances between 2 spots on a single-cell level and to compare data between microscopy and simulation. Therefore, we performed 3D STED measurements, which require careful correction for refractive index mismatch between the immersion fluid of the objective lens and the embedding medium (see supporting material's and methods).

The 3D STED measurements for the 5-kb AB interval in the active chromatin region revealed distances ranging from <50 to 250 nm, with a mean distance of 115 nm ($n = 762$; Fig. 4 *A*; data of all other segments Fig. S5; statistical data in Table S5). Remarkably, in active chromatin, elongated configurations can be found, which results in a right-tailed distribution of the microscopic distance measurements. To understand this phenomenon better, we performed coarse-grained computer modeling of the nucleosome chain with the most probable nucleosome positions. We sampled a statistically relevant ensemble of independent 3D configurations in the active region by applying our coarse-grained model, which included elastic and electrostatic properties as well as excluded volume effects. To compare the simulated data with the microscopic data, the distances between the simulated sequence segments that correspond to those of the microscopic measurements were determined. In this way, a distance histogram was generated from the simulated data, which can be directly compared to the microscopic data (Fig. 4, *B–G*). The computed distribution was narrower, and the mean distance was approximately a standard deviation shorter than the microscopically measured distribution (Fig. 4 *B*).

We hypothesized that in the cell population used for the microscopy experiment, the number of bound nucleosomes varies from cell to cell. This hypothesis was tested by computer simulations, in which the least probable nucleosomes were removed. To find the nucleosomes with the lowest occupancy signal, we analyzed the mean value from the occupancy data calculated by NucPosSimulator (nucleosomes with lowest occupancy signal are indicated in Figs. 3 *A* and S4). Next, we computed statistically relevant ensembles of 3D configurations by replacing the nucleosome with the lowest occupancy signal by naked DNA (−1, Fig. 4 *C*). The same was done by replacing two (Fig. 4 *D*), three (Fig. 4 *E*), four (Fig. 4 *F*), and five (Fig. 4 *G*) nucleosomes according to the rank order of the nucleosome occupancy signal. In fact, a reduction of the total nucleosome number resulted in increasingly larger mean distances, but none of the individual distributions were comparable with the microscopically measured distribution. By applying a least squares fit, the different distance distributions were combined and resulted in a mixed distance histogram that mimics the microscopic data better than each of the underlying histograms, as indicated by a reduction in the root mean square error (Fig. 4 *H*) (see Materials and methods). Visualizations of simulated chromatin configurations show that both fibers with all nucleosomes and with a reduced nucleosome number (−5) can have short and long end-to-end distances (Fig. 4, *I* and *J*). These configurations show local accumulations of a few nucleosomes connected by stretches with low nucleosome occupancy. These structures are remarkably similar to recently published light and electron microscopic data of interphase chromatin (12,14,78). These results from the models are robust against possible inaccuracies in the nucleosome positions since computer simulations with nucleosome positions derived from lymphoblastoid cell lines deliver nearly identical results (Fig. S6, *K* and *L*). Linker histone H1 does not change the distance distributions in this case either (Fig. S6, *I* and *J*).

A process that is obviously accompanied by major changes in chromatin structure and in which nucleosomes are also temporarily removed from the chromatin structure is DNA replication in the S phase of the cell cycle. Fluorescence-activated cell sorting of cells with fluorescently labeled DNA was used to generate G1, S, and G2 phase fractions for further analysis (Fig. S7). Microscopically measured distance distributions of G1 and S phase cells resemble the data of the unsorted population.

Inactive region is compacted by various mechanisms

Microscopic data of the inactive region CD show the expected shift of the histogram to shorter distances, indicating more condensed chromatin (Fig. 5 *A*). Similar to the active chromatin, the histogram of the inactive region also contains large distances that cannot be explained by replicating DNA (Fig. S7). 3D STED distance histograms of the inactive

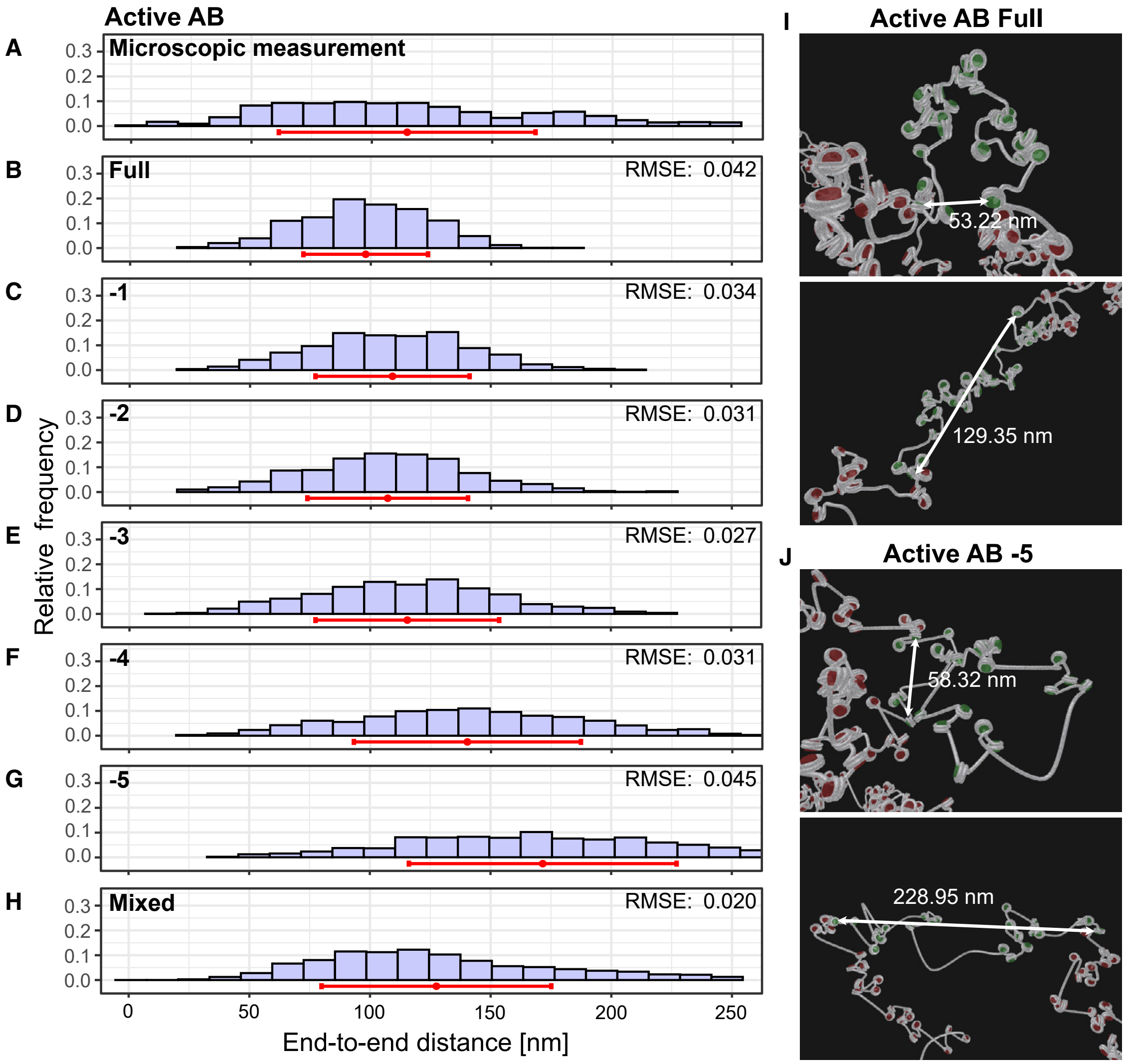

FIGURE 4 Distance distributions from microscopic experiments and from computer simulations of the active region. (*A*) 3D STED measurements of active AB result in a distance distribution ranging from <50 to 250 nm, with a mean of 115 $\pm$ 53 nm (n = 762 single-cell measurements from 3 independent replicates). (*B–H*) For computer simulations, results are shown for the region active AB with all nucleosomes (*full*) (*B*), with 1–5 nucleosomes replaced by naked DNA (*C–G*) and a combined plot (*H*). The mean value (*red dot*) and standard deviation (*red line*) are shown for each distribution. In the combined plot (*H*), the distributions have a weight of 0.45, 0.00, 0.00, 0.00, 0.31, and 0.24 (from all nucleosomes to -5 nucleosomes). (*I* and *J*) Example images of simulated chromatin fibers for active region AB (*green nucleosomes*), with all nucleosomes (*I*) and with -5 nucleosomes (*J*) and the adjacent sequences (*red nucleosomes*). The upper image in (*I*) and (*J*) shows a configuration resulting in a short end-to-end distance indicated by a white arrow; the lower image depicts a large end-to-end distance. RMSE, root mean square error of simulated histogram bins in comparison to the measured data. To see this figure in color, go online.

region CD were compared with simulated data by the same strategy as above. The comparison showed that the computed mean distance was ~40 nm larger than the microscopically measured distance when a maximal attractive internucleosomal energy of 4 kT was used for the simulation (Fig. 5, *A* and *B*). As argued earlier, an increase in the interaction energy to 6 kT seems to be more realistic for simulating inactive chromatin. This approach delivered configurations with the mean value of the simulated distance distribution in the correct range but symmetrical and not skewed to smaller values (Fig. 5 *C*). Obviously, additional mechanisms compact the inactive chromatin of the investigated region.

Genome-wide data on the level of H3K9me3 (GEO: GSM733776) and H3K27me3 (GEO: GSM733658) histone modifications show in the inactive region CD only background levels, which can be found throughout the genome. Also, repetitive DNA sequences (RepeatMasker) are not enriched. The levels of these markers are significantly lower than in regions known to be compacted by heterochromatinization or by binding the Polycomb group proteins. Therefore, other mechanisms must be considered,

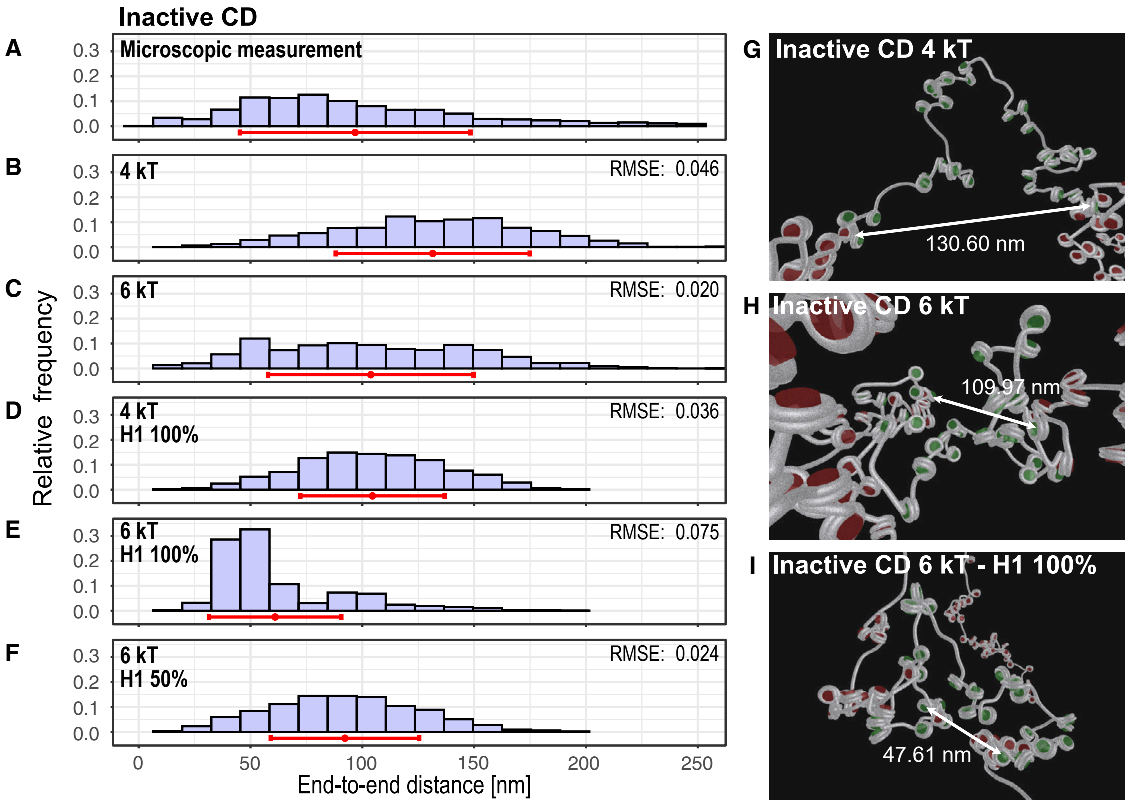

FIGURE 5 Distance distributions from microscopic experiments and from computer simulations of the inactive region. (*A*) 3D STED measurement of inactive CD results in a right-tailed distance distribution, with the mass of the distribution toward shorter distances and a mean of 97 ± 52 nm (n = 1320 single-cell measurements from 3 independent replicates). (*B–F*) Computed distance distributions with different maximal internucleosomal interactions (4 kT [*B*, *D*] and 6 kT [*C, E, F*]), without (*B*, *C*) linker histone H1 or with (*D*, *E*) H1 (100% of nucleosomes occupied), and a random distribution of binding of 50% H1 (*F*). The mean value (*red dot*) and standard deviation (*red line*) are shown for each distribution. (*G–I*) Visualizations of simulated configurations. RMSE, root-mean-square error of simulated histogram bins in comparison to the measured data. To see this figure in color, go online.

such as the binding of linker histone 1 (H1), which has long been known to have a chromatin-compacting effect (14,79). H1 is included in the computer model by different angles of the attached linker DNA at the nucleosomes (52). These angles were derived by a systematic analysis of data from reconstituted fibers (62). It can be expected that details of the angles vary since the chicken linker histone H5, for example, causes different angles than human H1 (62). However, all of the variants of H1 lead to higher chromatin compaction.

In fact, simulations with a stochiometric H1 to nucleosome ratio of 1:1 led to more compact configurations, with a mean value of 27 nm less for 4 kT (Fig. 5 *D*) and 43 nm less for 6 kT (Fig. 5 *E*). To explore the effects of different stoichiometry of H1, we performed computer simulations of a random 50% nucleosome binding (1:2). Here, the compaction is less pronounced, and the mean is approximately 12 nm smaller than without H1 (Fig. 5 *F*). Visualizations of exemplary simulated configurations are shown in Fig. 5, *G–I*.

In summary, similar to the active region the experimental data of the inactive region can only be explained by a cell-to-cell variability but including a stronger internucleosomal interaction and binding of H1.

DISCUSSION

By using high-throughput super-resolution microscopy, we studied the nanoscale organization of 5-kb chromatin segments that are located in regulatory active and inactive chromatin. The data shown here contain information that differs for fundamental reasons from that of published conformation capture data sets such as Hi-C. While microscopy measures the physical distances between genomic loci, conformation capture methods assess how often direct contacts between genomic elements occur (80). Most conformation capture data sets represent a population average, whereas we provide here statistically robust data on the chromatin configuration in single cells that can be directly compared with data from simulations. The selected areas are prototypic for active and inactive chromatin because patterns of prominent or absent DHSs spread over hundreds of kilobases around the selected region and can be found in more than 730 different human cell and tissue samples. Both regions have an NRL close to the average

of the entire chromosome, which is another indication that representative regions were selected. For these reasons, we assume that the structural principles described apply to other parts of the genome.

In both active and inactive chromatin, 3D spatial distances between the endpoints of the 5-kb segments differ from cell to cell, resulting in a broad distance distribution, with the mass of the distribution shifted more to shorter values in inactive chromatin. In contrast, simulations with different nucleosome occupancies, changed strength of the internucleosomal energies, or deviations from stoichiometric H1 binding led to far narrower distance distributions. Therefore, the large width of the distance distribution seems to be a feature that is caused by the summation of cell-to-cell differences in the resulting histogram.

Unexpectedly, we found very elongated chromatin configurations with 5-kb exhibiting lengths of over 200 nm in both active and inactive chromatin. For comparison, a stretched beads-on-a-string chromatin fiber of 5 kb has a length of 243 nm (63). Replication cannot account for the majority of these elongated configurations as we have shown by measurements on cells in G1 phase. Replication is a fast process, occurring at ~16 bp/s (81), so the probability of fixing a cell at the moment a 5-kb segment is currently replicated is low. The same is true for other active mechanisms such as loop extrusion by cohesion (82). In simulations with our coarse-grained model, elongated chromatin configurations are more probable if a number of nucleosomes are replaced by naked DNA. Therefore, it is important to investigate which nucleosomes have the weakest occupancy in our model. In fact, 8 of the 10 nucleosomes with the lowest occupancy in the active region are localized within DHSs (Fig. S4 *A*), a result that is consistent with genome-wide measurements (44).

The perspective of cell-to-cell differences in nucleosome occupancy in active DNA is supported by different lines of evidence: (1) While at certain positions nucleosomes are positioned with high precision (83), nucleosome positions can vary substantially from cell to cell (43,73), (2) pioneer transcription factors and chromatin remodeling complexes can change nucleosome occupancy (84,85), (3) the upregulation of genes is known to reduce the number of bound nucleosomes (42) and increases H2B mobility (14), (4) transcription factors compete cooperatively with nucleosomes for access to DNA (86,87), and (5) regulatory elements are actuated in an all-or-none fashion by the cooperative binding of transcriptional factors in place of a canonical nucleosome (44,88).

For most of the 1600 known transcription factors (89), there are no models to estimate the DNA structure after binding. We simulated regions without nucleosomes as linker DNA with the corresponding elastic and electrostatic properties, since 92% of the transcription factors studied have a DNA footprint between 7 and 30 nt (90), while nucleosomes have a footprint of 146–147 nt and the DNA is 1.65× wrapped around them (5). Therefore, we can neglect bound transcription factors in the model without limiting the conclusions.

As described earlier, the microscopic measurements of inactive chromatin revealed a compaction that can be explained by an increase in the strength of internucleosomal interactions and by the additional introduction of the linker histone H1. Microscopic measurements showed that the inactive region investigated here is expected to be embedded in a more compact chromatin environment (Fig. S8). It can be speculated that this higher density is caused by the increased internucleosomal interaction strength as found in the model. This result is in line with the current discussion of phase separation in the nucleus (91).

Similar to the active regions, microscopic data of the inactive region also show elongated chromatin configurations (>200 nm) in individual cells. In analogy to active chromatin, this could indicate variable nucleosome occupancy in inactive chromatin as well. In fact, the data shown in Fig. 3 *B* also show weakly bound nucleosomes in this chromatin class, but this does not exclude further mechanisms causing elongated chromatin configurations. Regardless, the large variation in physical distances between spots with a genomic distance of 5 kb from cell to cell suggests that inactive chromatin is also subject to continuous reorganization.

In each computer simulation, a system has a certain number of nucleosomes, amount of bound H1, and internucleosomal interaction strength. It can be expected that *in vivo* more variety exists (e.g., in the active region, one or two nucleosome are missing and a varying stoichiometry of linker histone is present). The properties of these systems are expected to be in the range of the already-broad range of different simulated systems presented here.

An extensive body of literature (for a review, see Schoenfelder and Fraser (92)) on chromatin architecture focuses on the formation of chromatin loops, bringing regulatory elements into close contact and thus regulating gene expression. Distances below which an enhancer is thought to activate a promotor range from less than 150 nm (66) to 300 nm (93). Here, we show by high-throughput microscopy of human chromatin that in active regions more than 45% of the 5-kb endpoints approach to less than 100 nm, whereas in inactive chromatin, this is the case in more than 60% of the cells (value derived from data of Figs. 4 *A* and 5 *A*). Apparently, thermodynamically driven spontaneous movements can bring regulatory elements into close contact with their promoters that are only a few kilobases distant from one another. Considering that in the human genome, 142,000 enhancer-like elements fall within 2 kb from the nearest transcription start site (21), such spontaneous movements of chromatin could significantly influence gene regulation.

SUPPORTING MATERIAL

Supporting material can be found online at https://doi.org/10.1016/j.bpj.2022.02.009.

AUTHOR CONTRIBUTIONS

This study was conceived and supervised by H.H., G.W., J.A.S., and H.L. K.B. performed all of the microscopic experiments, including sample preparation and STED imaging shown in Figs. 1, 2, 4 *A*, 5 *A*, S5, and S8. Work on reconstituted chromatin (Fig. S3) was completed by C.S., who was responsible for the molecular biology; T.B. prepared the salt gradient dialysis chromatin under the supervision of P.K.; D.H., M.G.-O. and C.S. performed the imaging. Cell-cycle measurements (Fig. S7) were performed by M.G.-O. and G.S. (imaging) and J.S. (fluorescence-activated cell sorting). E.R. selected the investigated genomic regions, and E.H. provided the computational tools for the oligo probe design. T.R. designed the oligo probes and supervised the FISH experiments. K.B. analyzed and interpreted the published ENCODE genome browser data, with the assistance of T.R. (Fig. S4). D.H. provided the computational tools for microscope automation and image analysis. T.Z. performed the computational modeling, with input from G.W. T.Z. analyzed the nucleosome position from MNase data, prepared and executed all of the simulations, and analyzed the simulation data as 3D visualizations and histograms (Figs. 3; 4, *B–J*; 5, *B–I*; S1; S2; S3, *D*; and S6, *B–L*). The manuscript was written by H.H. and G.W., with support from all of the authors.

ACKNOWLEDGMENTS

This work was supported by grants by the Deutsche Forschungsgemeinschaft Priority Program (SPP 2202/422857584, to H.H. and H.L.; SPP 2202/422780392 to G.W.), and SFB1064 (to P.K.), and by a grant from the National Human Genome Research Institute (RM1- HG007743-02CEGS–Center for Photogenomics, to J.A.S. and H.L.) G.W. was supported by the North-German Supercomputing Alliance (HLRN, mvb00012). The authors acknowledge the North-German Supercomputing Alliance (HLRN) and the Leibniz-Rechenzentrum (LRZ) for providing high-performance computing resources that have contributed to the research results reported in this article. K.B. and C.S. were supported by the International Max Planck Research School for Molecular Life Sciences (IMPRS-LS). Microscopic images were acquired from the microscopes of the Center for Advanced Light Microscopy (CALM) at LMU Munich. We thank Richard Sandstrom for help with the Hi-C data.

SUPPORTING CITATIONS

References (94–110) appear in the supporting material.

REFERENCES

1. Trojer, P., and D. Reinberg. 2007. Facultative heterochromatin: is there a distinctive molecular signature? *Mol. Cell.* 28:1–13.
2. Heitz, E. 1928. Das heterochromatin der Moose. *Jahrb. Wiss. Bot.* 69:762–818.
3. Kornberg, R. D. 1974. Chromatin structure: a repeating unit of histones and DNA. *Science.* 184:868–871. https://doi.org/10.1126/science.184.4139.868.
4. Olins, A. L., and D. E. Olins. 1974. Spheroid chromatin units (ν bodies). *Science.* 183:330–332.
5. Luger, K., A. W. Mader, …, T. J. Richmond. 1997. Crystal structure of the nucleosome core particle at 2.8 A resolution. *Nature.* 389:251–260. https://doi.org/10.1038/38444.
6. Lakadamyali, M., and M. P. Cosma. 2020. Visualizing the genome in high resolution challenges our textbook understanding. *Nat. Methods.* 17:371–379. https://doi.org/10.1038/s41592-020-0758-3.
7. Maeshima, K., S. Ide, and M. Babokhov. 2019. Dynamic chromatin organization without the 30-nm fiber. *Curr. Opin. Cell Biol.* 58:95–104. https://doi.org/10.1016/j.ceb.2019.02.003.
8. Mirny, L. A. 2011. The fractal globule as a model of chromatin architecture in the cell. *Chromosome Res.* 19:37–51. https://doi.org/10.1007/s10577-010-9177-0.
9. Konig, P., M. B. Braunfeld, …, D. A. Agard. 2007. The three-dimensional structure of in vitro reconstituted Xenopus laevis chromosomes by EM tomography. *Chromosoma.* 116:349–372. https://doi.org/10.1007/s00412-007-0101-0.
10. Fussner, E., M. Strauss, …, D. P. Bazett-Jones. 2012. Open and closed domains in the mouse genome are configured as 10-nm chromatin fibres. *EMBO Rep.* 13:992–996. https://doi.org/10.1038/embor.2012.139.
11. Boettiger, A. N., B. Bintu, …, X. Zhuang. 2016. Super-resolution imaging reveals distinct chromatin folding for different epigenetic states. *Nature.* 529:418–422. https://doi.org/10.1038/nature16496.
12. Ou, H. D., S. Phan, …, C. C. O'Shea. 2017. ChromEMT: visualizing 3D chromatin structure and compaction in interphase and mitotic cells. *Science.* 357:eaag0025. https://doi.org/10.1126/science.aag0025.
13. Nir, G., I. Farabella, …, C. T. Wu. 2018. Walking along chromosomes with super-resolution imaging, contact maps, and integrative modeling. *PLoS Genet.* 14:e1007872. https://doi.org/10.1371/journal.pgen.1007872.
14. Gomez-Garcia, P. A., S. Portillo-Ledesma, …, M. Lakadamyali. 2021. Mesoscale modeling and single-nucleosome tracking reveal remodeling of clutch folding and dynamics in stem cell differentiation. *Cell Rep.* 34:108614. https://doi.org/10.1016/j.celrep.2020.108614.
15. Ernst, J., and M. Kellis. 2010. Discovery and characterization of chromatin states for systematic annotation of the human genome. *Nat. Biotechnol.* 28:817–825. https://doi.org/10.1038/nbt.1662.
16. Ernst, J., P. Kheradpour, …, B. E. Bernstein. 2011. Mapping and analysis of chromatin state dynamics in nine human cell types. *Nature.* 473:43–49. https://doi.org/10.1038/nature09906.
17. Filion, G. J., J. G. van Bemmel, …, B. van Steensel. 2010. Systematic protein location mapping reveals five principal chromatin types in Drosophila cells. *Cell.* 143:212–224. https://doi.org/10.1016/j.cell.2010.09.009.
18. Ram, O., A. Goren, …, B. E. Bernstein. 2011. Combinatorial patterning of chromatin regulators uncovered by genome-wide location analysis in human cells. *Cell.* 147:1628–1639. https://doi.org/10.1016/j.cell.2011.09.057.
19. Hoffman, M. M., O. J. Buske, …, W. S. Noble. 2012. Unsupervised pattern discovery in human chromatin structure through genomic segmentation. *Nat. Methods.* 9:473–476. https://doi.org/10.1038/nmeth.1937.
20. Hoffman, M. M., J. Ernst, …, W. S. Noble. 2013. Integrative annotation of chromatin elements from ENCODE data. *Nucleic Acids Res.* 41:827–841. https://doi.org/10.1093/nar/gks1284.
21. ENCODE Project Consortium, Moore, J. E., …, Z. Weng 2020. Expanded encyclopaedias of DNA elements in the human and mouse genomes. *Nature.* 583:699–710. https://doi.org/10.1038/s41586-020-2493-4.
22. Meuleman, W., A. Muratov, …, J. Stamatoyannopoulos. 2020. Index and biological spectrum of human DNase I hypersensitive sites. *Nature.* 584:244–251. https://doi.org/10.1038/s41586-020-2559-3.
23. Gross, D. S., and W. T. Garrard. 1988. Nuclease hypersensitive sites in chromatin. *Annu. Rev. Biochem.* 57:159–197. https://doi.org/10.1146/annurev.bi.57.070188.001111.
24. Gorisch, S. M., M. Wachsmuth, …, K. Rippe. 2005. Histone acetylation increases chromatin accessibility. *J. Cell Sci.* 118 (Pt 24):5825–5834. https://doi.org/10.1242/jcs.02689.
25. Moller, J., J. Lequieu, and J. J. de Pablo. 2019. The free energy landscape of internucleosome interactions and its relation to chromatin fiber structure. *ACS Cent. Sci.* 5:341–348. https://doi.org/10.1021/acscentsci.8b00836.
26. Nozaki, T., R. Imai, …, K. Maeshima. 2017. Dynamic organization of chromatin domains revealed by super-resolution live-cell imaging.

Mol. Cell. 67:282–293 e287. https://doi.org/10.1016/j.molcel.2017.06.018.

27. Zhang, R., J. Erler, and J. Langowski. 2017. Histone acetylation regulates chromatin accessibility: role of H4K16 in inter-nucleosome interaction. *Biophys. J.* 112:450–459. https://doi.org/10.1016/j.bpj.2016.11.015.

28. Otterstrom, J., A. Castells-Garcia, …, M. Lakadamyali. 2019. Super-resolution microscopy reveals how histone tail acetylation affects DNA compaction within nucleosomes in vivo. *Nucleic Acids Res.* 47:8470–8484.

29. Allshire, R. C., and H. D. Madhani. 2018. Ten principles of heterochromatin formation and function. *Nat. Rev. Mol. Cell Biol.* 19:229–244. https://doi.org/10.1038/nrm.2017.119.

30. Heun, P., T. Laroche, …, S. M. Gasser. 2001. Chromosome dynamics in the yeast interphase nucleus. *Science.* 294:2181–2186. https://doi.org/10.1126/science.1065366.

31. Marshall, W. F., A. Straight, …, J. W. Sedat. 1997. Interphase chromosomes undergo constrained diffusional motion in living cells. *Curr. Biol.* 7:930–939. https://doi.org/10.1016/s0960-9822(06)00412-x.

32. Levi, V., Q. Ruan, …, E. Gratton. 2005. Chromatin dynamics in interphase cells revealed by tracking in a two-photon excitation microscope. *Biophys. J.* 89:4275–4285. https://doi.org/10.1529/biophysj.105.066670.

33. Hajjoul, H., J. Mathon, …, A. Bancaud. 2013. High-throughput chromatin motion tracking in living yeast reveals the flexibility of the fiber throughout the genome. *Genome Res.* 23:1829–1838. https://doi.org/10.1101/gr.157008.113.

34. Lucas, J. S., Y. Zhang, …, C. Murre. 2014. 3D trajectories adopted by coding and regulatory DNA elements: first-passage times for genomic interactions. *Cell.* 158:339–352. https://doi.org/10.1016/j.cell.2014.05.036.

35. Germier, T., S. Kocanova, …, K. Bystricky. 2017. Real-time imaging of a single gene reveals transcription-initiated local confinement. *Biophys. J.* 113:1383–1394. https://doi.org/10.1016/j.bpj.2017.08.014.

36. Chen, B., L. A. Gilbert, …, B. Huang. 2013. Dynamic imaging of genomic loci in living human cells by an optimized CRISPR/Cas system. *Cell.* 155:1479–1491. https://doi.org/10.1016/j.cell.2013.12.001.

37. Gu, B., T. Swigut, …, J. Wysocka. 2018. Transcription-coupled changes in nuclear mobility of mammalian cis-regulatory elements. *Science.* 359:1050–1055. https://doi.org/10.1126/science.aao3136.

38. Ma, H., L. C. Tu, …, T. Pederson. 2019. Cell cycle- and genomic distance-dependent dynamics of a discrete chromosomal region. *J. Cell Biol.* 218:1467–1477. https://doi.org/10.1083/jcb.201807162.

39. Shaban, H. A., R. Barth, and K. Bystricky. 2018. Formation of correlated chromatin domains at nanoscale dynamic resolution during transcription. *Nucleic Acids Res.* 46:e77.

40. Zidovska, A., D. A. Weitz, and T. J. Mitchison. 2013. Micron-scale coherence in interphase chromatin dynamics. *Proc. Natl. Acad. Sci. U S A.* 110:15555–15560. https://doi.org/10.1073/pnas.1220313110.

41. Bintu, B., L. J. Mateo, …, X. Zhuang. 2018. Super-resolution chromatin tracing reveals domains and cooperative interactions in single cells. *Science.* 362:eaau1783. https://doi.org/10.1126/science.aau1783.

42. Diermeier, S., P. Kolovos, …, R. Merkl. 2014. TNFα signalling primes chromatin for NF-κB binding and induces rapid and widespread nucleosome repositioning. *Genome Biol.* 15:536.

43. Lai, B., W. Gao, …, K. Zhao. 2018. Principles of nucleosome organization revealed by single-cell micrococcal nuclease sequencing. *Nature.* 562:281–285.

44. Stergachis, A. B., B. M. Debo, …, J. A. Stamatoyannopoulos. 2020. Single-molecule regulatory architectures captured by chromatin fiber sequencing. *Science.* 368:1449–1454. https://doi.org/10.1126/science.aaz1646.

45. Becker, P. B., and J. L. Workman. 2013. Nucleosome remodeling and epigenetics. *Cold Spring Harb Perspect. Biol.* 5:a017905. https://doi.org/10.1101/cshperspect.a017905.

46. Hargreaves, D. C., and G. R. Crabtree. 2011. ATP-dependent chromatin remodeling: genetics, genomics and mechanisms. *Cell Res.* 21:396–420. https://doi.org/10.1038/cr.2011.32.

47. Dultz, E., R. Mancini, …, K. Weis. 2018. Quantitative imaging of chromatin decompaction in living cells. *Mol. Biol. Cell.* 29:1763–1777. https://doi.org/10.1091/mbc.E17-11-0648.

48. Parmar, J. J., and R. Padinhateeri. 2020. Nucleosome positioning and chromatin organization. *Curr. Opin. Struct. Biol.* 64:111–118. https://doi.org/10.1016/j.sbi.2020.06.021.

49. Collepardo-Guevara, R., and T. Schlick. 2014. Chromatin fiber polymorphism triggered by variations of DNA linker lengths. *Proc. Natl. Acad. Sci. U S A.* 111:8061–8066. https://doi.org/10.1073/pnas.1315872111.

50. Clauvelin, N., P. Lo, …, W. K. Olson. 2015. Nucleosome positioning and composition modulate in silico chromatin flexibility. *J. Phys. Condens Matter.* 27:064112. https://doi.org/10.1088/0953-8984/27/6/064112.

51. Nordenskiöld, L., A. P. Lyubartsev, and N. Korolev. 2017. Coarse-grained Modeling of Nucleosomes and Chromatin. CRC Press, pp. 297–340.

52. Kepper, N., D. Foethke, …, K. Rippe. 2008. Nucleosome geometry and internucleosomal interactions control the chromatin fiber conformation. *Biophys. J.* 95:3692–3705. https://doi.org/10.1529/biophysj.107.121079.

53. Muller, O., N. Kepper, …, G. Wedemann. 2014. Changing chromatin fiber conformation by nucleosome repositioning. *Biophys. J.* 107:2141–2150. https://doi.org/10.1016/j.bpj.2014.09.026.

54. Stehr, R., N. Kepper, …, G. Wedemann. 2008. The effect of internucleosomal interaction on folding of the chromatin fiber. *Biophys. J.* 95:3677–3691. https://doi.org/10.1529/biophysj.107.120543.

55. John, S., P. J. Sabo, …, J. A. Stamatoyannopoulos. 2011. Chromatin accessibility pre-determines glucocorticoid receptor binding patterns. *Nat. Genet.* 43:264–268. https://doi.org/10.1038/ng.759.

56. Beliveau, B. J., J. Y. Kishi, …, P. Yin. 2018. OligoMiner provides a rapid, flexible environment for the design of genome-scale oligonucleotide in situ hybridization probes. *Proc. Natl. Acad. Sci. U S A.* 115:E2183–E2192. https://doi.org/10.1073/pnas.1714530115.

57. Gelali, E., G. Girelli, …, M. Bienko. 2019. iFISH is a publically available resource enabling versatile DNA FISH to study genome architecture. *Nat. Commun.* 10:1636. https://doi.org/10.1038/s41467-019-09616-w.

58. Beliveau, B. J., E. F. Joyce, …, C. T. Wu. 2012. Versatile design and synthesis platform for visualizing genomes with Oligopaint FISH probes. *Proc. Natl. Acad. Sci. U S A.* 109:21301–21306. https://doi.org/10.1073/pnas.1213818110.

59. Metropolis, N., A. W. Rosenbluth, …, E. Teller. 1953. Equation of state calculations by fast computing machines. *J. Chem. Phys.* 21:1087–1092.

60. Swendsen, R. H., and J. S. Wang. 1986. Replica Monte Carlo simulation of spin glasses. *Phys. Rev. Lett.* 57:2607–2609. https://doi.org/10.1103/PhysRevLett.57.2607.

61. Katzgraber, H. G., S. Trebst, …, M. Troyer. 2006. Feedback-optimized parallel tempering Monte Carlo. *J. Stat. Mech. Theor. Exp.* 2006:P03018.

62. Stehr, R., R. Schopflin, …, G. Wedemann. 2010. Exploring the conformational space of chromatin fibers and their stability by numerical dynamic phase diagrams. *Biophys. J.* 98:1028–1037. https://doi.org/10.1016/j.bpj.2009.11.040.

63. Carlson, R. D., and D. E. Olins. 1976. Chromatin model calculations: arrays of spherical nu bodies. *Nucleic Acids Res.* 3:89–100. https://doi.org/10.1093/nar/3.1.89.

64. Mörl, M.-C., T. Zülske, …, G. Wedemann. 2019. Data formats for modelling the spatial structure of chromatin based on experimental positions of nucleosomes. *AIMS Biophys.* 6:83.

65. Davis, C. A., B. C. Hitz, …, J. M. Cherry. 2018. The Encyclopedia of DNA elements (ENCODE): data portal update. *Nucleic Acids Res.* 46:D794–D801. https://doi.org/10.1093/nar/gkx1081.

66. Mateo, L. J., S. E. Murphy, …, A. N. Boettiger. 2019. Visualizing DNA folding and RNA in embryos at single-cell resolution. *Nature.* 568:49–54. https://doi.org/10.1038/s41586-019-1035-4.

67. Sahl, S. J., S. W. Hell, and S. Jakobs. 2017. Fluorescence nanoscopy in cell biology. *Nat. Rev. Mol. Cell Biol.* 18:685–701. https://doi.org/10.1038/nrm.2017.71.

68. Kent, W. J., C. W. Sugnet, …, D. Haussler. 2002. The human genome browser at UCSC. *Genome Res.* 12:996–1006. https://doi.org/10.1101/gr.229102.

69. Finn, E. H., G. Pegoraro, …, T. Misteli. 2019. Extensive heterogeneity and intrinsic variation in spatial genome organization. *Cell.* 176:1502–1515 e1510. https://doi.org/10.1016/j.cell.2019.01.020.

70. Ashwin, S. S., K. Maeshima, and M. Sasai. 2020. Heterogeneous fluid-like movements of chromatin and their implications to transcription. *Biophys. Rev.* 12:461–468. https://doi.org/10.1007/s12551-020-00675-8.

71. Funke, J. J., P. Ketterer, …, H. Dietz. 2016. Uncovering the forces between nucleosomes using DNA origami. *Sci. Adv.* 2:e1600974.

72. Cui, K., and K. Zhao. 2012. Genome-wide approaches to determining nucleosome occupancy in metazoans using MNase-Seq. *Methods Mol. Biol.* 833:413–419. https://doi.org/10.1007/978-1-61779-477-3_24.

73. Schopflin, R., V. B. Teif, …, G. Wedemann. 2013. Modeling nucleosome position distributions from experimental nucleosome positioning maps. *Bioinformatics.* 29:2380–2386. https://doi.org/10.1093/bioinformatics/btt404.

74. Gaffney, D. J., G. McVicker, …, J. K. Pritchard. 2012. Controls of nucleosome positioning in the human genome. *PLoS Genet.* 8:e1003036. https://doi.org/10.1371/journal.pgen.1003036.

75. Mangenot, S., A. Leforestier, …, F. Livolant. 2002. Salt-induced conformation and interaction changes of nucleosome core particles. *Biophys. J.* 82 (1 Pt 1):345–356. https://doi.org/10.1016/S0006-3495(02)75399-X.

76. Kepper, N., R. Ettig, …, K. Rippe. 2011. Force spectroscopy of chromatin fibers: extracting energetics and structural information from Monte Carlo simulations. *Biopolymers.* 95:435–447. https://doi.org/10.1002/bip.21598.

77. Norouzi, D., and V. B. Zhurkin. 2018. Dynamics of chromatin fibers: comparison of Monte Carlo simulations with force spectroscopy. *Biophys. J.* 115:1644–1655. https://doi.org/10.1016/j.bpj.2018.06.032.

78. Ricci, M. A., C. Manzo, …, M. P. Cosma. 2015. Chromatin fibers are formed by heterogeneous groups of nucleosomes in vivo. *Cell.* 160:1145–1158. https://doi.org/10.1016/j.cell.2015.01.054.

79. Van Holde, K. E. 1989. Chromatin. Springer Science & Business Media, Heidelberg.

80. Fudenberg, G., and M. Imakaev. 2017. FISH-ing for captured contacts: towards reconciling FISH and 3C. *Nat. Methods.* 14:673–678. https://doi.org/10.1038/nmeth.4329.

81. Maya-Mendoza, A., P. Moudry, …, J. Bartek. 2018. High speed of fork progression induces DNA replication stress and genomic instability. *Nature.* 559:279–284. https://doi.org/10.1038/s41586-018-0261-5.

82. Davidson, I. F., B. Bauer, …, J. M. Peters. 2019. DNA loop extrusion by human cohesin. *Science.* 366:1338–1345. https://doi.org/10.1126/science.aaz3418.

83. Baldi, S., P. Korber, and P. B. Becker. 2020. Beads on a string-nucleosome array arrangements and folding of the chromatin fiber. *Nat. Struct. Mol. Biol.* 27:109–118. https://doi.org/10.1038/s41594-019-0368-x.

84. Zaret, K. S. 2020. Pioneer transcription factors initiating gene network changes. *Annu. Rev. Genet.* 54:367–385. https://doi.org/10.1146/annurev-genet-030220-015007.

85. Bartholomew, B. 2014. Regulating the chromatin landscape: structural and mechanistic perspectives. *Annu. Rev. Biochem.* 83:671–696. https://doi.org/10.1146/annurev-biochem-051810-093157.

86. Svaren, J., E. Klebanow, …, R. Chalkley. 1994. Analysis of the competition between nucleosome formation and transcription factor binding. *J. Biol. Chem.* 269:9335–9344.

87. Mirny, L. A. 2010. Nucleosome-mediated cooperativity between transcription factors. *Proc. Natl. Acad. Sci. U S A.* 107:22534–22539.

88. Thurman, R. E., E. Rynes, …, J. A. Stamatoyannopoulos. 2012. The accessible chromatin landscape of the human genome. *Nature.* 489:75–82. https://doi.org/10.1038/nature11232.

89. Lambert, S. A., A. Jolma, …, M. T. Weirauch. 2018. The human transcription factors. *Cell.* 172:650–665. https://doi.org/10.1016/j.cell.2018.01.029.

90. Vierstra, J., J. Lazar, …, J. A. Stamatoyannopoulos. 2020. Global reference mapping of human transcription factor footprints. *Nature.* 583:729–736. https://doi.org/10.1038/s41586-020-2528-x.

91. Rippe, K. 2021. Liquid-Liquid phase separation in chromatin. *Cold Spring Harb Perspect. Biol.* 14:a040683. https://doi.org/10.1101/cshperspect.a040683.

92. Schoenfelder, S., and P. Fraser. 2019. Long-range enhancer-promoter contacts in gene expression control. *Nat. Rev. Genet.* 20:437–455. https://doi.org/10.1038/s41576-019-0128-0.

93. Chen, H., M. Levo, …, T. Gregor. 2018. Dynamic interplay between enhancer–promoter topology and gene activity. *Nat. Genet.* 50:1296–1303.

94. Wurm, C. A., D. Neumann, …, S. Jakobs. 2010. Sample preparation for STED microscopy. *In* Live Cell Imaging. Springer, pp. 185–199.

95. Markaki, Y., D. Smeets, …, M. Cremer. 2012. The potential of 3D-FISH and super-resolution structured illumination microscopy for studies of 3D nuclear architecture: 3D structured illumination microscopy of defined chromosomal structures visualized by 3D (immuno)-FISH opens new perspectives for studies of nuclear architecture. *Bioessays.* 34:412–426. https://doi.org/10.1002/bies.201100176.

96. Solovei, I., A. Cavallo, …, T. Cremer. 2002. Spatial preservation of nuclear chromatin architecture during three-dimensional fluorescence in situ hybridization (3D-FISH). *Exp. Cell Res.* 276:10–23. https://doi.org/10.1006/excr.2002.5513.

97. Branco, M. R., and A. Pombo. 2006. Intermingling of chromosome territories in interphase suggests role in translocations and transcription-dependent associations. *PLoS Biol.* 4:e138. https://doi.org/10.1371/journal.pbio.0040138.

98. Göttfert, F., C. A. Wurm, …, S. W. Hell. 2013. Coaligned dual-channel STED nanoscopy and molecular diffusion analysis at 20 nm resolution. *Biophysical J.* 105:L01–L03.

99. Esa, A., P. Edelmann, …, C. Cremer. 2000. Three-dimensional spectral precision distance microscopy of chromatin nanostructures after triple-colour DNA labelling: a study of the BCR region on chromosome 22 and the Philadelphia chromosome. *J. Microsc.* 199 (Pt 2):96–105. https://doi.org/10.1046/j.1365-2818.2000.00707.x.

100. Lieleg, C., P. Ketterer, …, P. Korber. 2015. Nucleosome spacing generated by ISWI and CHD1 remodelers is constant regardless of nucleosome density. *Mol. Cell Biol.* 35:1588–1605. https://doi.org/10.1128/MCB.01070-14.

101. Oberbeckmann, E., N. Krietenstein, …, S. Eustermann. 2021. Genome information processing by the INO80 chromatin remodeler positions nucleosomes. *Nat. Commun.* 12:3231. https://doi.org/10.1038/s41467-021-23016-z.

102. Van Rossum, G., and F. L. Drake. 2009. Python 3 Reference Manual. Create Space, Scotts Valley, CA.

103. Klenin, K., H. Merlitz, and J. Langowski. 1998. A Brownian dynamics program for the simulation of linear and circular DNA and

other wormlike chain polyelectrolytes. *Biophys. J.* 74:780–788. https://doi.org/10.1016/S0006-3495(98)74003-2.

104. Zewdie, H. 1998. Computer simulation studies of liquid crystals: a new Corner potential for cylindrically symmetric particles. *J. Chem. Phys.* 108:2117–2133.

105. Hess, B., C. Kutzner, …, E. Lindahl. 2008. GROMACS 4: algorithms for highly efficient, load-balanced, and scalable molecular simulation. *J. Chem. Theory Comput.* 4:435–447. https://doi.org/10.1021/ct700301q.

106. Levin, Y. 2002. Electrostatic correlations: from plasma to biology. *Rep. Prog. Phys.* 65:1577.

107. Walker, D. A., B. Kowalczyk, …, B. A. Grzybowski. 2011. Electrostatics at the nanoscale. *Nanoscale.* 3:1316–1344. https://doi.org/10.1039/c0nr00698j.

108. Maffeo, C., R. Schopflin, …, R. Seidel. 2010. DNA-DNA interactions in tight supercoils are described by a small effective charge density. *Phys. Rev. Lett.* 105:158101. https://doi.org/10.1103/PhysRevLett.105.158101.

109. RStudioTeam. 2020. RStudio: Integrated Development for R. RStudio, PBC.

110. Rippe, K., R. Stehr, and G. Wedemann. 2012. Monte Carlo simulations of nucleosome chains to identify factors that control DNA compaction and access. *Rsc Biomol. Sci.* 198–235. https://doi.org/10.1039/9781849735056-00198.

Biophysical Journal, Volume *121*

Supplemental information

Differences in nanoscale organization of regulatory active and inactive human chromatin

Katharina Brandstetter, Tilo Zülske, Tobias Ragoczy, David Hörl, Miguel Guirao-Ortiz, Clemens Steinek, Toby Barnes, Gabriela Stumberger, Jonathan Schwach, Eric Haugen, Eric Rynes, Philipp Korber, John A. Stamatoyannopoulos, Heinrich Leonhardt, Gero Wedemann, and Hartmann Harz

SUPPORTING MATERIALS AND METHODS

Reagents

Dulbecco's Phosphate buffered saline (DPBS, 1x, D8537), Poly-L-lysine (P1399), Dextran Sulfate (D6001) and DAPI (D9542) were all purchased by Sigma, Germany. 10x DPBS (Gibco, 14200075) and 20x SSC (Invitrogen, AM9763) were from Thermo Fisher Scientific, USA. Furthermore 16% methanol-free and ultra-pure Formaldehyde (18814-20, Polysciences, USA), high-precision coverslips of 1.5 thickness and 18x18 mm size (LH22.1, Carl Rot, Germany), Formamide (0606, Amresco, USA), Fixogum rubber cement (Marabu, Germany) and SiR-DNA dye (SC007, Spirochrome, Switzerland) were used. MOWIOL was prepared according to (1).

Selection criteria for genomic regions used in this study

From the UCSC Genome browser (http://hgdownload.soe.ucsc.edu/goldenPath/hg38/database/), we downloaded the hg38 coordinates of centromeres, segmental duplications ("super dups"), and short tandem repeats ("RepeatMasker"). From the Gencode Genes project website, we downloaded version 37 of "basic" gene annotations for the 24 chromosomes in hg38 coordinates (ftp://ftp.ebi.ac.uk/pub/databases/gencode/Gencode_human/release_37/gencode.v37.basic.annotation.gtf.gz). From the Gencode file, we extracted the coordinates of gene bodies for genes annotated at level 1 or 2 for which at least one transcript is annotated at level 1 or 2 with a transcript support level of 1 or 2; this curated list of gene bodies comprises the "genic regions" in what follows. To assess chromatin accessibility across diverse cell and tissue types, we used the "Index" of (2) derived from DNase I hypersensitive regions called at FDR 0.1% in 733 diverse biosamples, and to assess accessibility in K-562 cells, we used regions called via the program hotspot2 (3) at FDR 0.1% from the alignment file downloadable from https://www.encodeproject.org/files/ENCFF591TEM/.

To identify inactive regions, we took all genomic regions between successive elements in the Index, and the regions between the first and last Index elements on each chromosome and their respective ends of the chromosome. From these, we subtracted centromeric and genic regions, and retained all resulting regions with widths of at least 25 kb across which over 80% of the sites are uniquely mappable by 36mers and over 80% lie outside regions of segmental duplications. From these remaining regions, we chose the one that was overlapped the least by RepeatMasker elements. This region, chr11:55,810,260-55,840,940, stood out because less than 26.9% of it is overlapped by RepeatMasker elements; over 62% of each of the remaining candidate inactive regions are overlapped by RepeatMasker.

To identify active regions, we started by partitioning each chromosome into 50-kb segments, starting at the "left" end of each chromosome, and later repeating this 50-kb partitioning with an offset of 25kb into each chromosome. We restricted the 50-kb segments to those that are at least 50% overlapped by Index elements and overlapped <35% by RepeatMasker elements and not overlapped by any segmental duplications. We further restricted these to 50-kb regions fully containing an Index element present in 732 or 733 diverse biosamples and a strong (maximally-scoring) DNase I hypersensitive region in the K-562 biosample. We ranked the remaining 50-kb segments in descending order by the percentages by which they are overlapped by Index elements and considered their genic content and the degree to

which they are overlapped by RepeatMasker elements. The region chr11:119,075,000-119,125,000 stood out for being spanned by a diverse set of genes.

In addition, the probe sets were spaced approximately 5 kb (midpoint to midpoint) from each other and, for the active region, were mostly placed on DHS peaks. The probe sets were designed to span 1.5 – 2 kb.

The probe sets were also transferred to the hg19 genome assembly by using the UCSC genome browser in order to be able to use the publicly available MNase-seq data set ENCSR000CXQ from the ENCODE project (see also Preparation and Simulation).

Sample preparation and microscopy

Oligonucleotide probes for STED microscopy. Dye conjugation was carried out post-synthesis in a pool via an NHS-ester modification reaction. Working stocks of pools of 30 oligonucleotides covering the target regions had a total concentration of 10 µM and were diluted further for experiments. For a list of all oligo probes used in this study see Table S1.

Sample preparation and fluorescence in situ hybridization (FISH). Hybridization was carried out as previously published with small adaptations (4). PBS washed K-562 cells were resuspended in a small volume of PBS at a density of 1 million cells per ml and cell suspension was applied to poly-L-lysine coated glass coverslips. Cells were fixed using an osmotically balanced and methanol-free 4% formaldehyde solution which has previously been shown to not cause detectable nuclear shrinkage (5). The following washing and permeabilization steps were carried out according to Bintu et al (4). Coverslips were then inverted onto 8 µl of hybridization solution and sealed with rubber cement. Slides were placed on a heat block set to 81°C for 3 minutes. The samples were incubated at 37°C overnight (16 – 20 h). This protocol uses low hybridization temperatures and short hybridization times which has previously been shown to only minimally disrupt chromatin structure on the nanoscale by using electron and super-resolution microscopy (5-8). Due to the directly labeled primary probes the protocol contains only washing steps on the second day. The samples were washed twice with 2x SSC for 15 minutes. Two 7-minute washes in 0.2x SSC/ 0.2% Tween-20 were carried out on a heat block at 56°C followed by one wash in 4x SSC/ 0.2% Tween-20 at RT. DNA was counterstained with DAPI (100 µg/ml in 2x SSC), followed by two more washes in 2x SSC. Coverslips were mounted on microscopic slides with MOWIOL (2.5% DABCO, pH 7.0), dried for 30 minutes and sealed with nail polish to preserve cell morphology and prevent shrinkage of cells.

Sample preparation for FISH and SiR-DNA staining. Samples were prepared the same way as for two color FISH. In this case only one probe pool (B for active and inactive region) with an ATTO 594 dye label was used for hybridization. Instead of DAPI counterstaining, the samples were stained in 2.5 µM SiR-DNA in 2x SSC for 1 h in a humid chamber. Subsequently, slides were washed two times with 2x SSC for 5 minutes. Coverslips were mounted on microscopic slides with MOWIOL (2.5% DABCO, pH 7.0), dried for 30 min and sealed with nail polish.

Fluorescence activated cell sorting (FACS) The effect of the cell cycle stage on the chromatin configuration was investigated by sorting K562 cells based on their DNA content into the G1, S and G2 phase. Cells were incubated for 30 minutes in RPMI 1640 medium (10 % FCS, 1 % strep/pen) containing 5 µg/µl Hoechst33342 (Thermo Scientific, Germany). After centrifugation cell pellets were resuspended in 0.5 % BSA/PBS and sieved (mesh size 40 µm) to remove cell clumps. Cell sorting was performed using the BD FACSAria Fusion (BD Biosciences, New Jersey) and FACS Diva 9.0.1 software. The gating of the sorter was adjusted to sort single cells based on light scattering. Fluorescent gates were set to sort G1, S and G2 phase cells based on Hoechst33342 fluorescence using a 405nm laser excitation and a 425-475nm bandpass emission filter. Subsequently, the three cell fractions were used in FISH experiments as described above.

STED microscopy for FISH two color imaging. The STED hardware was controlled with Python scripts by using the specpy interface to the microscope control software Imspector (versions 0.13 and 14.0, Abberior Instruments). To find oligoFISH spot pairs confocal dual color 50 µm x 50 µm x 5 µm (for 2D and 3D acquisitions) volumes were acquired using 100 µm pinhole, 150 nm pixel size, 250 nm z-steps, 10 µs pixel dwell time, no line accumulation and excitation laser powers of 18.8% for 594 nm and 19.3% for 640 nm. Confocal scans were investigated, points were detected with a Laplacian-of-Gaussian blob detector in both channels and nuclear regions exhibiting signals in both color channels no more than 5 pixels apart from one another were determined. At these points of interest, STED detail stacks (3 µm x 3µm x 1.4µm) were acquired. For 2D STED acquisitions, the spatial light modulator (SLM) was used to generate a 2D STED depletion pattern and stacks were acquired with 200 nm z steps, 7 planes, 20 nm pixel size, 10 µs pixel dwell time, 5x line accumulation, 100 µm pinhole, excitation laser power 53.5% for 594 nm, 53.5% for 640 nm and 29.6% for 775 nm depletion laser power. For 3D STED acquisitions, careful correction for refractive index mismatch between immersion fluid of the microscope objective and the cell is crucial. Therefore, immersion oil with a refractive index of 1.522 was used for 3D acquisitions. The SLM modulator was set to generate a 3D STED depletion pattern and stacks (3 µm x 3 µm x 1.5 µm) were imaged with 60 nm z steps, 25 planes, 45 nm pixel size, 10 µs pixel dwell time, 5x line accumulation, 100 µm pinhole, excitation laser power 53.5% for 594 nm, 53.5% for 640 nm and 29.6% for 775 nm depletion laser power. The process was repeated for the next overview scan. The focus position was updated to the plane of maximum intensity in the previous overview image to allow for overnight imaging without focus loss. By moving the stage in x and y in a spiral pattern, overview scans followed by STED detail scans were acquired until a pre-set amount of time had passed.

STED microscopy for FISH and SiR-DNA co-imaging. Image acquisitions were carried out on a 3D STED microscope system from Abberior Instruments described above using a 100x UPlanSApo 1.4 NA oil immersion objective (Olympus, Japan). The STED hardware was controlled with Python scripts as described above. To find oligoFISH spots in 594 nm confocal dual color 50 µm x 50 µm x 7 µm volumes were acquired using 100 µm pinhole, 150 nm pixel size, 10 µs pixel dwell time, no line accumulation and excitation laser powers of 18.8% for 594 nm and 19.3% for 640 nm. Confocal scans were investigated, points were detected with a Laplacian-of-Gaussian blob detector in the 594 nm channel. At these points of interest, STED detail stacks (3 µm x 3 µm x 1.4 µm, 200 nm plane spacing) were acquired using the 594 nm laser for excitation. To get the surrounding SiR-DNA signal a 15 x 15 µm (30

nm pixel size, 1 plane) field of view was acquired around the same points of interest using the 640 nm laser. By moving the stage in x and y in a spiral pattern, overview scans followed by STED detail scans were acquired until a pre-set amount of time had passed.

Measurement of the microscopic Resolution

Precision of distance measurement

As STED microscopy is not prone to chromatic aberrations (9) we implement the two-color approach to increase optical resolution (10) without chromatic correction. In order to estimate the localization precision under our imaging conditions we proceeded as follows:

1. As measuring the localization precision is challenging in cells because FISH spots bleach too quickly, we used a test sample with bleach-stable fluorescent beads (Tetra Spec, dark red, 100nm, Thermo Fischer, USA).
2. The intensity of the excitation and depletion laser was adjusted so that the images of the beads are comparable to the FISH spots in the publication. By acquiring *T*=10 consecutive 3D stacks of the same beads, image series were obtained in which the beads were localized by a Laplacian of Gaussian blob detection followed by a least squares fit of a Gaussian function.
3. We determined the relative position $x_{i,t}^{r}$ of each bead *i* relative to the center of mass of all beads in one frame *t* of the timeseries to mitigate effects of drift (which would not be present in the single-frame FISH measurements).
4. We then calculated the mean relative position of individual beads over time μx_i^r and calculated the root-mean-square deviation (*RMSD*) for each bead:

   $$RMSD_i = \sqrt{\frac{1}{T}\sum_{t=1}^{T}(x_{i,t}^{r} - \mu x_i^r)^2}$$

   In 3D depletion mode, we measure a mean *RMSD* of 5.3nm (xyz).
5. By assuming that the errors are uncorrelated the localization imprecision for the 3D distance of a pair of spots results in $\sqrt{2}\,5.3 \approx 7.5$ nm

Measurement of microscope resolution with reconstituted chromatin

The plasmid pFMP233 (11) contains 25 repeats (197-bp) of the Widom 601 sequence (25 x 601). This insert was excised by EcoRI and Xba1 digestion and purified by agarose gel electrophoresis. The ends of the 25-mer were labeled by ligation with 58 bp long oligonucleotides marked by 5-Propargylamino-dCTP-ATTO-647N (Jena Bioscience) or 5-Propargylamino-dCTP-ATTO-594 (Jena Bioscience) respectively. In the next step, the labeled 25 x 601 fragments were either used for microcopy or assembled into chromatin by salt gradient dialysis as described (12). This was done at medium assembly degree (histone: DNA mass ratio ~ 0.5) by using a mixture (~ 1:1 ratio) of labeled and unlabeled DNA. Reconstituted chromatin or labelled 25 x 601 fragments were mounted for STED microscopy on poly-D-lysin coated coverslips. After washing and fixing in 4% formaldehyde, samples were mounted for STED microscopy as described above (Fig. S3).

Modeling of reconstituted chromatin

For the 25x601 systems used for controls base pair 88 to 234 was the position of the first nucleosome. The nuclear repeat length for the fragment was 197 bp. Therefore, the nucleosomes start points were calculated as 88 + x * 197 bp and the end points as 234 + x * 197 bp with x as an integer between 0 and 24. We performed simulations with a maximal internucleosomal interaction of 4 kT and 6 kT. We estimated that configurations are uncorrelated after $2.5x10^3$ steps for the 25x601 system used for controls (s. section "Analysis of correlation of simulated configurations”). We sampled 2000 independent configurations. The histograms of the end-to-end distances seem quite symmetric (Fig. S3). Distances are in the range of 0 to 260 nm with a peak at about 140 nm. For increasing the internucleosomal interaction strength the compaction rises only by a negligible distance for this system.

Image data analysis

STED microscopy image analysis for FISH spot distances. Though the automated data acquisition process produced large numbers of images, some of these were of insufficient quality for further analysis due to poor signal to noise ratio or spot detection only in one channel caused by premature bleaching or sample drift. Therefore, supervised machine learning was used as a quality control step to automatically classify STED stacks into “good” or “bad”. An experienced scientist classified about more than two thousand sum projections of oligoFISH STED stacks as “analyzable data” or “not analyzable data”. Features extracted from the sum projections of his ground truth dataset were used to train a Random Forest classifier that could be used to automatically classify further acquisitions. All machine learning was done in Python 3 (13) using scikit-learn (ver. 0.19.1 or earlier). All acquired raw data including “good” and “bad” images can be found via DOI 10.17605/OSF.IO/ZJWXM

Detailed spot analysis was performed on the analyzable data to determine the coordinates of both FISH spots in their respective STED channels. The algorithm searched for the spot pair with the brightest signal and saved their subpixel coordinates for further statistical analysis. After a rough spot detection with a Laplacian-of-Gaussian blob detector, subpixel localization was performed by fitting a multidimensional Gaussian using the Levenberg-Marquardt algorithm. The code for handling the microscopy data and analysis is available at: https://bitbucket.org/davidhoerl/sted-oligofish-analysis.

Chromatin environment of single FISH spots. To determine the relative chromatin compaction at the FISH spot, a maximum z-projection of the FISH stack was overlaid onto the single SiR-DNA plane (scaled with bilinear interpolation to match pixel sizes). In the resulting images, the spot position and nuclear outlines were annotated by hand. To reduce out-of-focus signal, a rolling-ball (radius=50px) background subtraction was performed on the SiR channel. For each image, the quantile of the SiR intensity at the FISH spot location with respect to all pixels in the nuclear annotation (smoothed with a Gaussian blur with sigma=1px) was determined. The results were visualized as boxplots and statistical significance of differences between inactive and active loci was assessed via a two-sided Wilcoxon rank sum test.

Distance distributions of reconstituted chromatin. To measure the end-to-end distances of ATTO594 and ATTO647N end-labelled reconstituted chromatin (25x601 system), we acquired two-channel STED images of large FOVs (2D depletion pattern, 20nm pixel size). Probe endpoints in both color channels were localized by performing Laplacian-of-Gaussian blob detection followed by subpixel refinement by fitting a Gaussian function to the detected blobs. Localized spots with a peak brightness above a manually determined threshold were discarded to ignore aggregates. Furthermore, spots having a nearest neighbor closer than 250nm in the same channel were discarded for the same reason. A matching of point pairs from both channels with minimal overall distance was determined by solving a linear assignment problem. Distances above the maximum plausible length of 250nm (corresponding to stretched beads-on-a-string) were set to a large constant in the distance matrix to exclude them from further analysis. All matches with plausible lengths from the assignment result were used to compile the final distance measurements. The result is shown in Fig. S3 *C.*

Coarse-grained modeling

Elastic energies. Elastic interactions are modelled by harmonic potentials. The strength constants of the interactions are named $a_{(Y)}^{(X)}$ where *X* denotes the type of interaction (*s*=stretching, *b*=bending, *t*=torsion) and *Y* the interaction partners (DNA or nucleosome). The energy for stretching (Eq. 2) is calculated by:

$$E_{stretch} = \frac{a_Y^{(s)}}{b_i^0} (b_i - b_i^0)^2, \qquad (2)$$

where b_i is the current length and b_i^0 is the equilibrium length of the segment. The bending energy is given by (Eq. 3):

$$E_{bending} = \frac{a_Y^{(b)}}{b_i^0} \theta_i^{\,2}, \qquad (3)$$

Where θ_i is calculated from $cos(\theta_i) = \hat{B}_i \cdot \hat{u}_{i+1}$ with $\hat{B}_i$ being the equilibrium direction of the next segment and $\hat{u}_{i+1}$ its actual direction. The torsional energy (Eq. 4) is computed as:

$$E_{torsion} = \frac{a_Y^{(t)}}{b_i^0} (\alpha_i + \gamma_i - \tau_i)^2, \qquad (4)$$

Where the angles α_i ,and γ_i are from the Euler-transformation $(\alpha_i, \beta_i, \gamma_i)$ from the local coordinate system from segment *i* to segment *i+1*. The angle τ_i is the intrinsic twist (14).

Internucleosomal interaction. The internucleosomal interaction is described by a shifted 12-6 Lennard-Jones (Eq. 5) potential

$$E_{internuc} = 4\varepsilon(\hat{o}_1, \hat{o}_2, \hat{r}) \left[\left(\frac{\sigma_0}{|\vec{r}| - \sigma(\hat{o}_1, \hat{o}_2, \hat{r}) + \sigma_0} \right)^{12} - \left(\frac{\sigma_0}{|\vec{r}| - \sigma(\hat{o}_1, \hat{o}_2, \hat{r}) + \sigma_0} \right)^{6} \right], (5)$$

where $\hat{o}_1$ and $\hat{o}_2$ denote the orientation of the nucleosome and $\vec{r}$ the distance between the centers of the nucleosomes. The shape of the nucleosome and the spatial dependency of the internucleosomal interaction strength is modelled by ε (Eq. 6) and σ (Eq. 7) depending of $\hat{o}_1, \hat{o}_2$ and $\hat{r}$. This is implemented by a series expansion in S-functions (15):

$\sigma(\hat{o}_1, \hat{o}_2, \hat{r}) = \sigma_0[\sigma_{000}S_{000} + \sigma_{cc2}(S_{202} + S_{022}) + \sigma_{220}S_{220} + \sigma_{222}S_{222} + \sigma_{224}S_{224}]$, (6)

and

$\varepsilon(\hat{o}_1, \hat{o}_2, \hat{r}) = \varepsilon[\varepsilon_{000}S_{000} + \varepsilon_{cc2}(S_{202} + S_{022}) + \varepsilon_{220}S_{220} + \varepsilon_{222}S_{222} + \varepsilon_{224}S_{224}]$, (7)

The expansion coefficients were chosen to match the spatial dimensions of the nucleosome and data from force spectroscopy experiments (16-18).

DNA-Nucleosome excluded volume. The volume of DNA segments is approximated by spheres. The minimal distance d between the center of DNA sphere and a spherocylinder describing the nucleosomes is computed. The excluded volume energies $E_{DNA-Nuc}$ is described as the sum of the individual excluded volume energies $E'_{DNA-Nuc}$ (Eq. 8) computed for DNA sphere and the volume of the nucleosome:

$$E'_{DNA-Nuc} = \begin{cases} 0 & if\ d \geq r_n + r_d \\ k(d - r_n - r_d)^{12} & else \end{cases}, \quad (8)$$

with $r_n = (5.5/2)$ nm and $r_d = 1.2\ nm$.

Electrostatic energy of linker DNA. A DNA segment is modelled by a chain c of charged spheres The GROMACS unit system was used which is based on nm, ps, K, electron charge (e) and atomic mass unit (u) (19).

The electrostatic energy of two spheres with charge q_1 and q_2 and radius a separated by a center-to-center distance r can be approximated by the electrostatic part of the Derjaguin-Landau-Verwey-Overbeek theory (20,21) as (Eq. 9)

$$E_{el}(r) = \frac{1}{4\pi\varepsilon\varepsilon_0} q_1 q_2 \left(\frac{e^{\kappa\alpha}}{1+\kappa\alpha}\right)^2 \frac{e^{-\kappa r}}{r}, \quad (9)$$

With κ being the inverse Debye length (Eq. 10) calculated by:

$$\kappa^2 = \frac{2e_c^2 \rho N_A}{\varepsilon\varepsilon_0 k_B T}, \quad (10)$$

For the values listed in Table S2 κ yields $\kappa = 1.0387\ nm^{-1}$ which corresponds to a Debye length of $\lambda_D = \kappa^{-1} = 0.96\ nm$.

The charge of a DNA segment is given by $q = \nu d$, with ν being the nominal line charge density ($-2/0.34\ e_c\ nm^{-1}$) and d the length of the DNA represented by the sphere. The line charge density ν of the DNA must be adapted to the effective charge density (Eq. 11) $\nu *$

$$\nu *= \nu\chi_{CR}\chi_{PBS}, \quad (11)$$

Where χ_{CR} is the charge adaptation factor and χ_{PBS} accounts for the geometry of subsequent overlapping beads and for deviations due to using an approximation instead of the exact Poisson-Boltzmann (PB) equation (22). Here, we use for χ_{CR} a value of 0.42 as derived in (22). The adaptation factor χ_{PBS} was determined by relating this potential to previous description as cylindrical segments (22).

Preparation and simulation. For the preparation of the simulation data we first selected an appropriate human genome dataset (MNAse-seq of K-562 cells from the ENCODE project ENCSR000CXQ (23,24)) in BigWig format (ENCFF000VNN). Next, we converted this file into the WIG-Format applying the BigWig2Wig-tool and finally in a BED format by a simple awk-script. Reads from chromosome 11 were extracted applying another simple UNIX-awk-script. In order to avoid false positive nucleosome positions blacklisted regions were filtered out (https://www.encodeproject.org/files/ENCFF001TDO/). Best nucleosome positions were determined with NucPosSimulator (25) generating a BED file containing the nucleosome positions and the occupancy, i.e. the number of read centers counted per base pair, smoothed with a Gaussian kernel and normalized. For identifying the least probable nucleosome the mean occupancy values of the 147 bp regions classified as nucleosomes by NucPosSimulator were determined and sorted. After removing the chosen number of nucleosomes with the smallest values, we generated a nucleosome chain with liker lengths as in the region and performed computer simulations (26). In order to incorporate effects of surrounding chromatin nucleosomes 20 kb were included at both sites of both investigated regions. The simulations were carried out on the linux cluster in Stralsund and the North German Supercomputing Alliance (HLRN) in Berlin.

Calculation of nucleosome repeat length. The nucleosome repeat length (NRL) of whole chromosome 11 was determined analyzing the chr11 BED-file as described in the previous section. In a preparatory step nucleosome positions for the whole chromosome 11 were determined applying NucPosSimulator. From resulting sorted paired end nucleosome reads the repeat length between adjacent nucleosomes was calculated by subtracting the last base pair to the first base pair of the following nucleosome read. The average NRL a sliding window was calculated for a window size of 30000 bp. From this dataset windows with less than 3 nucleosomes e.g. in the centromere were removed applying filter-function from R package "dplyr" (filter(dataset(`#Nucs`!=3))). The developed script (plotNRL.R) is published in a codeocean.com capsule (https://codeocean.com/capsule/8421512/tree/v2).

Analysis of correlation of simulated configurations

Maximum value of internucleosomal interaction energy 4 kT: We performed 10 x 10^6 Monte Carlo steps per replica after simulated annealing utilizing about 10^3 core hours for each system on the HLRN supercomputer. For the analysis of autocorrelation, we performed a test simulation where we saved every 25 Monte Carlo steps. We computed the autocorrelation of the end-to-end distances and the energies and concluded that the correlation length between configurations is about 800 steps. To be on the safe side we considered configurations uncorrelated after 10x10^3 steps. For equilibration we excluded the first about 10^5 steps after simulated annealing from the analysis. The exact values are given in table S3 in supplemental material. Therefore, we generated nearly 1000 uncorrelated configurations in the lowest temperature we used to calculate the distance distribution plots.

Maximum value of internucleosomal interaction energy 6 kT: We performed 90 x 10^6 steps per replica after simulated annealing utilizing about 30x10^3 core hours for each system on the HLRN supercomputer. For the analysis of autocorrelation, we performed a test simulation where we saved every 75 Monte Carlo steps. We computed autocorrelation the end-to-end distances and the energies, and we concluded that the correlation length between configurations is about 2.5 x10^3 steps. To be on

the safe side we considered configurations uncorrelated after 20×10^3 steps. We excluded the first half, 60×10^6 steps, from the analysis for equilibration. Therefore, we generated 2000 uncorrelated configurations in the lowest temperature we used to calculate the distance distribution plots.

The 25x601 system contains only 25 nucleosomes which is only 10 % of the 251 nucleosomes of the other simulations. We estimated, that 2.5×10^3 steps, a quarter of the steps used for the active region, are more than enough for considering two configurations as uncorrelated.

Statistical analysis

Statistics and reproducibility. No statistical method was used to predetermine sample size. Investigators were not blinded during the experiments and when assessing the outcome. For each experiment, data were collected from at least three independent biological replicates.

Plots in Fig. 2-5 and Fig. S4 and S5 were generated using ggplot2 in R Studio (ver. 1.3.1056) (27). Significance levels were always tested by a non-parametric two-sided Wilcoxon rank sum test and a Bonferroni-Holm correction was used to avoid errors through multiple testing when applicable. Data in Fig. 2-3 and Fig. S5 are represented as boxplots where the middle line indicates the median, the lower and upper hinges correspond to the 25% and 75% quartiles, the upper whisker extends to the largest value no further than 1.5 x IQR (inter-quartile range) from the hinge and the lower whisker extends to the smallest value from the hinge at most 1.5 x IQR. The data acquisition, image processing and analysis was done in an unbiased way by automation.

SUPPORTING TABLES

TABLE S1 The genomic coordinates and sequences of the used oligonucleotide probes can be found in the separate file named *Oligonucleotide_probes.xlsx*

TABLE S2 Simulation parameters and constants

e_c	1.602 ´ 10^{-19} C	Electric charge unit
v	–2/0.34 e_c nm^{-1}	Line charge density of DNA
ρ	0.1 ´ 10^{24} mol nm^{-3}	Molarity of the monovalent solution
N_A	6.022 ´ 10^{23} mol^{-1}	Avogadro constant
ε	80	Value for the dielectric value in the solution
ε_0	$(4\pi f)^{-1}$	Dielectric constant
f	138.935 kJ nm mol^{-1} e_c^{-2}	Electric conversion factor
k_B	8.314513 ´ 10^{-3} kJ mol^{-1} K^{-1}	Boltzmann constant
a	1.2 nm	Radius of the DNA model sphere
T	295 K	Temperature of the solution
	10 nm	maximum DNA segment length
	5.5 nm	nucleosome height
	11 nm	nucleosome diameter
	4 k_BT and 6 k_BT (inactive)	ε for $E_{internuc}$
	5.5 nm	σ for $E_{internuc}$
	665	$a_{DNA}^{(s)}$
	665	$a_{NUC}^{(s)}$
	120.44	$a_{DNA}^{(b)}$
	120.44	$a_{NUC}^{(b)}$
	219.25	$a_{DNA}^{(t)}$
	782.85	$a_{NUC}^{(t)}$
	1.2 kJ mol^{-1}	Lennard jones ε for DNA
	2.0 kJ mol^{-1}	Lennard jones σ for DNA
	S000 = 1.6957	interaction potential nucleosome s-functions
	Scc2 = –0.7641	
	S220 = –0.1480	
	S222 = –0.2582	
	S224 = 0.5112	
	E000 = 2.7206	
	Ecc2 = 6.0995	
	E220 = 3.3826	
	E222 = 7.1036	
	E224 = 3.2870	

TABLE S3 Overview over simulation steps in Monte Carlo simulations: All simulations steps (MCS = Monte Carlo steps), steps in simulated annealing (SA), steps in replica exchange (RE), The number of steps for equilibration and where the analysis starts, the estimated number of uncorrelated configurations used in the analysis, the maximal temperature in RE and the number of replicas.

	Total MCS [10^6]	MCS SA [10^6]	MCS RE [10^6]	Analysis start in MCS	Estimated #uncorrelated configurations	Maximal temperature [K]	#Replicas
Active Full	20	10	10	10120000	992	370	16
Active -1	20	10	10	10120000	992	370	16
Active -2	20	10	10	10120000	992	370	16
Active -3	20	10	10	10120000	992	370	16
Active -4	20	10	10	10120000	992	370	16
Active -5	20	10	10	10120000	992	370	16
Active Gafney	30	20	10	20040000	999	370	16
Active -5 Gafney	30	20	10	20040000	999	370	16
Active 45% H1	50	10	40	40020000	2000	450	32
Active H1	71	20	51	31040000	2000	450	32
Active -2 H1	59	20	39	39940000	2000	450	32
Inactive	20	10	10	10200000	982	370	16
Inactive H1	20	10	10	10200000	982	370	16
Inactive 6 KbT	100	10	90	72040000	2000	590	60
Inactive 6 kbT H1 70 %	100	10	90	60060000	2000	590	60
Inactive 6 KbT H1	100	10	90	60060000	2000	590	60
25x601 4KbT	15	5	10	10002500	2001	445	16
25x601 6KbT	15	5	10	10002500	2001	445	16

TABLE S4 Statistical data for 2D STED datasets

p-values for Active AB – BC – CD – DE

	**Active AB**	**Active BC**	**Active CD**
**Active BC**	0.03220	-	-
**Active CD**	0.02706	0.80123	-
**Active DE**	0.18970	0.00053	0.00053

p-values for Inactive AB – BC – CD – DE

	**Inactive AB**	**Inactive BC**	**Inactive CD**
**Inactive BC**	$3.3 * 10^{-7}$	-	-
**Inactive CD**	$5.8 * 10^{-9}$	0.451	-
**Inactive DE**	$1.2 * 10^{-12}$	0.071	0.451

p-value for all active vs. all inactive

	**Active**
**Inactive**	$<2 * 10^{-16}$

Test: Wilcoxon rank sum test with continuity correction. Correction method: Bonferroni holm correction for multiple testing. Software: R studio, Significant: $p < 0.05$.

TABLE S5 Statistical data for 3D STED datasets

p-values for Active AB – BC – CD – DE

	**Active AB**	**Active BC**	**Active CD**
**Active BC**	0.895	-	-
**Active CD**	0.138	0.076	-
**Active DE**	1.000	1.000	0.525

p-values for Inactive AB – BC – CD – DE

	**Inactive AB**	**Inactive BC**	**Inactive CD**
**Inactive BC**	$8 * 10^{-4}$	-	-
**Inactive CD**	$8.3 * 10^{-5}$	1.000	-
**Inactive DE**	$9.4 * 10^{-5}$	1.000	1.000

p-value for all active vs. all inactive

	**Active**
**Inactive**	$<2 * 10^{-16}$

Test: Wilcoxon rank sum test with continuity correction. Correction method: Bonferroni holm correction for multiple testing. Software: R studio. Significant: $p < 0.05$.

SUPPORTING FIGURES

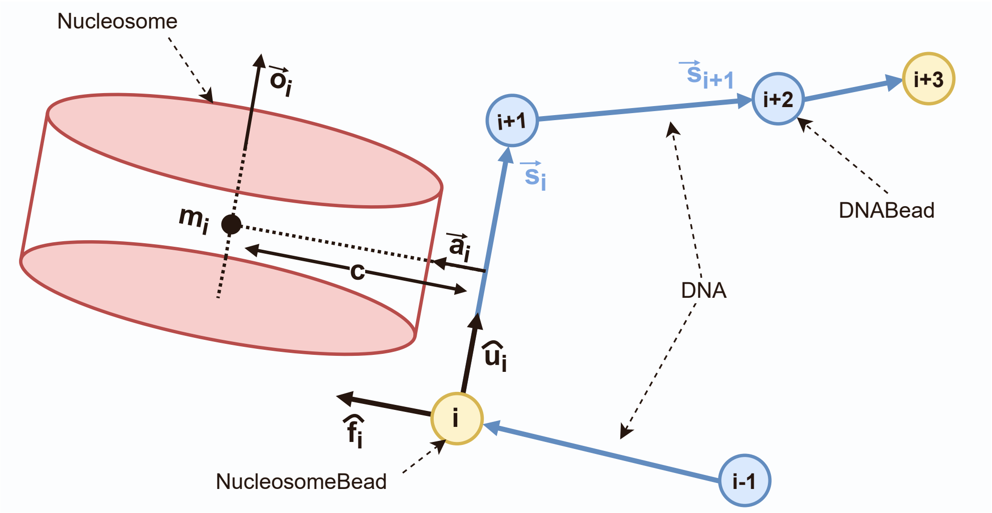

FIGURE S1 Model of a nucleosome chain. *i* represents the position of the bead in the chain, yellow circles indicate nucleosome bead positions, and blue circles indicate DNA bead positions. The nucleosome is represented by a red cylinder. The segment vector $\vec{s_i}$ points from one bead to the next bead. A local coordination system $(\hat{u}_i, \hat{v}_i, \hat{f}_i)$ (not shown) describes the orientation of a bead. Vector $\vec{a_i}$ describes the direction from the center of the segment to the nucleosome center m_i, c is its length, and vector $\vec{o_i}$ describes the orientation of the nucleosome. Vector $\vec{a_i}$ is defined by two rotations of vector $\hat{v}_i$ (i) around $\vec{u_i}$ by the angle ε (not shown), (ii) around vector $\hat{f}_i$ by the angle ϕ (not shown).

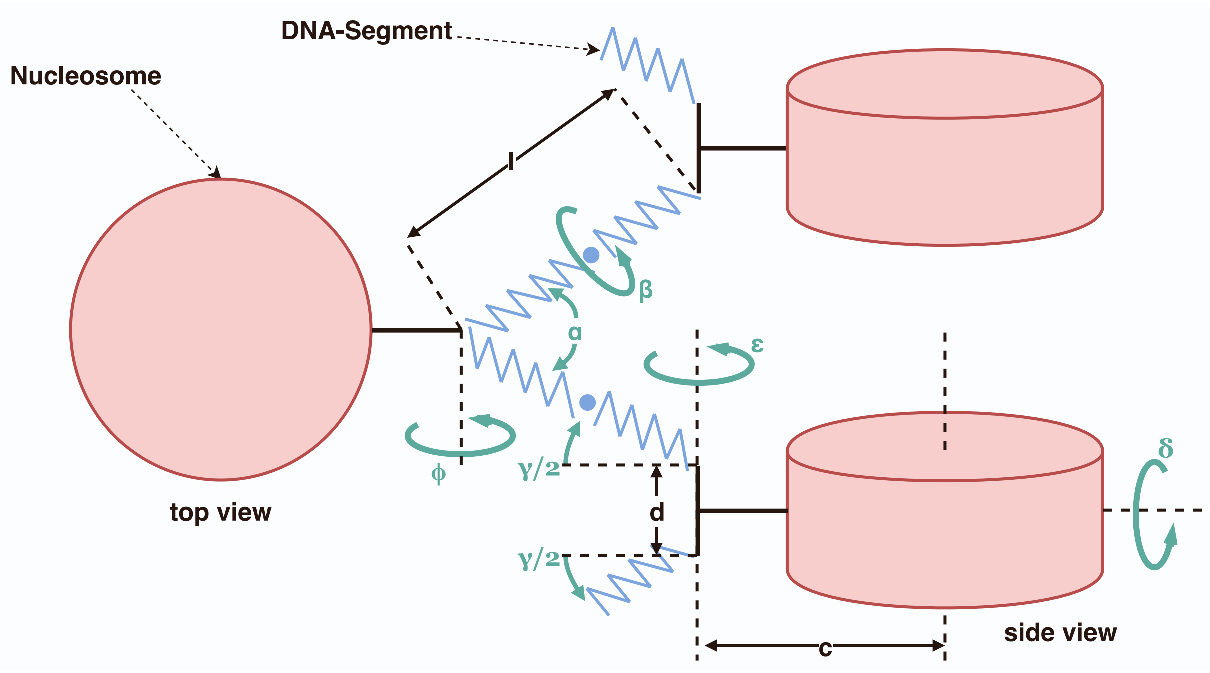

FIGURE S2 The relative orientation of the nucleosome is described by the angles $\alpha, \delta, \varepsilon, \gamma, \phi$ (modified from (28)). β is the torsional orientation of subsequent nucleosomes. l is the length of the DNA modeling the linker DNA, d the distance between the entry and the exit point of the linker DNA at the nucleosome, c the distance between the center of the nucleosome segment and the center of the oblate spherocylinder modeling of the nucleosome.

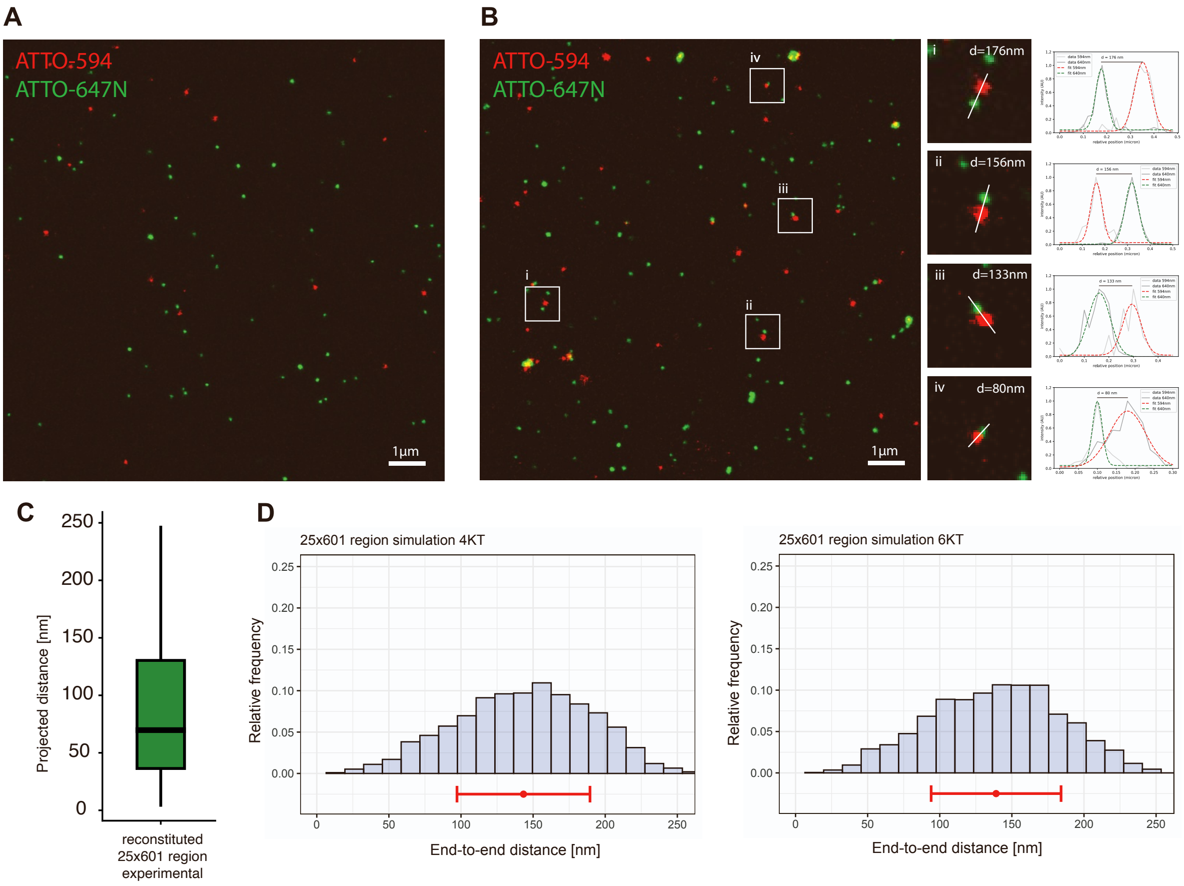

FIGURE S3 Measurement of microscope resolution with reconstituted chromatin. (*A*) Representative dual-channel STED image of ATTO-594 and ATTO-647N end-labelled DNA fragments (25x601) as described in Supporting Materials and Methods (*B*) Representative STED image of chromatin reconstituted from end-labelled 25x601 fragments. Right: magnified view of 1x1 micron around regions i-iv and line profiles. Distances were determined by fitting Gaussian functions to the line profiles in both channels and measuring the difference of the peak locations. (*C*) Box plot of the microscopically measured distances of n=230 reconstituted 25x601 fragments, median=70 nm (*D*) Distance distributions from simulations of the reconstituted 25x601 region as described in the Supporting Materials and Methods and the main text, 4KT (left) and 6KT (right). Mean value and standard deviation are shown below in red.

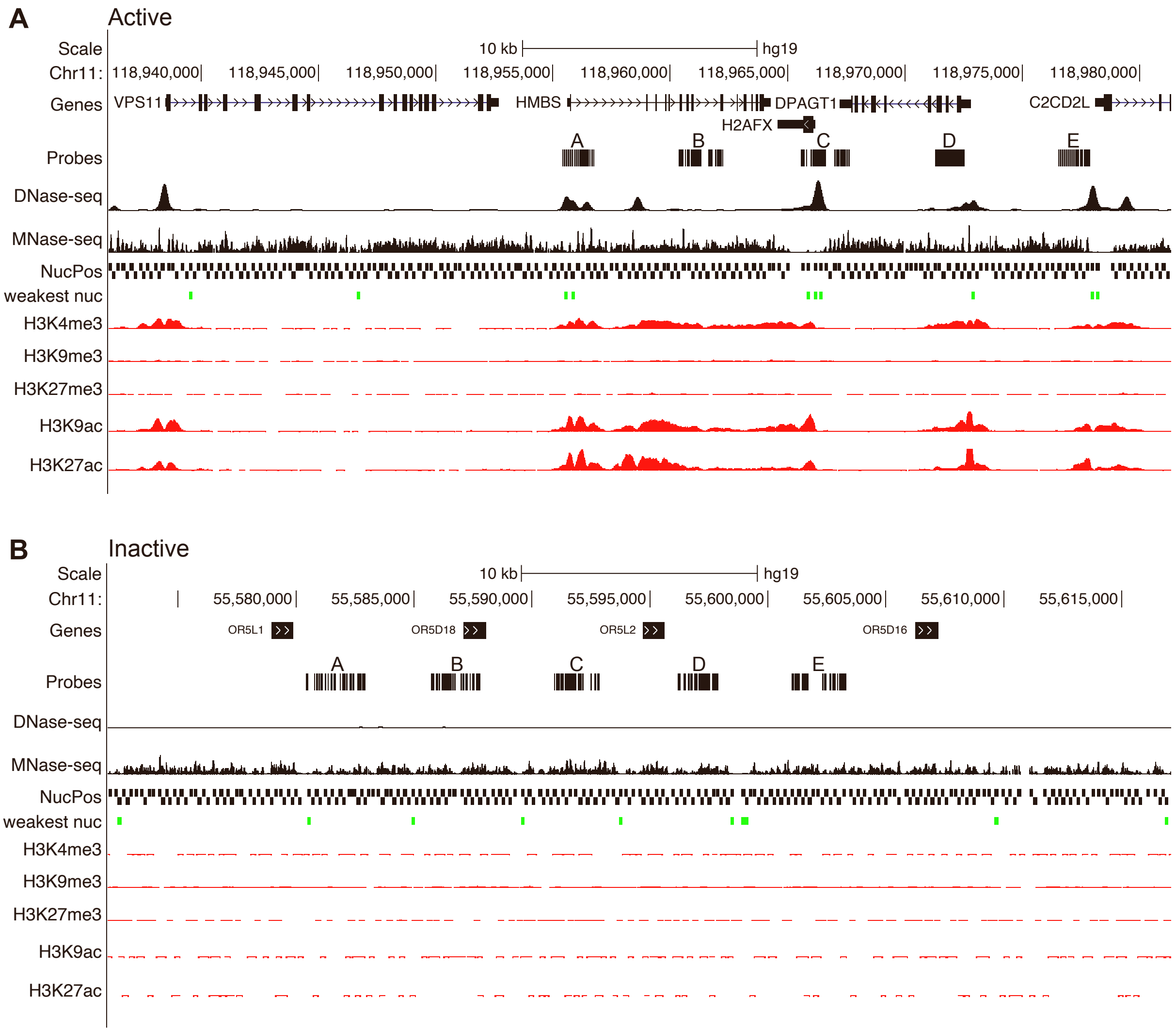

FIGURE S4 Active (*A*) and inactive (*B*) genomic regions with genes, probe sets (A-E), DNase-seq (GSM816655), MNase-seq (GSM920557), nucleosome positions from NucPosSimulator, the weakest nucleosomes calculated by NucPosSimulator, H3K4me3 (GSM733680), H3K9me3 (GSM733776), H3K27me3 (GSM733658), H3K9ac (GSM733778), H3K27ac (GSM733656). Tracks show that inactive region has almost no histone modifications while the active region contains active marks like H3K4me3, H3K9ac and H3K27ac. Notably, most of the weakest nucleosomes for the active region are located at DNase I hypersensitive sites. Plot was generated with the UCSC genome browser (29) (http://genome.ucsc.edu).

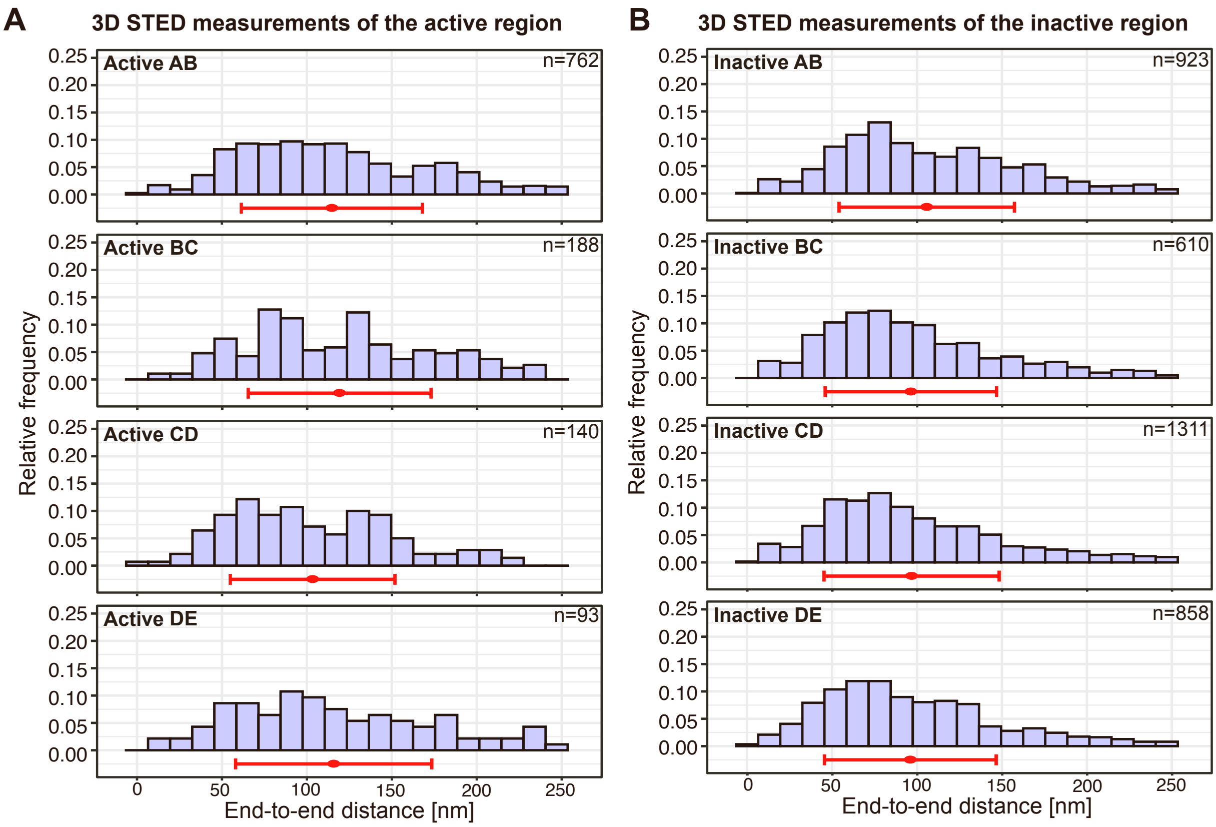

FIGURE S5 Distance histograms from 3D STED measurements for all four intervals (AB, BC, CD, DE) in active (*A*) and inactive (*B*). The mean for each histogram is indicated by the red dot and the standard deviation by the red line. N-numbers can be found next to the respective histogram (for statistical data, see Table S4).

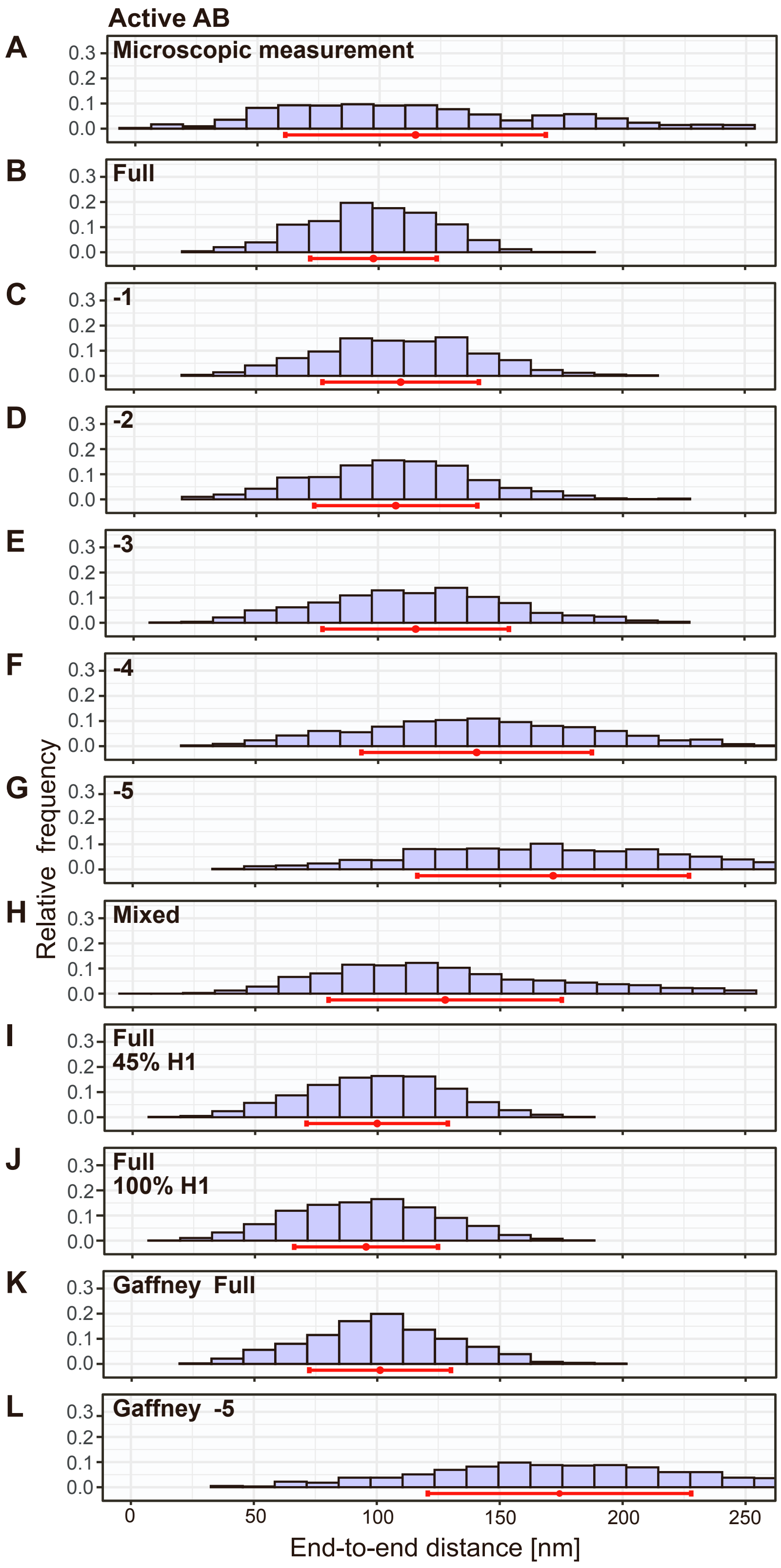

FIGURE S6 Distance distributions from simulations of the of the active locus. (*A-H*) same data as in Fig. 4 of the manuscript. As control the full locus was simulated in the presence of (*I*) 45% and (*J*) 100% H1 binding. Here, no effect was observed. (*K-L*) As second control simulations were performed with nucleosome positions derived from a lymphoblastoid cell lines (30). (*K*) all nucleosomes, (*L*) 5 nucleosomes with lowest occupancy replaced by naked DNA. Again, no effect was observed.

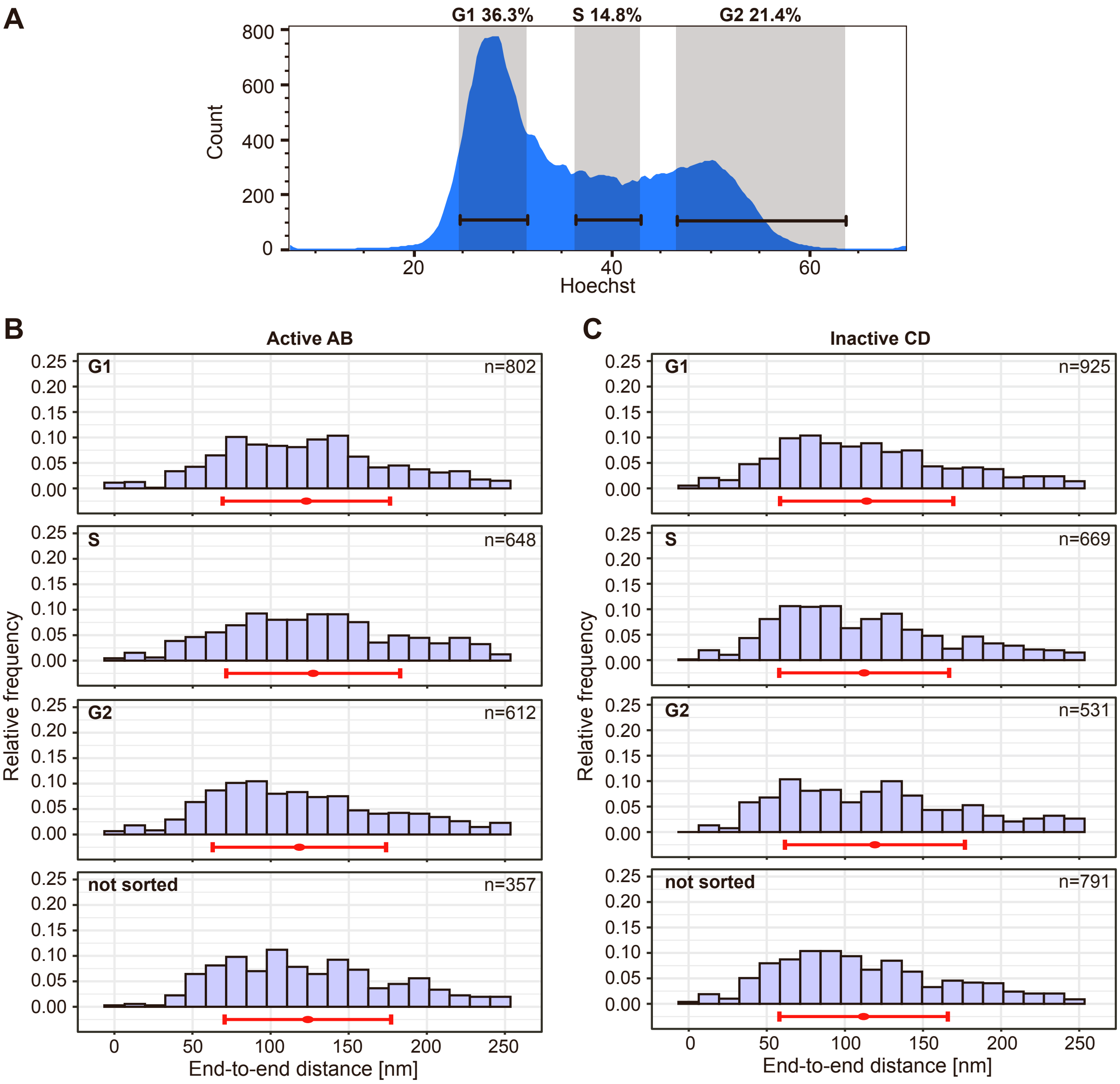

FIGURE S7 Chromatin compaction in cells of different cell cycle stages. Cells were flow-sorted according to their DNA content to investigate a possible influence of the cell cycle on the microscopically measured distance distribution of FISH spots 5 kb apart (*A*) Representative sorting profile of Hoechst-stained K562 cells, gate settings and percentage of cells in each cell cycle phase. (*B*) Distance distribution in active region AB for G1, S, G2 phase and unsorted control cells (6 replicates). (*C*) Histograms for the inactive region CD according to (*B*) using data from 4 replicates. Mean and standard deviation are shown in red under each histogram, n number of cells analyzed. Extended DNA configurations are also found in G1 phase, arguing against an effect of replication on the broadening of the histogram.

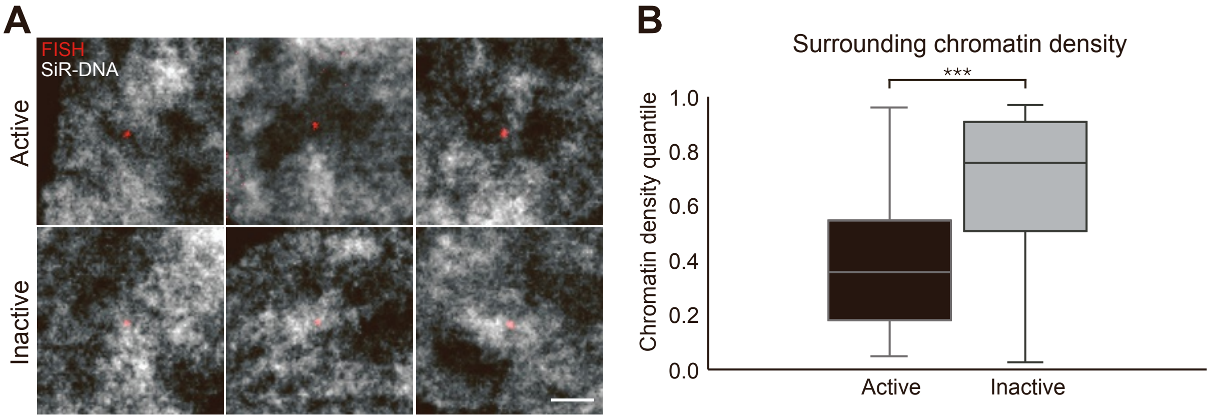

FIGURE S8 Chromatin environment of the active and inactive region. (*A*) Representative images for the active (upper row) and inactive (lower row) region labeled with one FISH probe set (red) and chromatin labeled with SiR-DNA (gray). Scale bar = 1 µm. (*B*) Chromatin density quantile for active (black, n=43) and inactive (gray, n=38) differ significantly (two-sided Wilcoxon rank sum test, $p = 9.890 * 10^{-6}$). Inactive region is embedded in higher density chromatin, while active chromatin is surrounded by lower density chromatin.

SUPPORTING REFERENCES

1. Wurm, C. A., D. Neumann, R. Schmidt, A. Egner, and S. Jakobs. 2010. Sample preparation for STED microscopy. In Live cell imaging. Springer, pp. 185-199.
2. Meuleman, W., A. Muratov, E. Rynes, J. Halow, K. Lee, D. Bates, M. Diegel, D. Dunn, F. Neri, A. Teodosiadis, A. Reynolds, E. Haugen, J. Nelson, A. Johnson, M. Frerker, M. Buckley, R. Sandstrom, J. Vierstra, R. Kaul, and J. Stamatoyannopoulos. 2020. Index and biological spectrum of human DNase I hypersensitive sites. *Nature*. 584(7820):244-251, doi: 10.1038/s41586-020-2559-3.
3. John, S., P. J. Sabo, R. E. Thurman, M. H. Sung, S. C. Biddie, T. A. Johnson, G. L. Hager, and J. A. Stamatoyannopoulos. 2011. Chromatin accessibility pre-determines glucocorticoid receptor binding patterns. *Nat Genet*. 43(3):264-268, doi: 10.1038/ng.759.
4. Bintu, B., L. J. Mateo, J. H. Su, N. A. Sinnott-Armstrong, M. Parker, S. Kinrot, K. Yamaya, A. N. Boettiger, and X. Zhuang. 2018. Super-resolution chromatin tracing reveals domains and cooperative interactions in single cells. *Science*. 362(6413):eaau1783, doi: 10.1126/science.aau1783.
5. Boettiger, A. N., B. Bintu, J. R. Moffitt, S. Wang, B. J. Beliveau, G. Fudenberg, M. Imakaev, L. A. Mirny, C. T. Wu, and X. Zhuang. 2016. Super-resolution imaging reveals distinct chromatin folding for different epigenetic states. *Nature*. 529(7586):418-422, doi: 10.1038/nature16496.
6. Markaki, Y., D. Smeets, S. Fiedler, V. J. Schmid, L. Schermelleh, T. Cremer, and M. Cremer. 2012. The potential of 3D-FISH and super-resolution structured illumination microscopy for studies of 3D nuclear architecture: 3D structured illumination microscopy of defined chromosomal structures visualized by 3D (immuno)-FISH opens new perspectives for studies of nuclear architecture. *Bioessays*. 34(5):412-426, doi: 10.1002/bies.201100176.
7. Solovei, I., A. Cavallo, L. Schermelleh, F. Jaunin, C. Scasselati, D. Cmarko, C. Cremer, S. Fakan, and T. Cremer. 2002. Spatial preservation of nuclear chromatin architecture during three-dimensional fluorescence in situ hybridization (3D-FISH). *Exp Cell Res*. 276(1):10-23, doi: 10.1006/excr.2002.5513.
8. Branco, M. R., and A. Pombo. 2006. Intermingling of chromosome territories in interphase suggests role in translocations and transcription-dependent associations. *PLoS Biol*. 4(5):e138, doi: 10.1371/journal.pbio.0040138.
9. Göttfert, F., C. A. Wurm, V. Mueller, S. Berning, V. C. Cordes, A. Honigmann, and S. W. Hell. 2013. Coaligned dual-channel STED nanoscopy and molecular diffusion analysis at 20 nm resolution. *Biophysical journal*. 105(1):L01-L03.
10. Esa, A., P. Edelmann, G. Kreth, L. Trakhtenbrot, N. Amariglio, G. Rechavi, M. Hausmann, and C. Cremer. 2000. Three-dimensional spectral precision distance microscopy of chromatin nanostructures after triple-colour DNA labelling: a study of the BCR region on chromosome 22 and the Philadelphia chromosome. *J Microsc*. 199(Pt 2):96-105, doi: 10.1046/j.1365-2818.2000.00707.x.
11. Lieleg, C., P. Ketterer, J. Nuebler, J. Ludwigsen, U. Gerland, H. Dietz, F. Mueller-Planitz, and P. Korber. 2015. Nucleosome spacing generated by ISWI and CHD1 remodelers is constant regardless of nucleosome density. *Mol Cell Biol*. 35(9):1588-1605, doi: 10.1128/MCB.01070-14.
12. Oberbeckmann, E., N. Krietenstein, V. Niebauer, Y. Wang, K. Schall, M. Moldt, T. Straub, R. Rohs, K. P. Hopfner, P. Korber, and S. Eustermann. 2021. Genome

information processing by the INO80 chromatin remodeler positions nucleosomes. *Nat Commun*. 12(1):3231, doi: 10.1038/s41467-021-23016-z.

13. Van Rossum, G., Drake, F. L. 2009. Python 3 Reference Manual. *Scotts Valley, CA: Create Space*.
14. Klenin, K., H. Merlitz, and J. Langowski. 1998. A Brownian dynamics program for the simulation of linear and circular DNA and other wormlike chain polyelectrolytes. *Biophys J*. 74(2 Pt 1):780-788, doi: 10.1016/S0006-3495(98)74003-2.
15. Zewdie, H. 1998. Computer simulation studies of liquid crystals: A new Corner potential for cylindrically symmetric particles. *The Journal of chemical physics*. 108(5):2117-2133.
16. Stehr, R., N. Kepper, K. Rippe, and G. Wedemann. 2008. The effect of internucleosomal interaction on folding of the chromatin fiber. *Biophys J*. 95(8):3677-3691, doi: 10.1529/biophysj.107.120543.
17. Stehr, R., R. Schopflin, R. Ettig, N. Kepper, K. Rippe, and G. Wedemann. 2010. Exploring the conformational space of chromatin fibers and their stability by numerical dynamic phase diagrams. *Biophys J*. 98(6):1028-1037, doi: 10.1016/j.bpj.2009.11.040.
18. Kepper, N., R. Ettig, R. Stehr, S. Marnach, G. Wedemann, and K. Rippe. 2011. Force spectroscopy of chromatin fibers: extracting energetics and structural information from Monte Carlo simulations. *Biopolymers*. 95(7):435-447, doi: 10.1002/bip.21598.
19. Hess, B., C. Kutzner, D. van der Spoel, and E. Lindahl. 2008. GROMACS 4: Algorithms for Highly Efficient, Load-Balanced, and Scalable Molecular Simulation. *J Chem Theory Comput*. 4(3):435-447, doi: 10.1021/ct700301q.
20. Levin, Y. 2002. Electrostatic correlations: from plasma to biology. *Reports on progress in physics*. 65(11):1577.
21. Walker, D. A., B. Kowalczyk, M. O. de la Cruz, and B. A. Grzybowski. 2011. Electrostatics at the nanoscale. *Nanoscale*. 3(4):1316-1344, doi: 10.1039/c0nr00698j.
22. Maffeo, C., R. Schopflin, H. Brutzer, R. Stehr, A. Aksimentiev, G. Wedemann, and R. Seidel. 2010. DNA-DNA interactions in tight supercoils are described by a small effective charge density. *Phys Rev Lett*. 105(15):158101, doi: 10.1103/PhysRevLett.105.158101.
23. Davis, C. A., B. C. Hitz, C. A. Sloan, E. T. Chan, J. M. Davidson, I. Gabdank, J. A. Hilton, K. Jain, U. K. Baymuradov, A. K. Narayanan, K. C. Onate, K. Graham, S. R. Miyasato, T. R. Dreszer, J. S. Strattan, O. Jolanki, F. Y. Tanaka, and J. M. Cherry. 2018. The Encyclopedia of DNA elements (ENCODE): data portal update. *Nucleic Acids Res*. 46(D1):D794-D801, doi: 10.1093/nar/gkx1081.
24. Consortium, E. P., J. E. Moore, M. J. Purcaro, H. E. Pratt, C. B. Epstein, N. Shoresh, J. Adrian, T. Kawli, C. A. Davis, A. Dobin, R. Kaul, J. Halow, E. L. Van Nostrand, P. Freese, D. U. Gorkin, Y. Shen, Y. He, M. Mackiewicz, F. Pauli-Behn, B. A. Williams, A. Mortazavi, C. A. Keller, X. O. Zhang, S. I. Elhajjajy, J. Huey, D. E. Dickel, V. Snetkova, X. Wei, X. Wang, J. C. Rivera-Mulia, J. Rozowsky, J. Zhang, S. B. Chhetri, J. Zhang, A. Victorsen, K. P. White, A. Visel, G. W. Yeo, C. B. Burge, E. Lecuyer, D. M. Gilbert, J. Dekker, J. Rinn, E. M. Mendenhall, J. R. Ecker, M. Kellis, R. J. Klein, W. S. Noble, A. Kundaje, R. Guigo, P. J. Farnham, J. M. Cherry, R. M. Myers, B. Ren, B. R. Graveley, M. B. Gerstein, L. A. Pennacchio, M. P. Snyder, B. E. Bernstein, B. Wold, R. C. Hardison, T. R. Gingeras, J. A. Stamatoyannopoulos, and Z. Weng. 2020. Expanded encyclopaedias of DNA elements in the human and mouse genomes. *Nature*. 583(7818):699-710, doi: 10.1038/s41586-020-2493-4.

25. Schopflin, R., V. B. Teif, O. Muller, C. Weinberg, K. Rippe, and G. Wedemann. 2013. Modeling nucleosome position distributions from experimental nucleosome positioning maps. *Bioinformatics*. 29(19):2380-2386, doi: 10.1093/bioinformatics/btt404.
26. Mörl, M.-C., T. Zülske, R. Schöpflin, and G. Wedemann. 2019. Data formats for modelling the spatial structure of chromatin based on experimental positions of nucleosomes. *AIMS Biophysics*. 6(3):83.
27. RStudioTeam. 2020. RStudio: Integrated Development for R. . *RStudio, PBC, Boston, MA*.
28. Rippe, K., R. Stehr, and G. Wedemann. 2012. Monte Carlo Simulations of Nucleosome Chains to Identify Factors that Control DNA Compaction and Access. *Rsc Biomol Sci*.(24):198-235, doi: 10.1039/9781849735056-00198.
29. Kent, W. J., C. W. Sugnet, T. S. Furey, K. M. Roskin, T. H. Pringle, A. M. Zahler, and D. Haussler. 2002. The human genome browser at UCSC. *Genome Research*. 12(6):996-1006, doi: 10.1101/gr.229102.
30. Gaffney, D. J., G. McVicker, A. A. Pai, Y. N. Fondufe-Mittendorf, N. Lewellen, K. Michelini, J. Widom, Y. Gilad, and J. K. Pritchard. 2012. Controls of nucleosome positioning in the human genome. *PLoS Genet*. 8(11):e1003036, doi: 10.1371/journal.pgen.1003036.
